# Filling a Gap: The Coordinatively Saturated Group 4 Carbonyl Complexes TM(CO)_8_ (TM=Zr, Hf) and Ti(CO)_7_

**DOI:** 10.1002/chem.201905552

**Published:** 2020-07-20

**Authors:** Guohai Deng, Shujun Lei, Sudip Pan, Jiaye Jin, Guanjun Wang, Lili Zhao, Mingfei Zhou, Gernot Frenking

**Affiliations:** ^1^ Department of Chemistry Collaborative Innovation Center of Chemistry for Energy Materials Shanghai Key Laboratory of Molecular Catalysis and Innovative Materials Fudan University Shanghai 200433 China; ^2^ Institute of Advanced Synthesis School of Chemistry and Molecular Engineering Jiangsu National Synergetic Innovation Center for Advanced Materials Nanjing Tech University Nanjing 211816 China; ^3^ Fachbereich Chemie Philipps-Universität Marburg Hans-Meerwein-Strasse 4 35043 Marburg Germany

**Keywords:** 18-electron rule, bonding analysis, matrix isolation spectroscopy, photodissociation spectroscopy, transition metals

## Abstract

Homoleptic Group 4 metal carbonyl cation and neutral complexes were prepared in the gas phase and/or in solid neon matrix. Infrared spectroscopy studies reveal that both zirconium and hafnium form eight‐coordinate carbonyl neutral and cation complexes. In contrast, titanium forms only the six‐coordinate Ti(CO)_6_^+^ and seven‐coordinate Ti(CO)_7_. Titanium octacarbonyl Ti(CO)_8_ is unstable as a result of steric repulsion between the CO ligands. The 20‐electron Zr(CO)_8_ and Hf(CO)_8_ complexes represent the first experimentally observed homoleptic octacarbonyl neutral complexes of transition metals. The molecules still fulfill the 18‐electron rule, because one doubly occupied valence orbital does not mix with any of the metal valence atomic orbitals. Zr(CO)_8_ and Hf(CO)_8_ are stable against the loss of one CO because the CO ligands encounter less steric repulsion than Zr(CO)_7_ and Hf(CO)_7_. The heptacarbonyl complexes have shorter metal−CO bonds than that of the octacarbonyl complexes due to stronger electrostatic and covalent bonding, but the significantly smaller repulsive Pauli term makes the octacarbonyl complexes stable.

## Graphical abstract

**Bonding discoveries**: The coordinatively saturated mononuclear carbonyl complexes of Group 4 elements, TM(CO)_8_ (TM=Zr, Hf) and Ti(CO)_7_, were prepared in the gas phase and/or in solid neon matrix. The analysis of the bonding situation shows that the formal 20‐electron complexes observe the 18‐electron rule and that Ti(CO)_8_ is unstable for the loss of one CO because of steric repulsion.

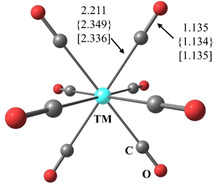

## Introduction

It has long been known that molecules possess a particular stability when their atoms have a certain number of electrons in a shell structure in which the outermost shell is wholly or partially connected to other atoms. This was formulated by Langmuir following the electron‐pair model of Lewis^1^ nearly a century ago, when he wrote: “The electrons in atoms tend to surround the nucleus in successive layers containing 2, 8, 8, 18, 18, and 32 electrons, respectively.”^2^ The counting of electrons is the basis of the octet, 18‐electron and 32‐electron rules, which are still used to explain the stability of molecules. With the advent of quantum theory, the physical basis of the electron‐counting rules was later laid when the valence shell of atoms was described in terms of s, p, d, and f atomic orbitals (AOs). Main group atoms employ their s/p valence shell for covalent bonding^3^ and obey the octet rule, transition metals use their s/p/d valence AOs in many stable complexes that are subject to the 18‐electron rule, and the 32‐electron rule is often a valid devise for the lanthanides and actinides possessing a s/p/d/f valence shell.

There are exceptions to the electron‐counting rules, which have been mysterious to chemists for some time, and which could only be explained when the quantum chemical nature of covalent bonds was understood.^4^ The most persistent problem was the so‐called hypervalent main‐group compounds, which apparently violate the octet rule. Prominent examples are pentavalent phosphorus molecules like PF_5_ and hexavalent sulfur molecules like SF_6_.^5^, ^6^ A formal electron count gives 10 valence electrons at phosphorous and 12 electrons at sulfur in the two species. However, the covalent bonds come from the interference of the electronic wave function, and the symmetry of the resulting molecular orbitals (MOs) shows that some valence electrons occupy MOs that have zero or negligible coefficients at the central atom. This was independently proposed by Rundle^7^ and Pimentel^8^ as a bonding model for molecules that appear to violate the octet rule. It is now known as the three‐center four‐electron bonding model, which was adapted by Coulson to the valence‐bond (VB) theory.^9^ The conclusion is that for the electron‐counting rules, only those electrons should be counted that actually occupy the valence AOs of the given atom. The octet rule is then valid for main group atoms having a s/p valence shell also in “hypervalent” molecules, which are better termed “hypercoordinated” compounds.

The present work deals with a similar situation for the 18‐electron rule for transition‐metal complexes. Homoleptic transition‐metal (TM) carbonyl complexes are archetypical examples for demonstrating the metal–ligand bonding and the 18‐electron rule.^10^ Mononuclear transition‐metal carbonyl complexes of groups 10, 8, and 6, such as Ni(CO)_4_, Fe(CO)_5_ and Cr(CO)_6_, are well‐known stable homoleptic carbonyl complexes that follow the 18‐electron rule. The metal−CO bonds can be straightforwardly explained with the Dewar–Chatt–Duncanson (DCD) bonding model.^11^ The early transition metals with fewer valence electrons need to bind with more than six carbonyl ligands to accomplish the 18‐electron configuration; however, high coordination may cause steric repulsion between the ligands. Therefore, the 18‐electron rule was considered to be less strict for early transition metals. V(CO)_6_ with 17 valence electrons is a highly reactive but isolable homoleptic metal carbonyl complex.^12^ The seven‐coordinate carbonyl complexes of Group 4 atoms TM(CO)_7_ (TM=Ti, Zr, Hf) formally satisfy the 18‐electron rule and were theoretically predicted to be stable species by Luo et al.^13^ Earlier matrix isolation spectroscopic studies suggest that the 16‐electron TM(CO)_6_ complexes, rather than the 18‐electron TM(CO)_7_ complexes, are formed in solid matrices.^14^, ^15^ The finding is in agreement with the idea that transition‐metal complexes TM(CO)_*n*_ with higher coordination number than *n*=6 are not stable, at least not for neutral species.

The first report of a seven‐coordinate homoleptic carbonyl was on the heptacarbonyl cation Ti(CO)_7_^+^, which was observed and investigated by using guided ion beam mass spectrometry in the gas phase.^16^ Although the bond dissociation energy of the 17‐electron Ti(CO)_7_^+^ complex is relatively small ((0.54±0.07) eV), it is stronger than expected for a ligand in the second coordination shell. Recent infrared photodissociation spectroscopic studies in the gas phase revealed that the 15‐electron TM(CO)_6_^+^ complexes, rather than the 17‐electron TM(CO)_7_^+^ complexes, should be characterized to be the fully coordinated complexes for TM=Ti, Zr, Hf.^17^ In the cases of group 3 and group 5 metals, the heavier metal cations form the expected 18‐electron complexes TM(CO)_7_^+^ (TM=Nb, Ta) and TM(CO)_8_^+^ (TM=Y, La), but the lighter Sc^+^ and V^+^ ions form only the 16‐electron complexes Sc(CO)_7_^+^ and V(CO)_6_^+^, respectively.^18^ The 18‐electron complexes Sc(CO)_8_^+^ and V(CO)_7_^+^ were predicted to be stable, and higher‐temperature experiments gave the expected 18‐electron complex V(CO)_7_^+^ for vanadium.^19^ Very recently, the 17‐electron Cr(CO)_6_^+^ cation was isolated as a stable salt compound.^20^

The situation for negatively charged metal carbonyl complexes is different. Very recently, we reported the observation of the anions TM(CO)_8_^−^ (TM=Sc, Y, La), which are isoelectronic with the neutral Group 4 complexes TM(CO)_8_ TM=Ti, Zr, Hf.^21^ Given that the valence shell of the anions has a larger radius than that of the neutral species, we were hoping that a higher coordination number than six could be achieved. Our expectation was approved, but surprisingly we observed the octacarbonyl complexes TM(CO)_8_^−^ (TM=Sc, Y, La) as coordinatively saturated complexes. The latter systems are formally 20‐electron systems when all valence electrons of the metal−CO bonds are counted. Our theoretical analysis showed that the complexes possess cubic (*O_h_*) symmetry, in which one of the metal−CO valence MOs with a_2u_ symmetry has no AO coefficient at the metal.^21^ The remaining nine MOs have AO coefficients at the metal; they can be easily associated with the TM←CO σ donation and TM→CO π back‐donation with the latter being the dominant term. Thus, the octacarbonyl complexes TM(CO)_8_^−^ (TM=Sc, Y, La) fulfill the 18‐electron rule suggested by Langmuir if only those valence electrons are counted that by symmetry bind to the metal.

A very surprising result was the recent observation by us that the heavy alkaline‐earth elements calcium, strontium, and barium can also bind eight CO ligands forming the eight‐coordinate neutral complexes M(CO)_8_ (M=Ca, Sr, Ba) in low‐temperature solid neon matrix.^22^ A theoretical study showed that the latter species also have cubic (*O_h_*) symmetry and that the metal−CO bonding exhibits the same pattern as the transition‐metal carbonyl complexes. Very recently, we even reported the observation of the isoelectronic dinitrogen complexes M(N_2_)_8_.^23^ The analysis of the bonding situation in M(CO)_8_ (M=Ca, Sr, Ba) revealed that it can be understood with the DCD model in terms of the M←CO σ donation and M→CO π back‐donation, for which the metal AOs of the latter term come from the valence d AOs.^21^ The heavy alkaline‐earth elements Ca, Sr, and Ba bind like transition metals in the octacarbonyl complexes M(CO)_8_. The complexes are formally 18‐electron systems, but the occupied valence a_2u_ MO of the cubic (*O_h_*) structures has a node at the metal atom, and thus, the complexes are actually 16‐electron systems. The degenerate e_g_ HOMO is only doubly occupied, and therefore, the complexes M(CO)_8_ (M=Ca, Sr, Ba) have a triplet ground state.^21^

With the above knowledge, we re‐investigated the question of the stability of the coordinatively saturated Group 4 carbonyl complexes, which may be realized in octacarbonyl complexes TM(CO)_8_ rather than heptacarbonyl complexes TM(CO)_7_ (TM=Ti, Zr, Hf). Here we report a combined infrared spectroscopic and theoretical study on coordinatively saturated Group 4 metal carbonyl complexes prepared in the gas phase and/or solid neon matrix. With respect to coordinative saturation of transition metals, we refer to the 18‐electron rule. The results show that although titanium only forms the Ti(CO)_6_^+^ and Ti(CO)_7_ complexes, both Zr and Hf form eight‐coordinate carbonyl neutral and positively charged adducts. The first experimental observation of the coordinatively saturated Group 4 carbonyl complexes closes a gap in the field of homoleptic transition‐metal complexes.

## Experimental Section

### Experimental methods

The Group 4 metal carbonyl cation complexes were generated in the gas phase by using a pulsed laser vaporization/supersonic‐expansion source and were studied by infrared photodissociation spectroscopy.^24^ The 1064 nm fundamental of a Nd:YAG laser was used to vaporize a rotating metal target. The metal carbonyl cation complexes were produced from the laser vaporization process in expansions of helium seeded with 5–10 % CO by using a pulsed valve (General Valve, Series 9) at 0.6–1.0 MPa backing pressure. After free expansion and cooling, the cations were skimmed into a second chamber and were pulse‐extracted and analyzed by using a time‐of‐flight mass spectrometer (TOFMS). Cations of a specific mass were mass‐selected by their flight time and decelerated. The ions were subsequently excited by a tunable IR laser in the extraction region of a second collinear TOFMS. The infrared source was generated by a KTP/KTA/AgGaSe2 optical parametric oscillator/amplifier system (OPO/OPA, Laser Vision) pumped by a Continuum Surelite EX Nd:YAG laser, providing tunable infrared light about 1.0–2.0 mJ pulse^−1^ in the range of 1500–2200 cm^−1^. The wavenumber of the OPO laser was calibrated by using the CO absorptions. When the infrared laser was on resonance with a vibrational fundamental of the ion complex, absorption took place, which resulted in the dissociation of the complex. The fragment and parent ions were reaccelerated and mass analyzed by the second TOFMS. The photodissociation spectrum was obtained by monitoring the yield of the fragment ion as a function of the dissociation IR laser wavelength and normalizing to the parent ion signal. Typical spectra were recorded by scanning the dissociation laser in steps of 2 cm^−1^ and averaging over 300 laser shots at each wavelength.

The Group 4 metal carbonyl neutral complexes were prepared by the reactions of metal atoms and carbon monoxide in solid neon and were investigated by using Fourier transform infrared absorption spectroscopy as described in detail previously.^25^ The metal atoms were prepared by pulsed laser evaporation of a rotating metal target and were co‐deposited with a dilute carbon monoxide–neon mixture (0.1–10 % CO/Ne on the basis of volume) onto a cryogenic CsI window maintained at 4 K by means of a closed‐cycle helium refrigerator. The 1064 nm fundamental of a Nd:YAG laser (Continuum, Minilite II, 10 Hz repetition rate) was used for evaporation. After 30 min of deposition, infrared spectra of the resulting samples were recorded in the transmission mode between 4000 and 450 cm^−1^ by using a Bruker Vertex 80 V spectrometer at 0.5 cm^−1^ resolution. A liquid‐nitrogen‐cooled broad‐band HgCdTe (MCT) detector was used. Bare window backgrounds, recorded prior to sample deposition, were used as references in processing the sample spectra. After the infrared spectrum of the initial deposition had been recorded, the samples were warmed up to the desired temperature and quickly re‐cooled and more spectra were taken. Alternatively, for some experiments, broad‐band photoexcitation was performed by using a high‐pressure mercury arc lamp with glass filters. The CO/Ne mixture was prepared in a stainless‐steel vacuum line by using standard manometric techniques. CO (Shanghai BOC, >99.5 %) and isotopically labelled ^13^C^16^O (ISOTEC, 99 %) and ^12^C^18^O (ISOTEC, 99 %) were used without further purification.

### Theoretical methods

The quantum chemical calculations using density functional theory (DFT) were carried out with the M06 functional developed by Truhlar and Zhao^26^ in combination with the def2‐TZVPP^27^ basis set. Comparative calculations with other functionals gave very similar results, with M06 showing the best agreement with experimental results. This level was also coupled with the D3 correction proposed by Grimme et al.^28^ This basis set uses quasirelativistic effective core potentials for 28 and 60 core electrons for Zr and Hf atoms, respectively. All these computations were carried out by using the Gaussian 16 program package.^29^ Superfine integration grid was used for the computations. Unless otherwise mentioned, all the geometries that are reported here are minima on the potential energy surfaces.

The bonding situation was studied by energy decomposition analysis (EDA)^30^ together with the natural orbitals for chemical valence (NOCV)^31^ method by using the ADF 2017.01 program package.^32^ The EDA‐NOCV^33^ calculations were performed at the M06/TZ2P^34^ level where the scalar relativistic effects were included by adopting the zeroth‐order regular approximation (ZORA).^35^ In the EDA method, the intrinsic interaction energy (Δ*Ε*_int_) between two fragments is decomposed into three energy components [Eq. (1)].

(1) $$\Delta E{}_{\mathrm{int}}=\Delta E{}_{\mathrm{elstat}}+\Delta E{}_{\mathrm{Pauli}}+\Delta E{}_{\mathrm{orb}}$$

The Δ*E*_elstat_ term represents the quasiclassical electrostatic interaction between the unperturbed charge distributions of the prepared fragments. The Pauli repulsion Δ*E*_Pauli_ is the energy change associated with the transformation from the superposition of the unperturbed electron densities of the isolated fragments to the wave function, which properly obeys the Pauli principle through explicit antisymmetrization and re‐normalization of the product wave function. The term Δ*E*_orb_ is originated from the mixing of orbitals, charge transfer, and polarization between the isolated fragments. Considering that we used a metahybrid functional for EDA‐NOCV, it gives an additional metahybrid correction Δ*E*_hybrid_. This comes from the use of Hartree–Fock exchange in the functional, which cannot be broken up, separated, and assigned to the three energy terms in Equation (1).

The combination of EDA with the NOCV method allows us to partition the total Δ*E*_orb_ term into pairwise contributions of the orbital interactions. The electron density deformation Δ*ρ_k_*(*r*), which is originated from the mixing of the orbital pairs *ψ_k_*(*r*) and *ψ*_−*k*_(*r*) of the interacting fragments in the complex, represents the amount and the shape of the charge flow due to the pairwise orbital interactions [Eq. (2)], whereas the associated orbital energy term reflects the strength of such orbital interactions [Eq. (3)]. The eigenvalues *υ_k_* provide a quantitative amount of the charge migration that is associated with each orbital interaction.

(2) $$\Delta \rho_{\mathrm{orb}}\left(r\right)=\sum_{k}\Delta \rho_{\mathrm{orb}}\left(r\right)=\sum_{k=1}^{N/2}\nu_{k}\left[-\psi_{-k}^{2}\left(r\right)+\psi_{k}^{2}\left(r\right)\right]$$

(3) $$\Delta E_{\mathrm{orb}}=\sum_{k}\Delta E_{k}^{\mathrm{orb}}=\sum_{k=1}^{N/2}\nu_{k}\left[-F_{-k,-k}^{\mathrm{TS}}+F_{k,k}^{\mathrm{TS}}\right]$$

Therefore, both qualitative (Δ*ρ*_orb_) and quantitative (Δ*E*_orb_) information of the strength of individual pairs of orbital interactions can be obtained from an EDA‐NOCV analysis. For further details on the EDA‐NOCV method and its application to the analysis of the chemical bond, some recent reviews are recommended.^36^

## Results and Discussion

### Experimental results

**Figure 1:**
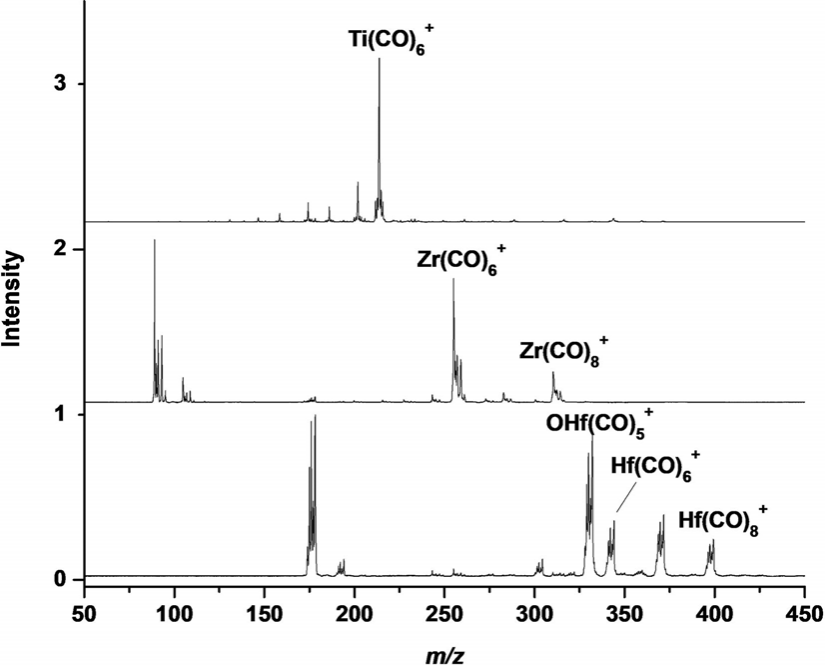
Mass spectra of Group 4 metal carbonyl cation complexes.

The mass spectra of carbonyl cation complexes of titanium, zirconium, and hafnium are shown in Figure 1. The spectra were recorded under the experimental conditions that favor the formation of coordinatively saturated mononuclear carbonyl complexes with relatively high thermal stability. The spectrum of titanium is dominated by the Ti(CO)_6_^+^ peak. The Ti(CO)_*n*_^+^ complexes of *n*>6 were barely observed. Besides the *n*=6 complexes, the TM(CO)_*n*_^+^ (TM=Zr, Hf; *n*=7, 8) complexes were also observed to have appreciable intensities in the mass spectra of zirconium and hafnium.

**Figure 2:**
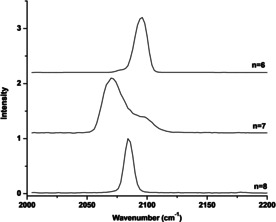
Infrared photodissociation spectra of the Zr(CO)_*n*_^+^ (*n*=6–8) complexes in the carbonyl stretching frequency region.

All these carbonyl cation complexes can dissociate by losing one CO ligand under loosely focused IR laser irradiation in the carbonyl stretching frequency region. The infrared photodissociation spectra of the Zr(CO)_*n*_^+^ cation complexes with *n*=6–8 are shown in Figure 2. The spectra of Ti(CO)_6_^+^ and Hf(CO)_*n*_^+^ (*n*=6–8) are shown in Figures S1 and S2 (Supporting Information). The spectra of the *n*=6 complexes each exhibits a single band centered at 2114 cm^−1^ for Ti, at 2096 cm^−1^ for Zr and at 2076 cm^−1^ for Hf, in full accord with previous reports.^17^ The infrared spectra of Zr(CO)_7_^+^ and Hf(CO)_7_^+^ are very similar, showing a major peak at 2071 cm^−1^ for Zr and at 2059 cm^−1^ for Hf, together with a partially resolved shoulder peak at around 2098 cm^−1^ for Zr and at 2081 cm^−1^ for Hf. The spectra of the *n*=8 complexes of zirconium and hafnium feature a single sharp band at 2085 cm^−1^ for Zr and at 2074 cm^−1^ for Hf. The observation of only one sharp band in the carbonyl stretching frequency region indicates that these *n*=8 cation complexes have high symmetry.

The mass spectrometric and infrared photodissociation spectroscopic results imply that the *n*=6 complex is the saturate‐coordinate complex for titanium, whereas both the seven‐ and eight‐coordinate complexes are formed for zirconium and hafnium. The observation of only the 6‐fold coordinate complex for titanium is in accord with the work of Duncan and Brathwaite.17a However, previous gas phase studies of Armentrout and Meyer found a stable Ti(CO)_7_^+^ complex.^16^ The seventh CO binding energy was determined to be (0.54±0.07) eV. The discrepancy was suggested to be due to the differences in ion production methods employed.17a In the Armentrout experiment, ions are thermalized by many room‐temperature collisions in a flow tube, and thus only the thermally stable ions can survive to be studied. The ions in both Duncan and co‐workers’ experiments and in our experiments are produced by pulsed laser evaporation/supersonic‐expansion source. The kinetics is the key factor that governs the formation mechanism. The Ti(CO)_6_^+^ complex has a quartet spin ground state, whereas the seven‐coordinate Ti(CO)_7_^+^ complex was predicted to have a doublet ground state.17a A spin change is therefore needed to form the seven‐coordinate complex from Ti(CO)_6_^+^. As has been discussed, the spin‐changing ligand addition reactions involve barriers, and the reaction rates are much slower than the spin‐conserving reactions.^37^

Both the *n*=7 and *n*=8 cation complexes of zirconium and hafnium are formed in our experiments, which are in apparent disagreement with the previous experiments of Duncan and co‐workers.17a In that study, they did not observe the seven‐ and eight‐coordinate complexes. This discrepancy is due to different ion production sources employed by the two laboratories. In our experiments, a Smalley‐type laser vaporization/supersonic‐expansion ion source is used.^24^ The source involves a growth channel, which can keep the laser‐ablated metal vapor and reactant gas mixtures confined to allow more condensation reactions beyond the laser vaporization point. This type of source is good for growing larger thermally stable clusters as has been demonstrated for the production of C_60_^+^.^38^ On the other hand, Duncan employed a so‐called “cutaway” laser vaporization ion source,^39^ which eliminates the growth channel beyond the laser vaporization point. The gas flow from the nozzle picks up the ablated metal and electrons forming cold metal complexes. There is no further confinement of the gas after laser vaporization.^38^ The supersonic expansion conditions make very cold ions, which may limit the production of the seven‐coordinate complex as a result of the existence of a small barrier.

**Figure 3:**
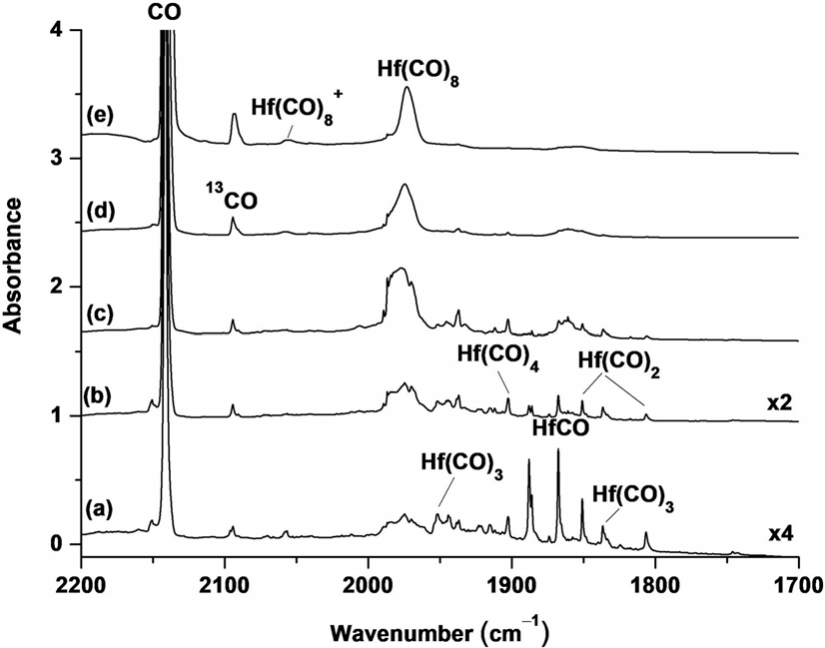
Infrared spectra in the 2200–1700 cm^−1^ region from co‐deposition of laser‐evaporated hafnium atoms with different concentrations of CO in neon. The spectra were taken after 12 K annealing. a) 0.2, b) 0.5, c) 1, d) 2.0, and e) 5.0 %.

**Figure 4:**
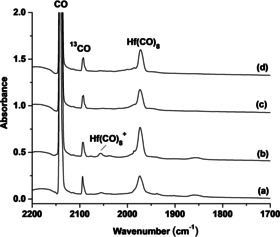
Infrared spectra in the 2200–1700 cm^−1^ region from co‐deposition of laser‐evaporated hafnium atoms with 2.5 % CO in neon. a) After 30 min of sample deposition at 4 K, b) after annealing to 12 K, c) after 15 min of UV‐visible light irradiation, and d) after another annealing to 12 K.

Besides the gas phase infrared photodissociation spectroscopic studies of the cation complexes, a series of matrix isolation experiments were performed to prepare the saturate‐coordinate Group 4 metal carbonyl complexes in solid neon matrix. The species formed were detected by using IR absorption spectroscopy. Experiments were performed by using a wide range of CO concentrations ranging from 0.1 to 10 %. The infrared spectra are summarized in Figures 3 and 4 and Figures S3–S6 (Supporting Information). The spectra from the experiments with relatively low CO concentrations (see Figure S3, Supporting Information, for hafnium) are about the same as those in previous reports.^15^ Mononuclear low‐coordinate carbonyl complexes TM(CO)_*n*_ of *n*=1–6 were formed either on sample deposition and/or on sample annealing. The relative intensities of the lower coordinated complexes decrease, whereas the relative intensities of the higher coordinated complexes increase with increasing CO concentrations (Figure 3 for Hf, Figures S4 and S5, Supporting Information, for Zr and Ti, respectively). Only two obvious bands at 1979.1 and 2066.2 cm^−1^ for zirconium (Figure S6, Supporting Information) and at 1972.5 and 2056.3 cm^−1^ for hafnium (Figure 4) were observed in the zirconium and hafnium experiments using high CO concentrations (>2 %). Both bands increase on annealing. The upper band is much weaker and is totally destroyed under UV‐visible light irradiation and cannot be recovered on subsequent annealing (Figure 4). The low band is the dominate product band presented in the spectra, which decreases on UV‐visible light irradiation and is partially recovered on subsequent annealing. In the case of titanium, multiple bands are observed to exist in the highest CO concentration experiments (Figure S5, Supporting Information). Experiments were also performed by using the isotopic‐substituted ^13^CO and C^18^O samples. The isotopic shifts are appropriate for terminal CO stretching vibrations.

The band at 1979.1 cm^−1^ for zirconium and at 1972.5 cm^−1^ for hafnium were observed to be the end‐product absorptions upon progressive annealing of the samples to temperatures of 10 to 12 K under relatively high CO concentrations. These absorptions are the dominant features in the spectra with the highest CO concentration (Figure 4 and Figure S6, Supporting Information), suggesting the assignment to the coordinatively saturate Zr(CO)_8_ and Hf(CO)_8_ complexes in solid neon matrix following the observation of the octacarbonyl cation complexes in the gas phase. The isotopic splittings in the experiments with the ^12^CO+^13^CO mixture cannot be resolved because of band overlap. Two broad bands slightly shifted from those of the pure isotopic counterparts are observed with the ^12^CO+^13^CO mixed sample (Figure S7, Supporting Information).

The much weaker band at 2066.2 cm^−1^ for zirconium and at 2056.3 cm^−1^ for hafnium can be assigned to the octacarbonyl cation complexes in solid neon. These bands are respectively 18.8 and 17.7 cm^−1^ red‐shifted from the gas phase values. These values of gas phase‐to‐matrix shift are typical for cation species.^40^

The spectra of titanium are completely different from those of zirconium and hafnium. Four bands at 1990.8, 1966.4, 1953.3, and 1942.0 cm^−1^ are observed to be the end‐product absorptions with high CO concentrations, which can be assigned to the seven‐coordinate Ti(CO)_7_ neutral complex. The bands at 1856.5 and 2098.2 cm^−1^ are photosensitive and are assigned to the Ti(CO)_6_^−^ and Ti(CO)_6_^+^ charged complexes, respectively, both of which are characterized to be coordinatively saturated complexes with absorptions at 1856 and 2114 cm^−1^, respectively, in the gas phase. The experimentally observed C−O stretching frequencies in neon matrix and in the gas phase and the calculated DFT values of the neutral and positively charged TM(CO)_*n*_^*q*^ (*n*=8, 7, 6; *q*=0, +1) species are shown in Table 1.

**Table 1:** Experimental Ne matrix and gas phase (in square brackets) infrared and calculated C−O stretching wavenumbers [cm^−1^] at the M06‐D3/def2‐TZVPP level for Group 4 metal carbonyl complexes.^[a]^ The computed intensities [km mol^−1^] are provided in parentheses.

Complex	Experimental						Calculated					
	^12^C^16^O	Δ^[b]^	^13^C^16^O	Δ^[c]^	^12^C^18^O	Δ^[c]^	^12^C^16^O	Δ^[b]^	^13^C^16^O	Δ^[c]^	^12^C^18^O	Δ^[c]^
Zr(CO)_8_ (*O_h_*, ^1^A_1g_)	1979.1	−163.9	1936.8	−42.3	1930.9	−48.2	2012.2 (t_1u_, 2607)	−130.8	1968.0	−44.2	1962.7	−49.5
Hf(CO)_8_ (*O_h_*, ^1^A_1g_)	1972.5	−170.5	1929.9	−42.6	1923.8	−48.7	2006.4 (t_1u_, 2700)	−136.6	1962.3	−44.1	1957.1	−49.3
Zr(CO)_8_^+^ (*D*_4*h*_, ^2^A_1g_)	2066.2 [2085]	−76.8 [−58]	2021.8	−44.4	2017.1	−49.1	2092.2 (a_2u_, 1654) 2095.2 (e_u_, 1638)	−50.8 −47.8	2046.1 2049.0	−46.1 −46.2	2040.9 2043.9	−51.3 −51.3
Hf(CO)_8_^+^ (*D*_4*h*_, ^2^A_1g_)	2056.3 [2074]	−86.7 [−69]	2011.6	−44.7	2007.5	−48.8	2083.2 (a_2u_, 1790) 2086.1 (e_u_, 1776)	−59.8 −56.9	2037.3 2040.1	−45.9 −46.0	2032.3 2035.2	−50.9 −50.9
Zr(CO)_7_^+^ (*C_s_*, ^2^A′)	[2098] [2071]	[−45] [−72]					2116.0 (a′′, 362) 2105.8 (a′′, 441) 2102.2 (a′′, 699) 2093.8 (a′′, 1244) 2085.7 (a′′, 1234) 2081.9 (a“, 817)	−27.0 −37.2 −40.8 −49.2 −57.3 −61.1				
Hf(CO)_7_^+^ (*C*_2*v*_, ^2^A_1_)	[2081] [2059]	[−62] [−84]					2107.9 (a_1_, 390) 2093.2 (b_2_, 732) 2087.8 (b_1_, 1838) 2078.7 (a_1_, 1321) 2075.4 (b_2_, 919)	−35.1' −49.8 −55.2 −64.3 −67.6				
Ti(CO)_7_ (*C*_3*v*_, ^1^A_1_)	1990.8 1966.4 1953.3 1942.0	−152.2 −176.6 −189.7 −201.0	1945.9 1923.2 1909.3 1899.6	−44.9 −43.2 −44.0 −42.4			2029.1 (e, 731) 2010.1 (a_1_, 1083) 1987.2 (e, 1657) 1986.1 (a_1_, 988)	−113.9 −132.9 −155.8 −156.9	1982.3 1965.5 1944.6 1941.4	−46.8 −44.6 −42.6 −44.7		
Ti(CO)_6_^+^ (*O_h_*, ^4^A_1g_)	2098.2 [2114]	−44.8 [−29]	2051.2	−47.0			2119.9 (t_1u_, 1139)	−23.1	2072.8	−47.1

[a] At M06‐D3/def2‐TZVPP level, the scaling factor is 0.958, which is obtained from the ratio of free CO stretching frequency (2143 cm^−1^) and that obtained at the M06‐D3/def2‐TZVPP level (2237 cm^−1^). [b] The frequency shift with respect to free CO stretching frequency (2143 cm^−1^). [c] The shift with respect to the C−O stretching frequency in the ^12^C^16^O isotopomer.

### Theoretical results and bonding analysis

**Figure 5:**
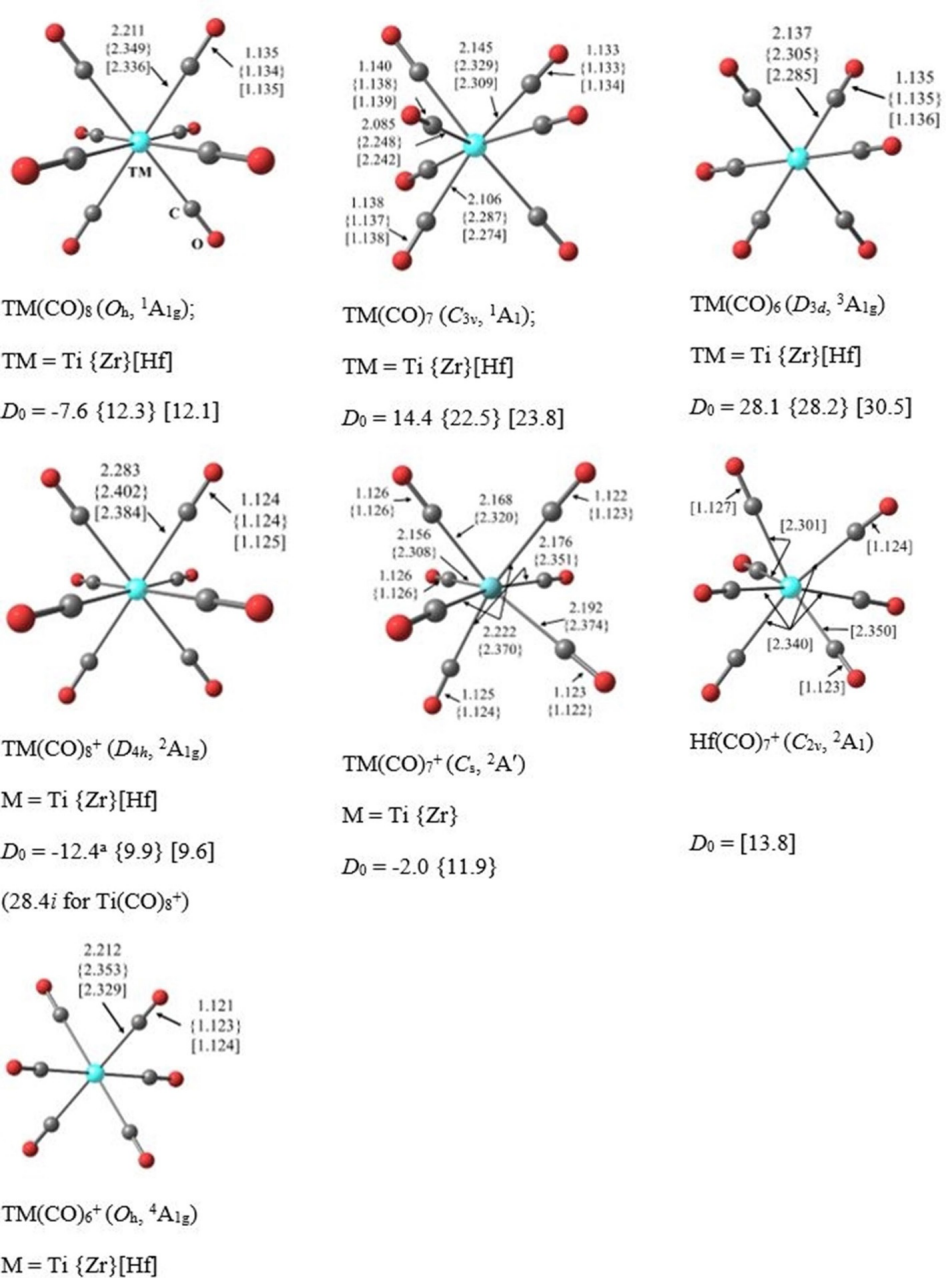
The lowest‐energy structures of TM(CO)_*n*_ and TM(CO)_*n*_^+^ (TM=Ti, {Zr}, [Hf]; *n*=6, 7, 8) at the M06‐D3/def2‐TZVPP level. Bond lengths are in Å. The zero‐point energy (ZPE)‐corrected bond dissociation energy (*D*_0_) for the loss of a single CO is given in kcal mol^−1^. [a] The BDE with respect to Ti(CO)_8_^+^ (*D*_4*h*_, ^2^A_1g_)→Ti(CO)_6_⋅⋅⋅CO^+^ (*C*_3*v*_, ^4^A_1_)+CO.

Figure 5 shows the calculated geometries of the neutral and positively charged Group 4 carbonyl complexes TM(CO)_*n*_^*q*^ (TM=Ti, Zr, Hf; *n*=6, 7, 8; *q*=0, +1). The neutral octacarbonyl complexes TM(CO)_8_ have cubic (*O_h_*) symmetry and a singlet (^1^A_1g_) electronic ground state like the isoelectronic group 3 anions TM(CO)_8_^−^ (TM=Sc, Y, La) (see Figure S8, Supporting Information, for other isomers).^21^ All Group 4 octacarbonyl complexes are minima on the potential energy surface, but the titanium complex Ti(CO)_8_ is thermodynamically unstable for the loss of one CO ligand. The dissociation reaction TM(CO)_8_→TM(CO)_7_+CO is calculated to be exothermic for TM=Ti, whereas it is endothermic for TM=Zr, Hf (Figure 5). This explains why Zr(CO)_8_ and Hf(CO)_8_ could be observed but Ti(CO)_8_ could not. The neutral Group 4 heptacarbonyl complexes TM(CO)_7_ have *C*_3*v*_ symmetry and a singlet (^1^A_1_) electronic ground state like the isoelectronic group 3 anions TM(CO)_7_^−^ (TM=Sc, Y, La) (see Figure S9, Supporting Information, for other isomers).^21^ All Group 4 heptacarbonyl complexes TM(CO)_7_ are stable with respect to CO loss yielding the hexacarbonyl complexes TM(CO)_6_, which have *D*_3*d*_ symmetry and a triplet (^3^A_1g_) ground state (Figure 5 and Figure S10, Supporting Information). The heptacarbonyl complex Ti(CO)_7_ is calculated as the highest‐coordinate neutral titanium carbonyl complex that is thermodynamically stable. Our calculated geometries of TM(CO)_7_ agree quite well with the values reported by Luo et al. for the capped octahedron (*C*_3*v*_) structures.^13^ The authors also calculated TM(CO)_7_ structures with pentagonal bipyramidal (*D*_5*h*_) and face‐capped trigonal prismatic (*C*_2*v*_) geometries, which were found to be saddle points on the potential energy surface. A second energy minimum structure with one side‐on‐bonded CO ligand and *C_s_* symmetry lies 17–22 kcal mol^−1^ above the *C*_3*v*_ structures.^13^ A comparison of the geometries of the octacarbonyl complexes with that of the heptacarbonyl complexes shows that the TM(CO)_8_ complexes have significantly longer TM−CO bonds than that of the TM(CO)_7_ adducts. This is in agreement with the calculated bond dissociation energy (BDE) for the loss of one CO ligand, which is always larger for TM(CO)_7_ than for TM(CO)_8_ (Figure 5).

Figure 5 also shows the calculated structures of the Group 4 carbonyl cations TM(CO)_*n*_^+^ (TM=Ti, Zr, Hf; *n*=6, 7, 8). Like the neutral systems, the octacarbonyl cations TM(CO)_8_^+^ are thermodynamically stable only for TM=Zr, Hf but not for TM=Ti (Figure S11, Supporting Information). The energy minimum structures TM(CO)_8_^+^ have *D*_4*h*_ symmetry and doublet (^2^A_1g_) electronic ground states. Interestingly, even the titanium heptacarbonyl cation Ti(CO)_7_^+^ is thermodynamically unstable for the loss of one CO whereas the heavier homologues Zr(CO)_7_^+^ and Hf(CO)_7_^+^ are stable (Figure 5 and Figure S12, Supporting Information). All heptacarbonyl cations TM(CO)_7_^+^, which have *C_s_* symmetry and a ^2^A′ electronic ground state for TM=Ti, Zr and *C*_2*v*_ symmetry and a ^2^A_1_ electronic ground state for TM=Hf, are energy minima. The hexacarbonyl Ti(CO)_6_^+^ is calculated as the highest‐coordinate cationic titanium carbonyl complex that is thermodynamically stable. The cations TM(CO)_6_^+^ have octahedral (*O_h_*) symmetry and a quartet (^4^A_1g_) electronic ground state (Figure 5 and Figure S13, Supporting Information).

Table 1 also shows the calculated C−O stretching modes and IR intensities of the experimentally observed neutral and positively charged carbonyl complexes with coordination numbers 6, 7, and 8. The complete list of the calculated vibrational frequencies and IR intensities of all computed species is given in Table S1 (Supporting Information). The theoretical values are scaled by 0.958, which is the ratio of the experimental value of free CO (2143 cm^−1^) and the calculated value at M06‐D3/def2‐TZVPP (2237 cm^−1^). Table 1 also gives the frequency shifts with respect to free CO and the isotope shifts of the ^13^C^16^O and ^12^C^18^O isotopomers.

The calculated C−O stretching modes are always slightly larger than the experimental values, but the trends and the frequency shifts support the assignment of the recorded modes in the gas phase and in the matrix. The neutral octacarbonyl complexes Zr(CO)_8_ and Hf(CO)_8_ have (as expected) a significantly larger red‐shift than the cations Zr(CO)_8_^+^ and Hf(CO)_8_^+^. The calculations suggest one IR‐active mode for the neutral *O_h_* complexes but two IR‐active modes for the *D*_4*h*_ cations; although the splitting of the latter modes is too small to be resolved with our equipment. The calculated isotope shifts of all octacarbonyl complexes are in excellent agreement with the experimental shifts. The experimental assignment of two frequencies for Zr(CO)_7_^+^ and Hf(CO)_7_^+^ indicates the peaks to be the rather broad signals, which are observed (Figure 2 and Figure S2, Supporting Information). The experimental spectrum agrees very well with the calculated strongly IR‐active CO stretching modes of the heptacarbonyl cations. The theoretical data suggest six frequencies for the *C_s_* structure of Zr(CO)_7_^+^ and five frequencies for the *C*_2*v*_ structure of Hf(CO)_7_^+^ within a rather narrow range of approximately 35 cm^−1^, which conforms with the recorded signals.

Table 1 also shows that the calculated IR‐active CO stretching modes of the highest‐coordinate neutral and positively charged titanium carbonyl complexes Ti(CO)_7_ and Ti(CO)_6_^+^ agree quite well with the experimental spectra. Four frequencies within a range of approximately 49 cm^−1^ are calculated and experimentally observed for the *C*_3*v*_ structure of Ti(CO)_7_, whereas only one is found for the octahedral cation Ti(CO)_6_^+^. The differences of the four IR‐active signals for Ti(CO)_7_ between theory and experiment may be partly due to dynamic effects of the fluctuate structure. Overall, the calculated IR signals and the isotope frequency shifts provide strong evidence for the assignments of the recorded spectra to the molecular species.

**Figure 6:**
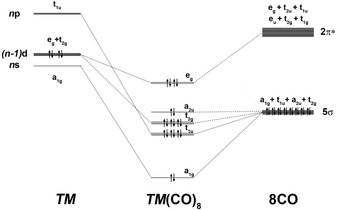
Splitting of the (*n*)s, (*n*−1)d, and (*n*)p valence orbitals of a Group 4 TM (Ti, Zr, Hf) in the cubic field (*O_h_*) of eight CO ligands and interactions with the 5σ and 2π* valence MOs of (CO)_8_.

We analyzed the metal−CO interactions in the neutral and charged complexes with the EDA‐NOCV method, which has been proven to give deep insight into the nature of the chemical bonds in metal carbonyl complexes^21^, ^22^, ^41^ and other compounds.^42^, ^43^ A particularly interesting topic concerns the question why the heavier Group 4 metals form stable octacarbonyl complexes, which are formally 20‐electron species, as highest‐coordinate complexes; although the heptacarbonyl complexes satisfy the electron demand of the metals in the 18‐electron complexes TM(CO)_7_. Figure 6 shows the correlation diagram of the (*n*)s, (*n*−1)d, and (*n*)p valence orbitals of a Group 4 transition metal TM with four valence electrons in the electronic reference state in the cubic field (*O_h_*) of eight CO ligands and the interactions with the 5σ and 2π* valence MOs of (CO)_8_. The occupied 5σ MOs of (CO)_8_ donate electronic charge into the vacant (*n*)s (a_1g_), (*n*−1)d (t_2g_), and (*n*)p (t_1u_) AOs of the metal, and the π back‐donation from the occupied metal (*n*−1)d (e_g_) AOs takes place into the vacant 2π*(e_g_) MOs of (CO)_8_. The occupied a_2u_ MO of (CO)_8_ does not interact with the valence MOs of TM because they do not have the proper symmetry. Thus, the formal 20‐electron systems TM(CO)_8_ (TM=Ti, Zr, Hf) are effectively 18‐electron complexes when only those electrons that are engaged in the metal–ligand interactions are counted.

**Figure 7:**
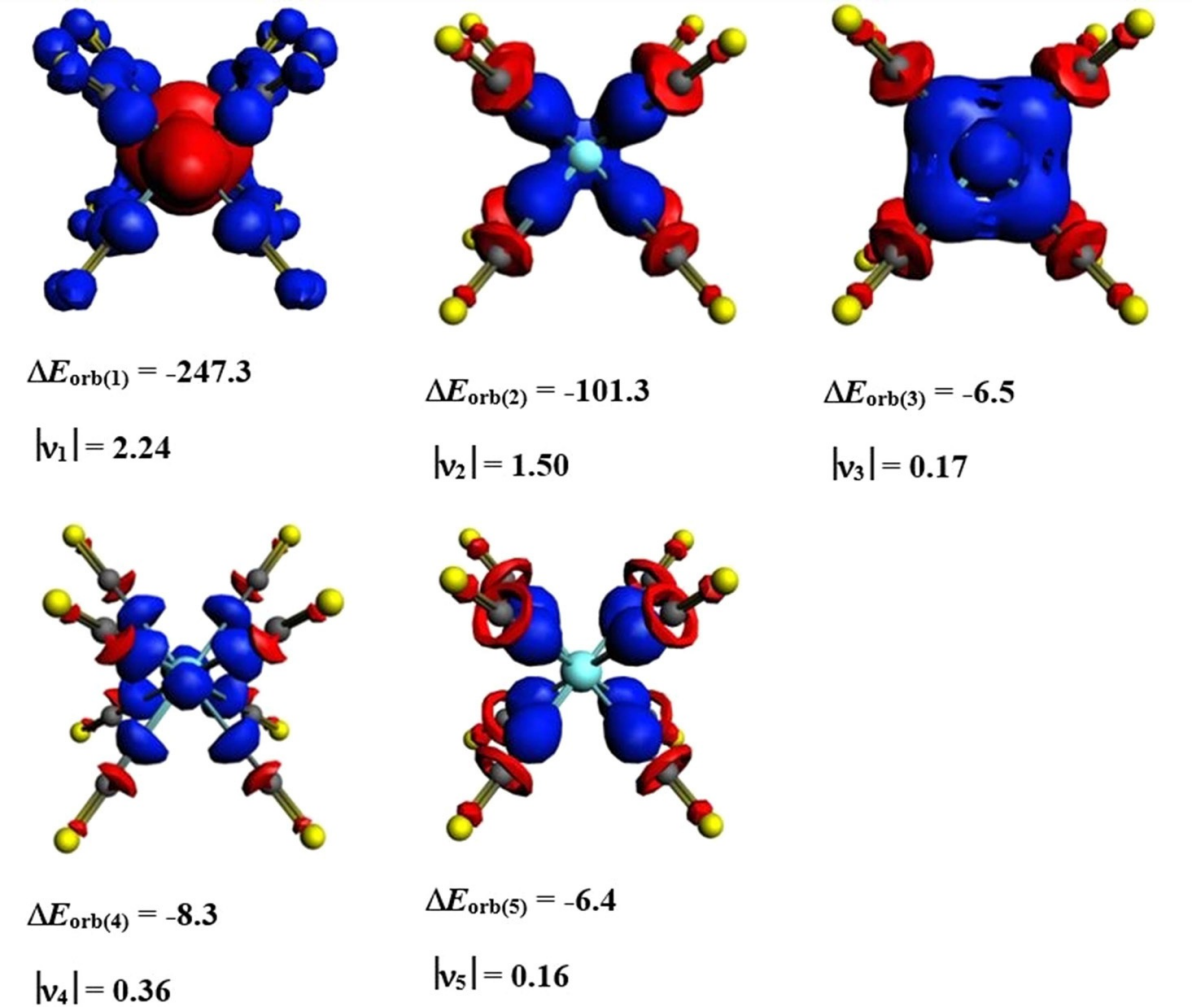
Shape of the deformation densities Δ*ρ*_(1)–(5)_, which are associated with the orbital interactions Δ*E*_orb(1)–(5)_ in Zr(CO)_8_ and eigenvalues |**ν_n_**| of the charge flow. The isosurface values are 0.002 for Δ*ρ*_(1)–(2)_ and 0.0008 for Δ*ρ*_(3)–(5)_. Only one component of the degenerate orbital interactions is shown. The color code of the charge flow is red→blue.

The numerical EDA‐NOCV results of the TM(CO)_8_ complexes are shown in Table 2. The strength of the interaction energy Δ*E*_int_ of the three metals shows the usual V‐shaped trend in which the third transition‐metal‐row atom Hf exhibits the strongest interactions due to relativistic effects, whereas the second‐row element Zr has the weakest interactions. It is interesting to note that the isoelectronic anions TM(CO)_8_^−^ (TM=Sc, Y, La) show a more regular trend for the Δ*E*_int_ values (Sc^−^>Y^−^>La^−^) in which the heaviest metal anion La^−^ has the weakest bonds.^21^ The reason for this remains to be studied. The breakdown of the orbital term Δ*E*_orb_, which provides about two‐thirds of the total TM−(CO)_8_ attraction in the Group 4 octacarbonyl complexes, into the pairwise orbital interactions shows that the major contribution comes from the [TM(d)]→(CO)_8_ π back‐donation followed by [TM(d)]←(CO)_8_ σ donation (Table 2). The two orbital terms afford >87 % of the covalent TM−(CO)_8_ bonding. The EDA‐NOCV results suggest that the most important valence orbitals of the metal atoms for the covalent bonds are the (*n*−1)d AOs. Figure 7 shows the deformation densities Δ*ρ*_(1)_–Δ*ρ*_(5)_ of Zr(CO)_8_, which are associated with the most important pairwise orbital interactions Δ*E*_orb(1)_–Δ*E*_orb(5)_ given in Table 2, which nicely illustrate the charge flow that accompanies the orbital terms. The deformation densities Δ*ρ*_(1)_–Δ*ρ*_(5)_ of Ti(CO)_8_ and Hf(CO)_8_ look very similar and are shown in Figures S14 and S15 (Supporting Information). The color code of the charge migration is red→blue. Only one component of the degenerate orbital interactions is shown. The shape of Δ*ρ*_(5)_ shows that the stabilizing polarization of the (CO)_8_ cage involves a charge migration from oxygen to carbon.

**Table 2:** EDA‐NOCV results for singlet *O_h_* symmetric TM(CO)_8_ (TM=Ti, Zr, Hf) complexes at the M06/TZ2P‐ZORA//M06‐D3/def2‐TZVPP level. The interacting fragments are the metal atom TM in the singlet (S) excited state with a (*n*)s^0^(*n*)p^0^(*n*−1)d^4^ valence electronic configuration and (CO)_8_ in the singlet state. Energy values are given in kcal mol^−1^.

Energy terms	Orbital interactions	Ti (S)+(CO)_8_ (S)	Zr (S)+(CO)_8_ (S)	Hf (S)+(CO)_8_ (S)
Δ*E*_int_		−362.2	−335.8	−397.8
Δ*E*_hybrid_^[a]^		31.6	43.8	36.5
Δ*E*_Pauli_		174.4	230.4	232.7
Δ*E*_elstat_^[b]^		−184.1 (32.4 %)	−221.4 (36.3 %)	−249.0 (37.3 %)
Δ*E*_orb_^[b]^		−384.0 (67.6 %)	−388.5 (63.7 %)	−417.9 (62.7 %)
Δ*E*_orb(1)_^[c,d]^ (e_g_)	[TM(d)]→(CO)_8_ π back‐donation	−264.7 (68.9 %)	−247.3 (63.7 %)	−253.3 (60.6 %)
Δ*E*_orb(2)_^[c,d]^ (t_2g_)	[TM(d)]←(CO)_8_ σ donation	−87.5 (22.8 %)	−101.3 (26.1 %)	−110.2 (26.4 %)
Δ*E*_orb(3)_^[c]^ (a_1g_)	[TM(s)]←(CO)_8_ σ donation	−4.8 (1.3 %)	−6.5 (1.7 %)	−12.6 (3.0 %)
Δ*E*_orb(4)_^[c,d]^ (t_1u_)	[TM(p)]←(CO)_8_ σ donation	−7.3 (1.9 %)	−8.3 (2.1 %)	−12.3 (2.9 %)
Δ*E*_orb(5)_^[c]^ (a_2u_)	(CO)_8_ polarization	−3.4 (0.9 %)	−6.4 (1.6 %)	−6.1 (1.5 %)
Δ*E*_orb(rest)_		−16.3 (4.2 %)	−18.7 (4.8 %)	−23.4 (5.6 %)

[a] Metahybrid correction towards orbital interaction. [b] The values within the parentheses show the percentage contribution towards the total attractive interaction Δ*E*_elstat_+Δ*E*_orb_. [c] The values within the parentheses show the percentage contribution towards the total orbital interaction Δ*E*_orb_. [d] The sum of the two or three components is given.

**Figure 8:**
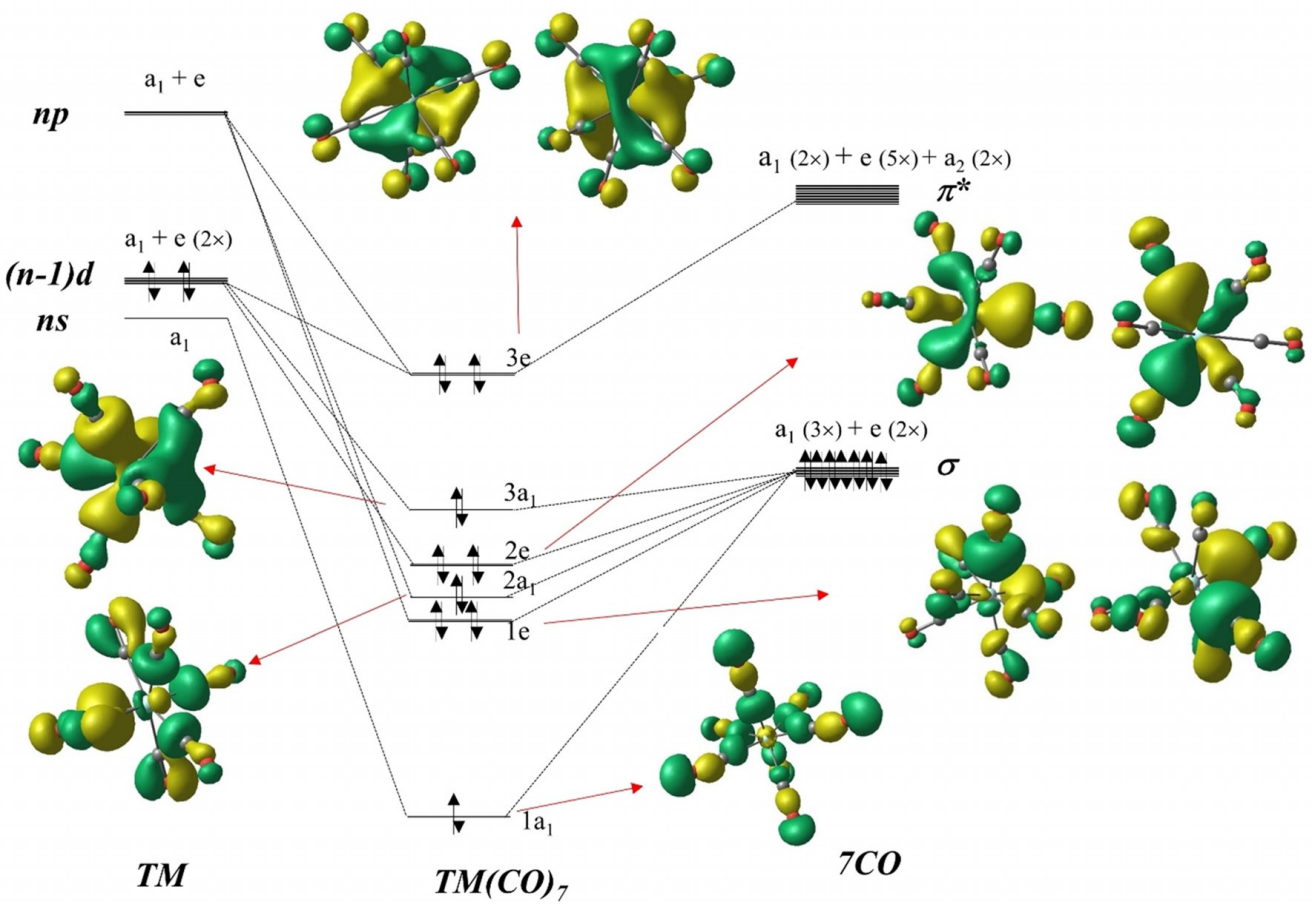
Splitting of the (*n*)s, (*n*−1)d, and (*n*)p valence orbitals of a Group 4 transition metal TM (TM=Ti, Zr, Hf) in the capped octahedron field (*C*_3*v*_) of seven CO ligands and interactions with the 5σ and 2π* valence MOs of (CO)_7_. The shape of the occupied valence MOs of Ti(CO)_7_ is also shown.

Figure 8 shows the orbital correlation diagram between the valence orbitals of a Group 4 transition metal TM in the capped octahedron (*C*_3*v*_) field of seven CO ligands in TM(CO)_7_ and the interactions with the occupied σ and vacant π* valence MOs of (CO)_7_. Considering the lower symmetry of the heptacarbonyl complex, all of the occupied σ valence MOs of (CO)_7_ can donate electronic charge to the metal, satisfying the 18‐electron rule in TM(CO)_7_. The shape of the occupied valence orbitals of Ti(CO)_7_ displayed in Figure 8 indicates that all metal AOs are involved in the occupied molecular orbitals. In principle, the metal valence (*n*)s, (*n*−1)d, and (*n*)p AOs, which split into a_1_ and e orbitals in the *C*_3*v*_ field could mix into all occupied a_1_ and e valence orbitals of the complex. Inspection of the metal AO coefficients suggest that the 1a_1_ MO of the complex has mainly contributions from the (*n*)s AO, the 2a_1_ MO has mainly contributions from the (*n*)p AO, and the 3a_1_ MO has mainly contributions from the (*n*−1)d AO. The 1e MO of the complex has mainly contributions from the (*n*)p AO, the 2e MO has mainly contributions from the (*n*−1)d AO, and the 3e MO has contributions from the (*n*−1)d and (*n*)p AOs. The latter AOs serve as polarization of the occupied (*n*−1)d AOs.

**Figure 9:**
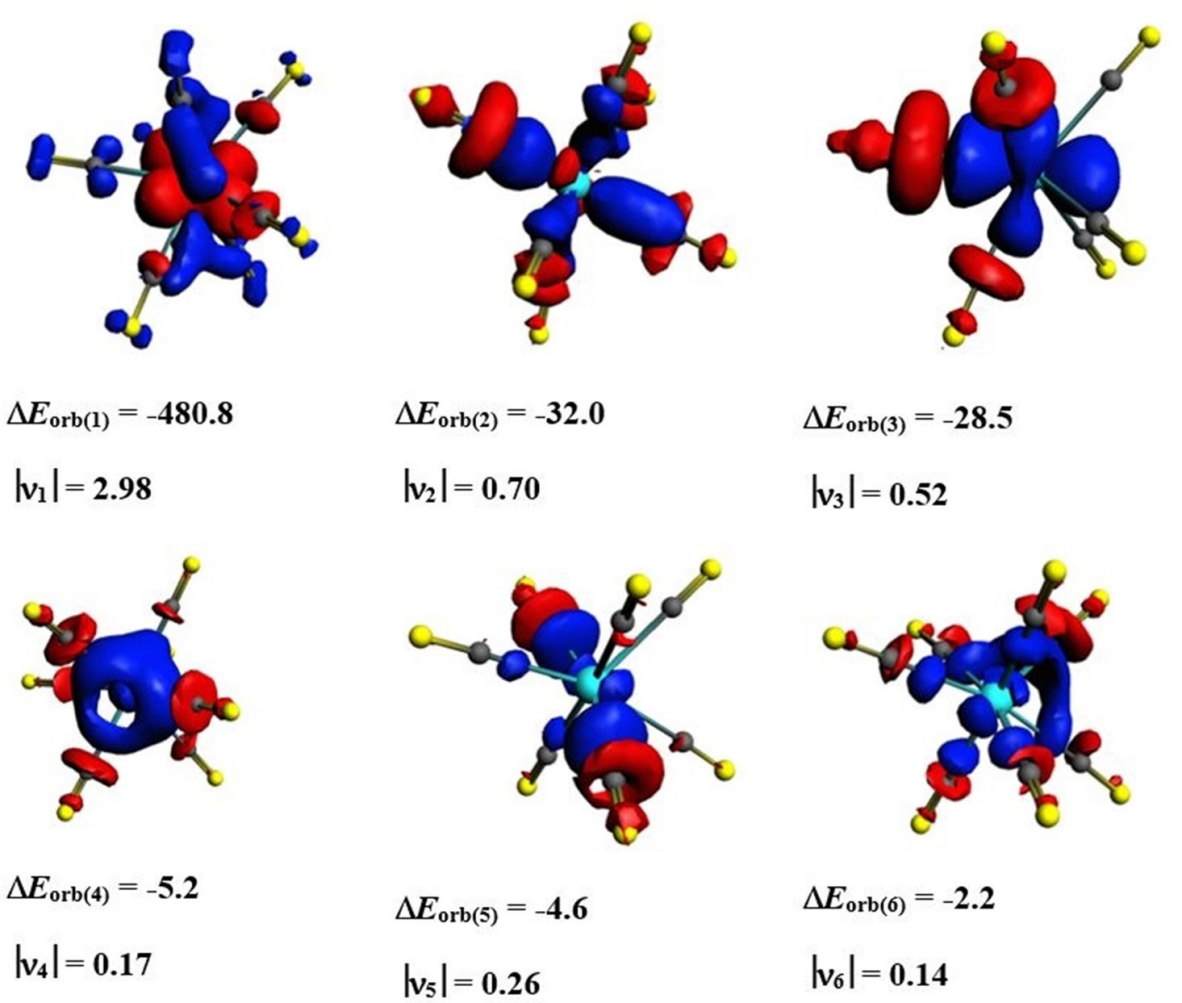
Shape of the deformation densities Δ*ρ*_(1)–(6)_, which are associated with the orbital interactions Δ*E*_orb(1)–(6)_ in Zr(CO)_7_ and eigenvalues |**ν_n_**| of the charge flow. The isosurface values are 0.004 for Δ*ρ*_(1)_, 0.001 for Δ*ρ*_(2)–(3)_, and 0.0008 for Δ*ρ*_(4)–(6)_. Only one component of the degenerate orbital interactions is shown. The color code of the charge flow is red→blue.

Table 3 shows the numerical results of the EDA‐NOCV calculations for TM(CO)_7_, which provide a quantitative account of the orbital interactions that are qualitatively sketched in Figure 8. It becomes obvious that the covalent bonding comes mainly from the [TM(d)]→(CO)_7_ π back‐donation followed by [TM(d)]←(CO)_7_ σ donation, which has two (2e and 3a_1_) components. The overall metal–ligand bonding situation in the heptacarbonyl complexes is thus very similar to those in the octacarbonyl complexes. However, why is then TM(CO)_8_ favored over TM(CO)_7_? A comparison of the EDA‐NOCV results for the two sets of complexes in Tables 2 and 3 gives a somewhat surprising answer. The intrinsic TM−(CO)_*n*_ attraction Δ*E*_int_ of the octacarbonyl complexes is (as expected) stronger than that in the heptacarbonyl complexes. However, the attractive components Δ*E*_elstat_ and Δ*E*_orb_ in TM(CO)_7_ are much stronger than that in TM(CO)_8_. The covalent bonding and the electrostatic attraction in the octacarbonyl complexes are weaker than that in the heptacarbonyl complexes, which agrees with the shorter TM−CO bonds in the latter, but the octacarbonyl complexes are yet the energetically more favored complexes. This is because the destabilizing component of the repulsive Pauli term Δ*E*_Pauli_ is much higher in TM(CO)_7_ than in TM(CO)_8_ (Tables 2 and 3). There is stronger steric repulsion between the CO ligands in the *C*_3*v*_ structures of TM(CO)_7_ as a result of the shorter TM−(CO)_7_ bonds, which overcompensates the stronger attraction compared with TM(CO)_8_. The role of Pauli repulsion is often neglected in the discussion about bond strength, which usually only considers covalent and electrostatic (ionic) attraction. It has been shown that Pauli repulsion prevents the maximum overlap of the bonding orbitals at the equilibrium bond distance^44^ and that the weaker Pauli repulsion is why CO has a higher BDE than N_2_.^45^ The deformation densities Δ*ρ*_(1)–(6)_, which are associated with the pairwise orbital interactions Δ*E*_orb(1)–(6)_ in TM(CO)_7_, are shown in Figure 9 (for Zr(CO)_7_) and in Figures S16 and S17 (Supporting Information) (for Ti(CO)_7_ and Hf(CO)_7_).

**Table 3:** EDA‐NOCV results for singlet TM(CO)_7_ (TM=Ti, Zr, Hf) complexes at the M06/TZ2P‐ZORA//M06‐D3/def2‐TZVPP level. The interacting fragments are the metal atom M in the singlet excited state with a (*n*)s^0^(*n*)p^0^(*n*−1)d^4^ valence electronic configuration and (CO)_7_ in the singlet state. Energy values are given in kcal mol^−1^.

Energy terms	Orbital interactions	Ti (S)+(CO)_7_ (S)	Zr (S)+(CO)_7_ (S)	Hf (S)+(CO)_7_ (S)
Δ*E*_int_		−352.5	−317.7	−380.4
Δ*E*_hybrid_		39.6	48.2	34.0
Δ*E*_Pauli_		582.9	580.9	630.8
Δ*E*_elstat_^[a]^		−361.9 (37.1 %)	−375.7 (39.7 %)	−432.4 (41.4 %)
Δ*E*_orb_^[a]^		−613.1 (62.9 %)	−571.0 (60.3 %)	−612.8 (58.6 %)
Δ*E*_orb(1)_^[b]^ (3e)	[TM(d)]→(CO)_7_ π back‐donation	−543.7 (88.7 %)	−480.8 (84.2 %)	−499.2 (81.5 %)
Δ*E*_orb(2)_^[b]^ (2e)	[TM(d)]←(CO)_7_ σ donation	−28.4 (4.6 %)	−32.0 (5.6 %)	−37.3 (6.1 %)
Δ*E*_orb(3)_^[b]^ (3a_1_)	[TM(d)]←(CO)_7_ σ donation	−23.1 (3.8 %)	−28.5 (5.0 %)	−31.6 (5.2 %)
Δ*E*_orb(4)_^[b]^ (1a_1_)	[TM(s)]←(CO)_7_ σ donation	−2.0 (0.3 %)	−5.2 (0.9 %)	−11.2 (1.8 %)
Δ*E*_orb(5)_^[b]^ (1e)	[TM(p)]←(CO)_7_ σ donation	−2.2 (0.4 %)	−4.6 (0.8 %)	−7.0 (1.1 %)
Δ*E*_orb(6)_^[b]^ (2a_1_)	[TM(p)]←(CO)_7_ σ donation	0.0 (0.0 %)	−2.2 (0.4 %)	−3.3 (0.5 %)
Δ*E*_orb(rest)_		−13.7 (2.2 %)	−17.7 (3.1 %)	−23.2 (3.8 %)

[a] The values in parentheses give the percentage contribution to the total attractive interactions Δ*E*_elstat_+Δ*E*_orb_. [b] The values in parentheses give the percentage contribution to the total orbital interactions Δ*E*_orb_.

The final question for the neutral complexes concerns the finding that only Zr and Hf afford stable octacarbonyl complexes whereas Ti(CO)_8_ is unstable for the loss of one CO. EDA‐NOCV calculations using TM(CO)_7_+CO as interacting fragments provide an answer. Table 4 gives the numerical results. The attractive terms Δ*E*_elstat_ and Δ*E*_orb_ in Ti(CO)_8_ are stronger than that in their heavier homologues. However, the Pauli repulsion Δ*E*_Pauli_ in the titanium octacarbonyl is significantly larger, which makes the intrinsic interaction energy Δ*E*_int_ in Ti(CO)_8_ weaker than that in the heavier octacarbonyl complexes, albeit it is still attractive. What makes Ti(CO)_8_ energetically unstable for the loss of one CO is the assembling preparation energy Δ*E*_prep_, which is clearly larger than that for Zr(CO)_8_ and Hf(CO)_8_ (Table 4). Thus, Ti(CO)_8_ is unstable for the loss of one CO because of repulsion between the CO ligands, which is stronger because of the shorter metal−CO bonds than that in the heavier homologues. The BDE values *D*_e_ in Table 4 are slightly different from the data in Figure 5, because the former values were calculated with a Slater basis set of comparable quality.

**Table 4:** EDA‐NOCV results for singlet TM(CO)_8_ (TM=Ti, Zr, Hf) complexes at the M06/TZ2P‐ZORA//M06‐D3/def2‐TZVPP level using TM(CO)_7_ and one CO as interacting fragments. Energy values are given in kcal mol^−1^.

Energy terms	Orbital interactions	CO (S)+Ti(CO)_7_ (S)	CO (S)+Zr(CO)_7_ (S)	CO (S)+Hf(CO)_7_ (S)
Δ*E*_int_		−13.7	−22.1	−24.2
Δ*E*_hybrid_		4.7	4.7	4.2
Δ*E*_Pauli_		88.6	63.0	71.4
Δ*E*_elstat_^[a]^		−54.3 (50.8 %)	−43.6 (48.6 %)	−50.2 (50.3 %)
Δ*E*_orb_^[a]^		−52.6 (49.2 %)	−46.2 (51.4 %)	−49.6 (49.7 %)
Δ*E*_orb(1)_^[b]^	[CO]→[TM(CO)_7_] σ donation	−25.2 (47.9 %)	−21.2 (45.9 %)	−23.2 (46.8 %)
Δ*E*_orb(2)_^[b]^	[CO]←[TM(CO)_7_] π back‐donation	−12.8 (24.3 %)	−12.2 (26.4 %)	−12.8 (25.8 %)
Δ*E*_orb(3)_^[b]^	[CO]←[TM(CO)_7_] π back‐donation	−12.8 (24.3 %)	−12.2 (26.4 %)	−12.8 (25.8 %)
Δ*E*_orb(rest)_		−1.8 (3.4 %)	−0.6 (1.3 %)	−0.8 (1.6 %)
Δ*E*_prep_		20.9	8.6	13.1
*D*_e_		−7.2	13.5	11.1

[a] The values in parentheses give the percentage contribution to the total attractive interactions Δ*E*_elstat_+Δ*E*_orb_. [b] The values in parentheses give the percentage contribution to the total orbital interactions Δ*E*_orb_.

The bonding analysis of the neutral complexes is presented and discussed in detail to explain the peculiar observation that Ti(CO)_7_ and TM(CO)_8_ (TM=Zr, Hf) appear as coordinatively saturated Group 4 carbonyl complexes. The EDA‐NOCV results of the cations shall only be shortly discussed. Table 5 shows the numerical results for the octacarbonyl cations TM(CO)_8_^+^ using TM^+^ and (CO)_8_ as interacting fragments. The [TM(d)]^+^→(CO)_8_ π back‐donation has two components from orbitals having a_1g_ and b_2g_ symmetry as a result of the lower *D*_4*h*_ symmetry. The contribution of the π back‐donation in the cations is (as expected) smaller than that in the neutral species (Table 2) because of the positive charge of the metal and one less d electron, but it still provides 40 %–50 % of the total orbital interactions Δ*E*_orb_. The [TM(d)]^+^←(CO)_8_ σ donation also has two components (e_g_ and b_1g_), which contribute 30 %–35 % to Δ*E*_orb_. The results for Zr(CO)_8_^+^ must be taken with some caution because the self‐consistent field (SCF) calculation of Zr^+^ did not fully converge to the (*n*)s^0^(*n*)p^0^(*n*−1)d^3^ valence electronic configuration but showed a hybrid orbital of 70 % d${}_{z{}^{2}}$ and 30 % s character. The EDA‐NOCV results for TM(CO)_6_^+^ shown in Table 6 have only one component for the four classical orbital interactions because of the octahedral (*O_h_*) symmetry of the hexacarbonyl cation. The [TM(d)]^+^→(CO)_6_ π back‐donation and [TM(d)]^+^←(CO)_6_ σ donation have about the same strength in the three systems, providing about 36 % each to the total orbital interactions Δ*E*_orb_. Thus, the most important valence orbital of the metal atoms is the (*n*−1)d AO also in the cations. The deformation densities of TM(CO)_8_^+^ and TM(CO)_6_^+^ are shown in Figures S18–S23 (Supporting Information).

**Table 5:** EDA‐NOCV results for singlet *D*_4*h*_ symmetric TM(CO)_8_^+^ (TM=Ti, Zr, Hf) complexes at the M06/TZ2P‐ZORA//M06‐D3/def2‐TZVPP level. The interacting fragments are the metal cation M^+^ in the doublet (D) excited state with a (*n*)s^0^(*n*)p^0^(*n*−1)d^3^ valence electron configuration and (CO)_8_ in the singlet state. Energy values are given in kcal mol^−1^.

Energy terms	Orbital interactions	Ti^+^ (D)+(CO)_8_ (S)	Zr^+^ (D)^[a]^+(CO)_8_ (S)	Hf^+^ (D)+(CO)_8_ (S)
Δ*E*_int_		−263.6	−278.6	−331.1
Δ*E*_hybrid_^[b]^		40.1	45.8	41.3
Δ*E*_Pauli_		181.5	236.4	183.0
Δ*E*_elstat_^[c]^		−153.9 (31.7 %)	−203.8 (36.3 %)	−199.2 (35.9 %)
Δ*E*_orb_^[c]^		−331.3 (68.3 %)	−356.9 (63.7 %)	−356.2 (64.1 %)
Δ*E*_orb(1)_^[d]^ (b_2g_)	[TM(d)]^+^→(CO)_8_ π back‐donation	−107.8 (32.5 %)	−99.2 (27.8 %)	−99.3 (27.9 %)
Δ*E*_orb(2)_^[d]^ (a_1g_)	[TM(d)]^+^→(CO)_8_ π back‐donation	−54.3 (16.4 %)	−67.6 (18.9 %)	−43.3 (12.2 %)
Δ*E*_orb(3)_^[d]^ (b_1g_)	[TM(d)]^+^←(CO)_8_ σ donation	−32.5 (9.8 %)	−37.9 (10.6 %)	−41.5 (11.7 %)
Δ*E*_orb(4)_^[d,e]^ (e_g_)	[TM(d)]^+^←(CO)_8_ σ donation	−69.4 (20.9 %)	−80.4 (22.5 %)	−84.8 (23.8 %)
Δ*E*_orb(5)_^[d]^ (a_1g_)	[TM(s)]^+^←(CO)_8_ σ donation	−9.2 (2.8 %)	−9.6 (2.7 %)	−18.0 (5.1 %)
Δ*E*_orb(6)_^[d]^ (a_2u_)	[TM(p)]^+^←(CO)_8_ σ donation	−6.8 (2.1 %)	−6.1 (1.7 %)	−7.1 (2.0 %)
Δ*E*_orb(7)_^[d,e]^ (e_u_)	[TM(p)]^+^←(CO)_8_ σ donation	−12.2 (3.7 %)	−11.2 (3.1 %)	−12.4 (3.5 %)
Δ*E*_orb(rest)_^[d]^		−39.1 (11.8 %)	−44.9 (12.6 %)	−49.8 (14.0 %)

[a] At M06/TZ2P‐ZORA level, Zr^+^ with (*n*)s^0^(*n*)p^0^(*n*−1)d^3^ valence electronic configuration was not fully converged even after several iterations. After numerous iterations, we used a state with (*n*)s^0^(*n*)p^0^(*n*−1)d^3^ configuration in which the unpaired electron is located in a hybrid orbital of 70 % d${}_{z{}^{2}}$ and 30 % s character. [b] Metahybrid correction towards orbital interaction. [c] The values within the parentheses show the percentage contribution towards the total attractive interaction Δ*E*_elstat_+Δ*E*_orb_. [d] The values within the parentheses show the percentage contribution towards the total orbital interaction Δ*E*_orb_. [e] The sum of the two components is given.

**Table 6:** EDA‐NOCV results for the octahedral (*O_h_*) complexes TM(CO)_6_^+^ (TM=Ti, Zr, Hf) in the quintet (^4^A_1g_) state at the M06/TZ2P‐ZORA//M06‐D3/def2‐TZVPP level. The interacting fragments are the metal cation M^+^ in the quartet (Q) excited state with a (*n*)s^0^(*n*)p^0^(*n*−1)d^3^ valence electronic configuration and (CO)_6_ in the singlet state. Energy values are given in kcal mol^−1^.

Energy terms	Orbital interactions	Ti^+^ (Q)+(CO)_6_ (S)	Zr^+^ (Q)+(CO)_6_ (S)	Hf^+^ (Q)+(CO)_6_ (S)
Δ*E*_int_		−193.8	−206.8	−263.0
Δ*E*_hybrid_^[a]^		35.3	34.9	32.7
Δ*E*_Pauli_		151.3	170.7	180.7
Δ*E*_elstat_^[b]^		−150.2 (39.5 %)	−170.4 (41.3 %)	−204.1 (42.8 %)
Δ*E*_orb_^[b]^		−230.2 (60.5 %)	−242.1 (58.7 %)	−272.3 (57.2 %)
Δ*E*_orb(1)_^[c,d]^ (t_2g_)	[TM(d)]^+^→(CO)_6_ π back‐donation	−84.0 (36.5 %)	−92.4 (38.2 %)	−97.3 (35.7 %)
Δ*E*_orb(2)_^[c,d]^ (e_g_)	[TM(d)]^+^←(CO)_6_ σ donation	−83.6 (36.3 %)	−87.4 (36.1 %)	−95.2 (35.0 %)
Δ*E*_orb(3)_^[c,d]^ (t_1u_)	[TM(p)]^+^←(CO)_6_ σ donation	−21.0 (9.1 %)	−18.9 (7.8 %)	−21.9 (8.0 %)
Δ*E*_orb(4)_^[c]^ (a_1g_)	[TM(s)]^+^←(CO)_6_ σ donation	−10.9 (4.7 %)	−11.6 (4.8 %)	−19.5 (7.2 %)
Δ*E*_orb(rest)_		−30.7 (13.3 %)	−31.8 (13.1 %)	−38.4 (14.1 %)

[a] Metahybrid correction towards orbital interaction. [b] The values within the parentheses show the percentage contribution towards the total attractive interaction Δ*E*_elstat_+Δ*E*_orb_. [c] The values within the parentheses show the percentage contribution towards the total orbital interaction Δ*E*_orb_. [d] The sum of the two or three components is given.

## Conclusion

We report the first experimental observation of coordinatively saturated neutral and positively charged homoleptic Group 4 metal carbonyl complexes, which have been prepared in the gas phase and/or in solid neon matrix. Combined infrared photodissociation spectroscopy and matrix isolation infrared absorption spectroscopy studies reveal that both zirconium and hafnium form eight‐coordinate carbonyl neutral and cationic complexes. The neutral octacarbonyl complexes TM(CO)_8_ have cubic (*O_h_*) geometries, whereas the cations TM(CO)_8_^+^ have *D*_4*h*_ structures. In contrast, titanium only forms the stable six‐coordinate cation complex Ti(CO)_6_^+^ and seven‐coordinate neutral complex Ti(CO)_7_. Titanium octacarbonyl Ti(CO)_8_ with *O_h_* symmetry is an energy minimum, but it is unstable with regard to loss of one CO ligand due to steric repulsion between the CO ligands. The Zr(CO)_8_ and Hf(CO)_8_ complexes represent the first experimentally observed homoleptic octacarbonyl neutral complexes of transition metals with 20 valence electrons. The molecules still fulfill the 18‐electron rule, because one doubly occupied valence orbital has a node at the metal atom, and thus, it does not mix with any of the metal valence AOs. A detailed bonding analysis by using the sophisticated EDA‐NOCV approach suggests that the major contribution of the covalent metal−CO bonding in the neutral complexes and in the cations comes from the [TM(d)]→(CO)_8_ π back‐donation. The most important valence orbitals of the metals are the d orbitals with the s and p functions playing an equally minor role. The bonding analysis shows that Zr(CO)_8_ and Hf(CO)_8_ are stable for loss of one CO because the CO ligands encounter less steric repulsion than Zr(CO)_7_ and Hf(CO)_7_. The heptacarbonyl complexes have shorter metal−CO bonds than that of the octacarbonyl complexes as a result of stronger electrostatic and covalent bonding, but the significantly smaller repulsive Pauli term makes the octacarbonyl complexes lower in energy.

The described complexes of this work appear exotic and may not attract the interest of chemists who are mainly interesting in “compounds in the bottle”. It is indeed unlikely that the Group 4 octacarbonyl complexes will find a wide application in synthesis and chemical technology. However, they increase our knowledge of the most important bonding modes of the Group 4 metal atoms and affect the 18‐electron rule, which belongs to the arsenal of elementary rules of chemistry that are fundamental to chemical research. The dication He_2_^2+^ is irrelevant for synthesis, but it is important to learn that the interference of the wave functions that give rise to covalent bonding can overcome the immense Coulomb repulsion of 200 kcal mol^−1^.^46^ Likewise it is important to know the relevance of symmetry for the electron‐counting rules in chemistry. According to the definition of coordinative saturation and unsaturation, “a complex is said to be coordinatively saturated if its electron count has attained the maximum permitted by bonding theory. For transition metals, this is normally 18e.”^47^ We show in this work that the number 18 refers only to electrons that occupy orbitals, which have the right symmetry to mix with valence orbitals of the metal. The main relevance of the present study lies in its contribution to state the 18‐electron rule more precisely.

## Conflict of interest

The authors declare no conflict of interest.

## Supporting information

As a service to our authors and readers, this journal provides supporting information supplied by the authors. Such materials are peer reviewed and may be re‐organized for online delivery, but are not copy‐edited or typeset. Technical support issues arising from supporting information (other than missing files) should be addressed to the authors.

Supplementary

## References

[1] G. N. Lewis , J. Am. Chem. Soc. 1916, 38, 762–785.

[2] I. Langmuir , Science 1921, 54, 59–67. 17843674 10.1126/science.54.1386.59

[3] The term “covalence” for the electron pair bonding model of Lewis^[1]^ was also coined for the first time by Langmuir: “… we shall denote by the term *covalence* the number of pairs of electrons that a given atom shares with its neighbors”. I. Langmuir , J. Am. Chem. Soc. 1919, 41, 868–934.

[4] For recent review articles on the historical development of the understanding of chemical bonding and bonding models, see:

[4a] G. Frenking , M. Hermann , Struct. Bonding 2016, 169, 131–156;

[4b] L. Zhao , S. Pan , N. Holzmann , P. Schwerdtfeger , G. Frenking , Chem. Rev. 2019, 119, 8781–8845; 31251603 10.1021/acs.chemrev.8b00722

[4c] L. Zhao , W. H. E. Schwarz , G. Frenking , Nat. Rev. Chem. 2019, 3, 35–47.

[5a] R. Hoffmann , J. M. Howell , E. L. Muetterties , J. Am. Chem. Soc. 1972, 94, 3047–3058;

[5b] M. Lein , G. Frenking , Aust. J. Chem. 2004, 57, 1191–1195.

[6] T. A. Albright , J. K. Burdett , M.-H. Whangbo , Orbital Interactions in Chemistry , 2nd ed., Wiley, New York, 2013.

[7a] R. E. Rundle , J. Am. Chem. Soc. 1947, 69, 1327–1331;

[7b] R. J. Hach , R. E. Rundle , J. Am. Chem. Soc. 1951, 73, 4321–4324;

[7c] R. E. Rundle , J. Am. Chem. Soc. 1963, 85, 112–113.

[8] G. C. Pimentel , J. Chem. Phys. 1951, 19, 446–448.

[9] C. A. Coulson , J. Chem. Soc. 1964, 1442–1454.

[10] G. Frenking , N. Fröhlich , Chem. Rev. 2000, 100, 717–774. 11749249 10.1021/cr980401l

[11a] M. J. S. Dewar , Bull. Soc. Chim. Fr. 1951, 18, C79;

[11b] J. Chatt , L. A. Duncanson , J. Chem. Soc. 1953, 2939–2947;

[11c] G. Frenking , J. Organomet. Chem. 2001, 635, 9–23;

[11d] Modern Coordination Chemistry: The Legacy of Joseph Chatt (Eds.: G. J. Leigh, N. Winterton), RSC, London, 2002.

[12] R. Ercoli , F. Calderazzo , A. Alberola , J. Am. Chem. Soc. 1960, 82, 2966–2967.

[13] Q. Luo , Q. S. Li , Z. H. Yu , Y. M. Xie , R. B. King , H. F. Schaefer III , J. Am. Chem. Soc. 2008, 130, 7756–7765. 18491904 10.1021/ja8003655

[14] R. Busby , W. Klotzbucher , G. A. Ozin , Inorg. Chem. 1977, 16, 822–828.

[15a] M. F. Zhou , L. Andrews , J. Phys. Chem. A 1999, 103, 5259–5268;

[15b] M. F. Zhou , L. Andrews , J. Am. Chem. Soc. 2000, 122, 1531–1539.

[16] F. Meyer , P. B. Armentrout , Mol. Phys. 1996, 88, 187–197.

[17a] A. D. Brathwaite , M. A. Duncan , J. Phys. Chem. A 2013, 117, 11695–11703; 23485174 10.1021/jp400793h

[17b] X. J. Zhou , J. M. Cui , Z. H. Li , G. J. Wang , Z. P. Liu , M. F. Zhou , J. Phys. Chem. A 2013, 117, 1514–1521. 23330878 10.1021/jp3120429

[18a] A. D. Brathwaite , J. A. Maner , M. A. Duncan , Inorg. Chem. 2014, 53, 1166–1169; 24380416 10.1021/ic402729g

[18b] H. Xie , J. Wang , Z. B. Qin , L. Shi , Z. C. Tang , X. P. Xing , J. Phys. Chem. A 2014, 118, 9380–9385; 25203282 10.1021/jp504079k

[18c] A. M. Ricks , Z. D. Reed , M. A. Duncan , J. Am. Chem. Soc. 2009, 131, 9176–9177; 19522497 10.1021/ja903983u

[18d] A. M. Ricks , A. D. Brathwaite , M. A. Duncan , J. Phys. Chem. A 2013, 117, 1001–1010. 22486750 10.1021/jp301679m

[19a] J. W. Dicke , N. J. Stibrich , H. F. Schaefer , Chem. Phys. Lett. 2008, 456, 13–18;

[19b] M. R. Sievers , P. B. Armentrout , J. Phys. Chem. 1995, 99, 8135–8141.

[20] J. Bohnenberger , W. Feuerstein , D. Himmel , M. Daub , F. Breher , I. Krossing , Nat. Commun. 2019, 10, 624. 30733449 10.1038/s41467-019-08517-2 PMC6367395

[21] J. Jin , T. Yang , K. Xin , G. Wang , X. Wang , M. Zhou , G. Frenking , Angew. Chem. Int. Ed. 2018, 57, 6236; 10.1002/anie.201802590 29578636

[22] X. Wu , L. Zhao , J. Jin , S. Pan , W. Li , X. Jin , G. Wang , M. Zhou , G. Frenking , Science 2018, 361, 912–916. 30166489 10.1126/science.aau0839

[23] Q. Wang , S. Pan , S. Lei , J. Jin , G. Deng , G. Wang , L. Zhao , M. Zhou , G. Frenking , Nat. Commun. 2019, 10, 3375. 31358748 10.1038/s41467-019-11323-5 PMC6662891

[24a] G. J. Wang , C. X. Chi , X. P. Xing , C. F. Ding , M. F. Zhou , Sci. China Chem. 2014, 57, 172–177;

[24b] G. J. Wang , C. X. Chi , J. M. Cui , X. P. Xing , M. F. Zhou , J. Phys. Chem. A 2012, 116, 2484–2489. 22360767 10.1021/jp211936b

[25] G. J. Wang , M. F. Zhou , Int. Rev. Phys. Chem. 2008, 27, 1–25.

[26] Y. Zhao , D. G. Truhlar , Theor. Chem. Acc. 2008, 120, 215–241.

[27a] F. Weigend , R. Ahlrichs , Phys. Chem. Chem. Phys. 2005, 7, 3297–3305; 16240044 10.1039/b508541a

[27b] F. Weigend , Phys. Chem. Chem. Phys. 2006, 8, 1057–1065. 16633586 10.1039/b515623h

[28] S. Grimme , J. Antony , S. Ehrlich , H. Krieg , J. Chem. Phys. 2010, 132, 154104. 20423165 10.1063/1.3382344

[29] Gaussian 16, Revision A.03, M. J. Frisch, G. W. Trucks, H. B. Schlegel, G. E. Scuseria, M. A. Robb, J. R. Cheeseman, G. Scalmani, V. Barone, G. A. Petersson, H. Nakatsuji, X. Li, M. Caricato, A. V. Marenich, J. Bloino, B. G. Janesko, R. Gomperts, B. Mennucci, H. P. Hratchian, J. V. Ortiz, A. F. Izmaylov, J. L. Sonnenberg, D. Williams-Young, F. Ding, F. Lipparini, F. Egidi, J. Goings, B. Peng, A. Petrone, T. Henderson, D. Ranasinghe, V. G. Zakrzewski, J. Gao, N. Rega, G. Zheng, W. Liang, M. Hada, M. Ehara, K. Toyota, R. Fukuda, J. Hasegawa, M. Ishida, T. Nakajima, Y. Honda, O. Kitao, H. Nakai, T. Vreven, K. Throssell, J. A. Montgomery, Jr., J. E. Peralta, F. Ogliaro, M. J. Bearpark, J. J. Heyd, E. N. Brothers, K. N. Kudin, V. N. Staroverov, T. A. Keith, R. Kobayashi, J. Normand, K. Raghavachari, A. P. Rendell, J. C. Burant, S. S. Iyengar, J. Tomasi, M. Cossi, J. M. Millam, M. Klene, C. Adamo, R. Cammi, J. W. Ochterski, R. L. Martin, K. Morokuma, O. Farkas, J. B. Foresman, D. J. Fox, Gaussian, Inc., Wallingford CT, **2016**.

[30] T. Ziegler , A. Rauk , Theor. Chim. Acta 1977, 46, 1–10.

[31a] M. Mitoraj , A. Michalak , Organometallics 2007, 26, 6576–6580;

[31b] M. Mitoraj , A. Michalak , J. Mol. Model. 2008, 14, 681–687. 18278526 10.1007/s00894-008-0276-1

[32a] ADF2017, SCM, Theoretical Chemistry, Vrije Universiteit, Amsterdam, The Netherlands, http://www.scm.com;

[32b] G. te Velde , F. M. Bickelhaupt , E. J. Baerends , C. F. Guerra , S. J. A. Van Gisbergen , J. G. Snijders , T. Ziegler , J. Comput. Chem. 2001, 22, 931–967.

[33a] A. Michalak , M. Mitoraj , T. Ziegler , J. Phys. Chem. A 2008, 112, 1933–1939; 18266342 10.1021/jp075460u

[33b] M. P. Mitoraj , A. Michalak , T. Ziegler , J. Chem. Theory Comput. 2009, 5, 962–975. 26609605 10.1021/ct800503d

[34] E. van Lenthe , E. J. Baerends , J. Comput. Chem. 2003, 24, 1142–1156. 12759913 10.1002/jcc.10255

[35] E. van Lenthe , A. Ehlers , E. J. Baerends , J. Chem. Phys. 1999, 110, 8943.

[36a] L. Zhao , M. von Hopffgarten , D. M. Andrada , G. Frenking , WIREs Comput. Mol. Sci. 2018, 8, e1345;

[36b] G. Frenking , F. M. Bickelhaupt , The Chemical Bond: Fundamental Aspects of Chemical Bonding (Eds.: G. Frenking, S. Shaik), Wiley-VCH, Weinheim, 2014, pp. 121–158;

[36c] G. Frenking , R. Tonner , S. Klein , N. Takagi , T. Shimizu , A. Krapp , K. K. Pandey , P. Parameswaran , Chem. Soc. Rev. 2014, 43, 5106–5139; 24916774 10.1039/c4cs00073k

[36d] L. Zhao , M. Hermann , N. Holzmann , G. Frenking , Coord. Chem. Rev. 2017, 344, 163–204;

[36e] G. Frenking , M. Hermann , D. M. Andrada , N. Holzmann , Chem. Soc. Rev. 2016, 45, 1129–1144. 26815221 10.1039/c5cs00815h

[37a] J. Wang , G. T. Long , E. Weitz , J. Phys. Chem. A 2001, 105, 3765–3772;

[37b] J. N. Harvey , M. Aschi , Faraday Discuss. 2003, 124, 129–143; 14527214 10.1039/b211871h

[37c] J. N. Harvey , Phys. Chem. Chem. Phys. 2007, 9, 331–343. 17199148 10.1039/b614390c

[38] H. W. Kroto , J. R. Heath , S. C. O'Brien , R. F. Curl , R. E. Smalley , Nature 1985, 318, 162–163.

[39] M. A. Duncan , Rev. Sci. Instrum. 2012, 83, 041101. 22559508 10.1063/1.3697599

[40] M. E. Jacox , Chem. Soc. Rev. 2002, 31, 108–115. 12109204 10.1039/b102907j

[41a] C. Chi , S. Pan , J. Jin , L. Meng , M. Luo , L. Zhao , M. Zhou , G. Frenking , Chem. Eur. J. 2019, 25, 11772–11784; 31276242 10.1002/chem.201902625 PMC6772027

[41b] J. Jin , S. Pan , X. Jin , S. Lei , L. Zhao , G. Frenking , M. Zhou , Chem. Eur. J. 2019, 25, 3229–3234; 30566753 10.1002/chem.201805260

[41c] C. Poggel , G. Frenking , Chem. Eur. J. 2018, 24, 11675–11782; 29718560 10.1002/chem.201801410

[41d] S. Pan , L. Zhao , H. V. R. Dias , G. Frenking , Inorg. Chem. 2018, 57, 7780–7791; 29900734 10.1021/acs.inorgchem.8b00851

[41e] X. Wu , L. Zhao , D. Jiang , I. Fernández , R. Berger , M. Zhou , G. Frenking , Angew. Chem. Int. Ed. 2018, 57, 3974–3980; 10.1002/anie.201713002 29431895

[41f] M. Chen , Q. Zhang , M. Zhou , D. M. Andrada , G. Frenking , Angew. Chem. Int. Ed. 2015, 54, 124–128; 10.1002/anie.201406264 25369759

[42] E. P. A. Couzijn , Y.-Y. Lai , A. Limacher , P. Chen , Organometallics 2017, 36, 3205–3214.

[43] Recent review: L. Zhao , M. Hermann , W. H. E. Schwarz , G. Frenking , Nat. Rev. Chem. 2019, 3, 48–63.

[44] A. Krapp , F. M. Bickelhaupt , G. Frenking , Chem. Eur. J. 2006, 12, 9196–9216. 17024702 10.1002/chem.200600564

[45] C. Esterhuysen , G. Frenking , Theor. Chem. Acc. 2004, 111, 381–389. Erratum: **2005**, *113*, 294.

[46] M. Guilhaus , A. G. Brenton , J. H. Beynon , M. Rabenovic , P. von R. Schleyer , J. Phys. B 1984, 17, L605.

[47a] Encyclopedia of Inorganic Chemistry, 2nd ed. (Ed.: R. B. King), Wiley, New York, 2006;

[47b] P. Muller , Pure Appl. Chem. 1994, 66, 1077.

