# Supplementary material for: Filling a Gap: The Coordinatively Saturated Group 4 Carbonyl Complexes TM(CO)8 (TM=Zr, Hf) and Ti(CO)7
Source: Chemistry. 2020 Jul 20;26(46):10487–500. doi: 10.1002/chem.201905552 (PMC7496348; doi:10.1002/chem.201905552)
Supplement: Supplementary file 1 — Supplementary [file CHEM-26-10487-s001.pdf]

Chemistry–A European Journal

Supporting Information

Filling a Gap: The Coordinatively Saturated Group 4 Carbonyl Complexes $TM(CO)_8$ (TM = Zr, Hf) and $Ti(CO)_7$

Guohai Deng+,[a] Shujun Lei+,[a] Sudip Pan+,[b, c] Jiaye Jin,[a] Guanjun Wang,[a] Lili Zhao,[b] Mingfei Zhou,*[a] and Gernot Frenking*[b, c]

Supporting Information for

Filling a Gap: The Coordinatively Saturated Group 4 Carbonyl Complexes $TM(CO)_8$ (TM = Zr, Hf) and $Ti(CO)_7$

Figures S1 – S23
Tables S1 – S2

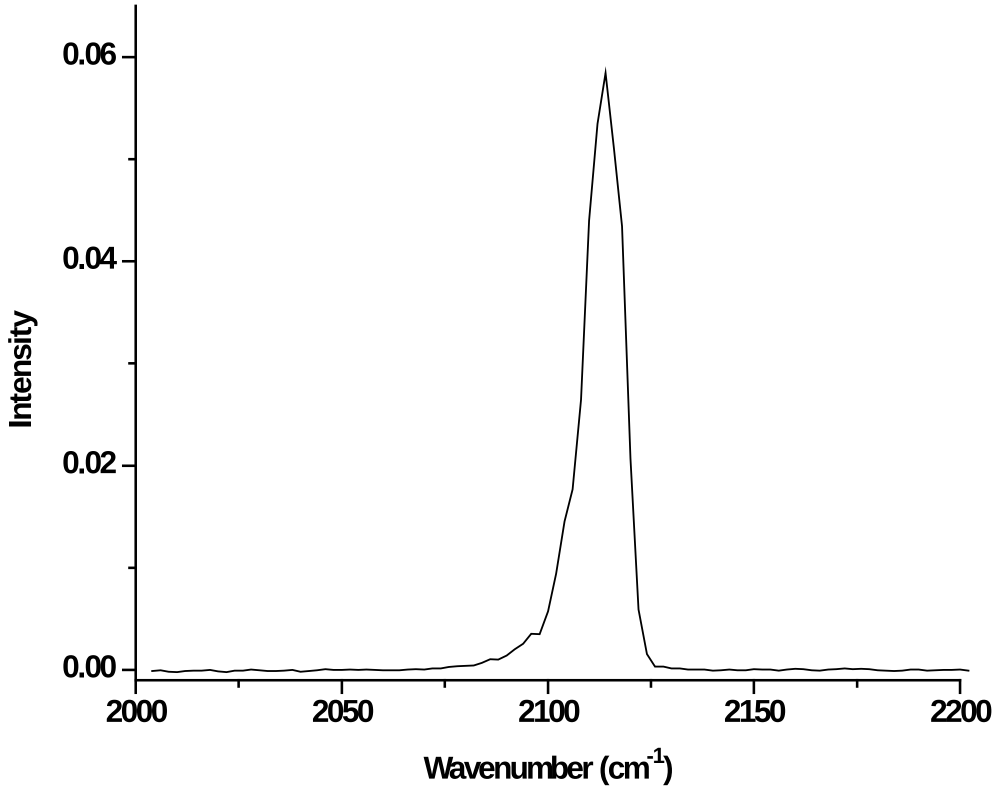

Figure S1. Infrared photodissociation spectrum of the $Ti(CO)_6^+$ complex in the carbonyl stretching frequency region.

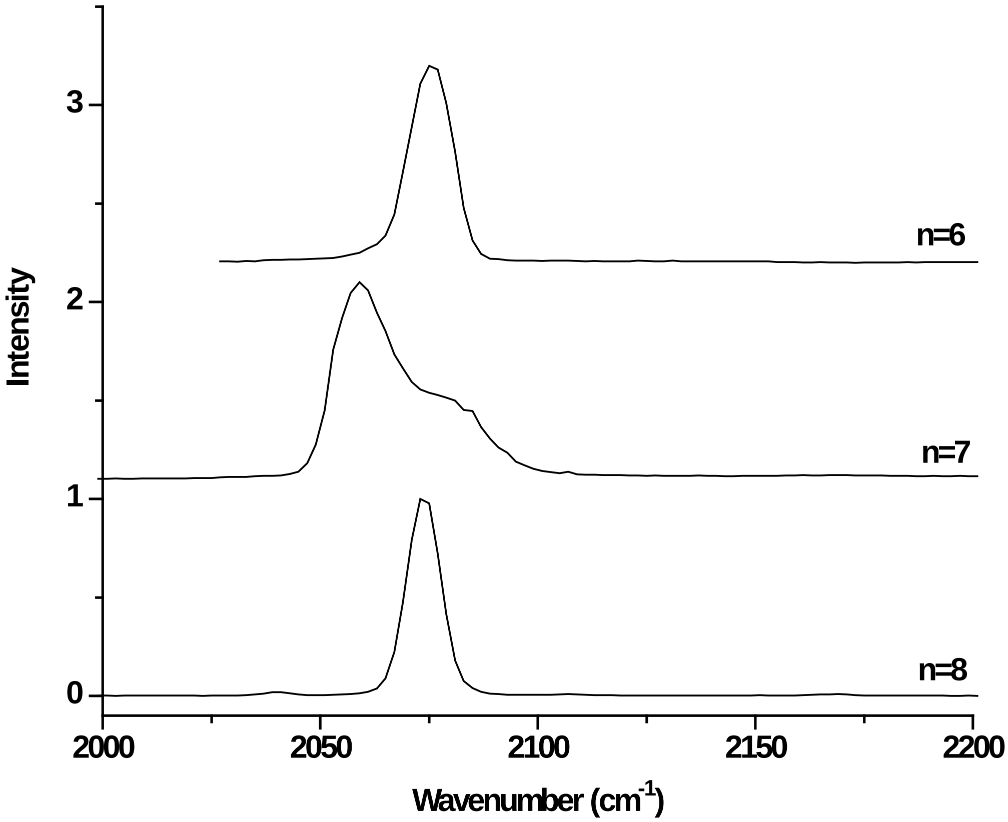

Figure S2. Infrared photodissociation spectra of the $Hf(CO)_n^+$ (n = 6-8) complexes in the carbonyl stretching frequency region.

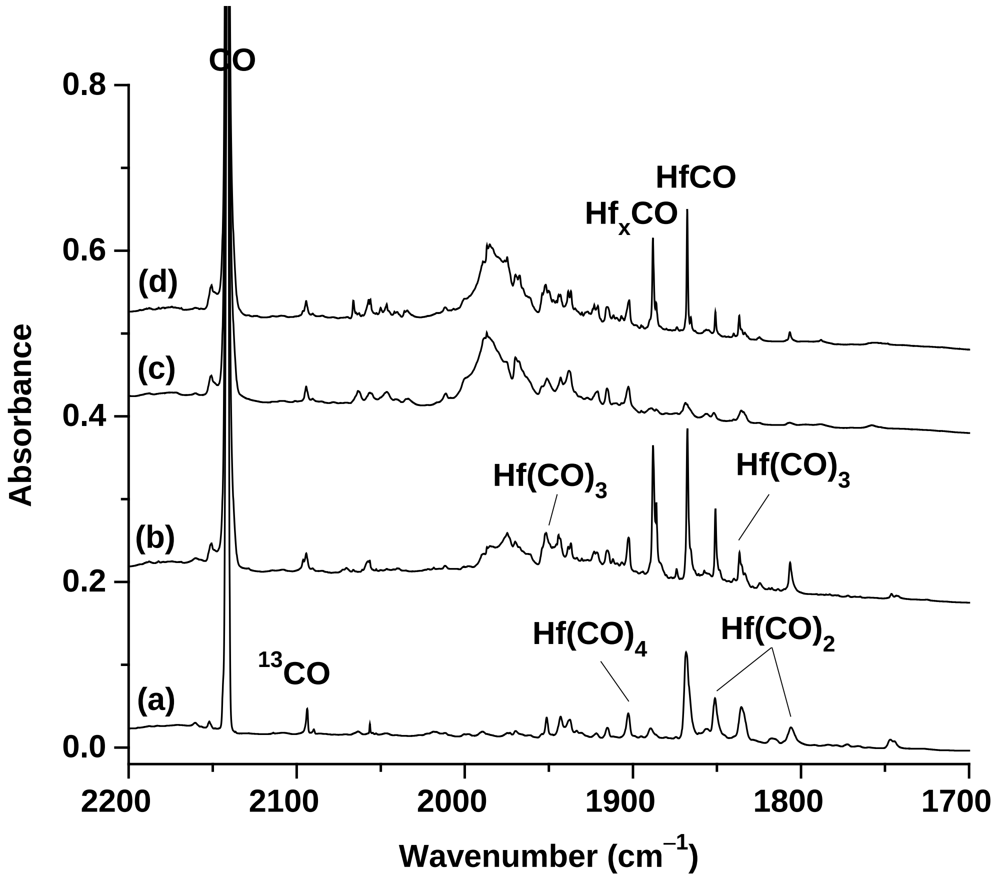

Figure S3. Infrared spectra in the 2200–1700 cm^{-1} region from co-deposition of laser evaporated hafnium atoms with 0.2 % CO in neon; (a) after 30 min of sample deposition at 4 K, (b) after annealing to 12 K, (c) after 15 min of UV-visible light irradiation, and (d) after another annealing to 12 K.

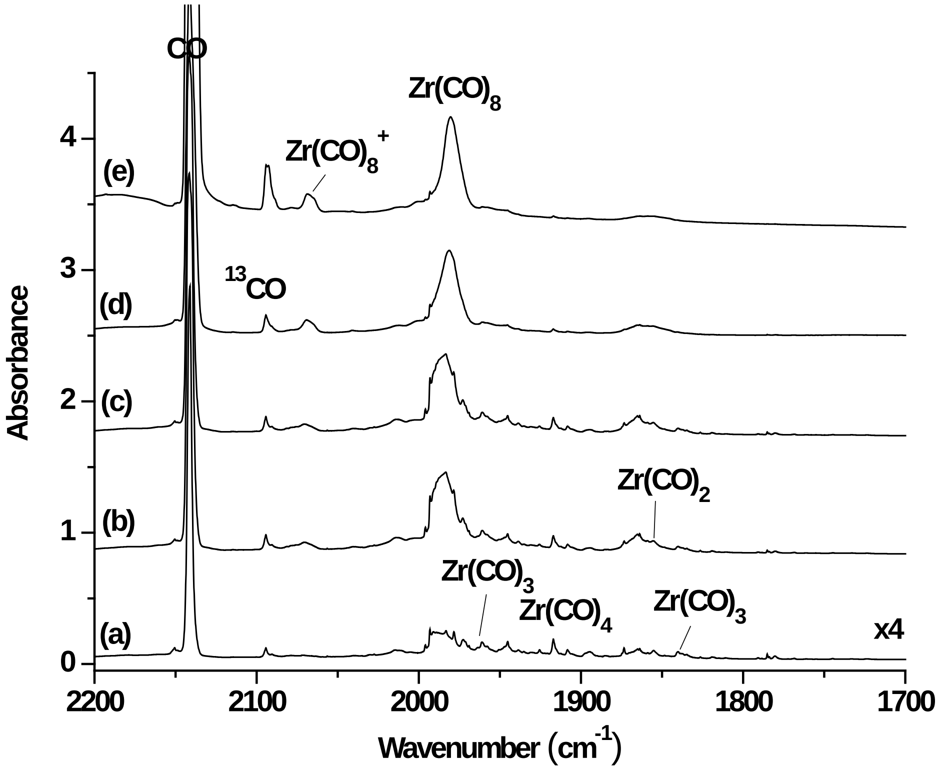

Figure S4. Infrared spectra in the 2200–1700 cm^{-1} region from co-deposition of laser evaporated zirconium atoms with different concentrations of CO in neon (The spectra were taken after 12 K annealing). (a) 0.2%, (b) 0.5 %, (c) 1%, (d) 2.0 %, and (e) 5.0 %.

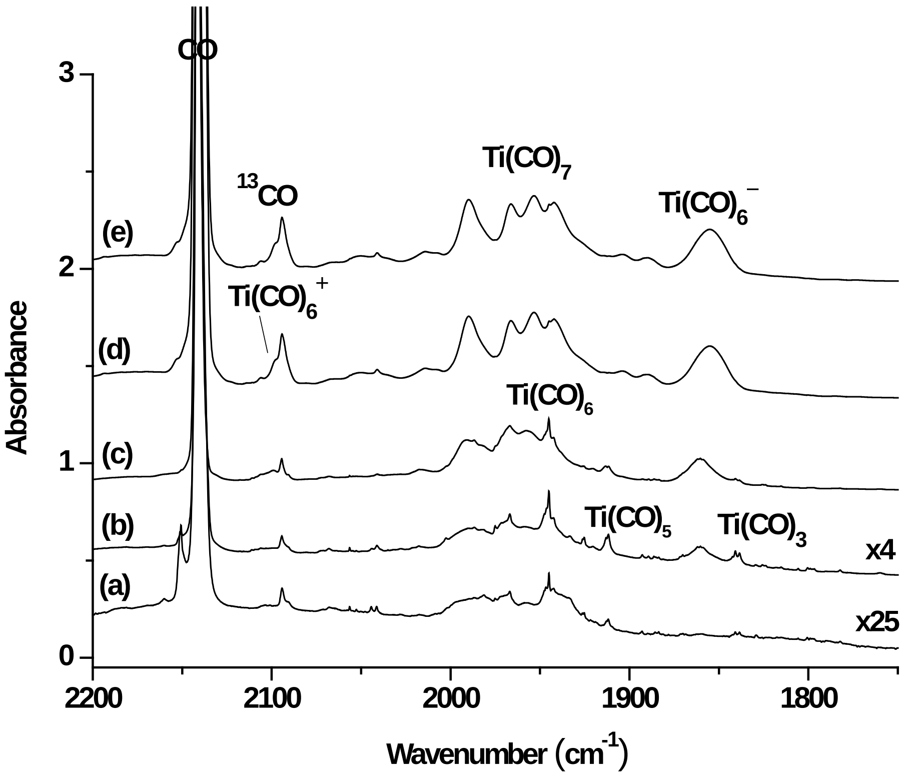

Figure S5. Infrared spectra in the 2200–1750 cm^{-1} region from co-deposition of laser evaporated titanium atoms with different concentrations of CO in neon (The spectra were taken after 12 K annealing). (a) 0.2%, (b) 0.5 %, (c) 1%, (d) 2.0 %, and (e) 5.0 %.

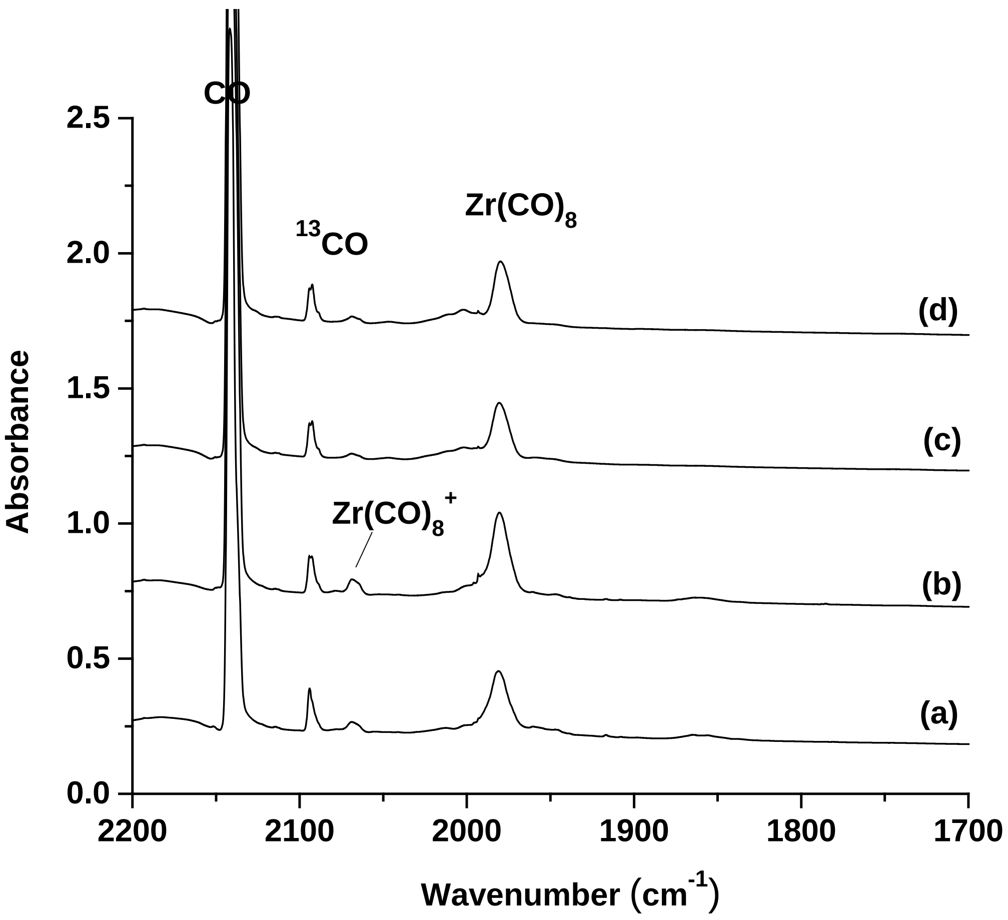

Figure S6. Infrared spectra in the 2200–1700 cm^{-1} region from co-deposition of laser evaporated zirconium atoms with 2.5 % CO in neon; (a) after 30 min of sample deposition at 4 K, (b) after annealing to 12 K, (c) after 15 min of UV-visible light irradiation, and (d) after another annealing to 12 K.

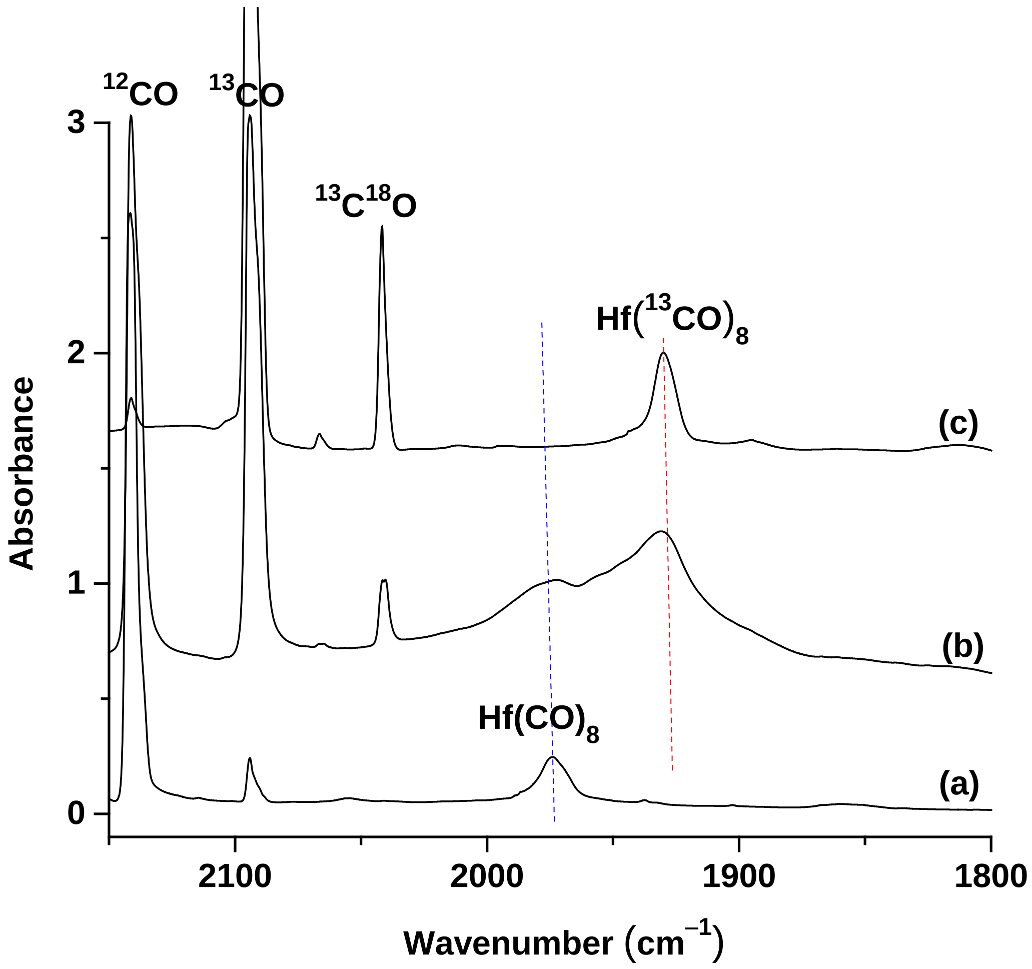

Figure S7. Infrared spectra in the 2150–1800 cm^{-1} region from co-deposition of laser evaporated hafnium atoms with isotopic-substituted CO samples in neon. a) 2.5 % ^{12}CO, (b) 1.5 % ^{12}CO + 3.0 % ^{13}CO, and (c) 2.5 % ^{13}CO.

<table>
<tr>
<td>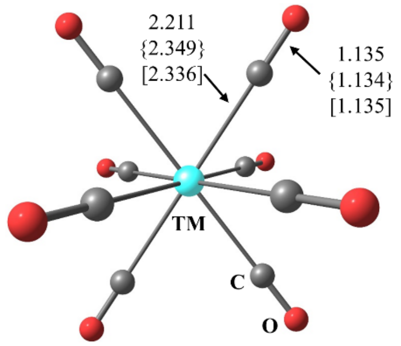

9.3 {0.0}[0.0]
$TM(CO)_8$ (O_h, $^1A_{1g}$); TM = Ti {Zr}[Hf]</td>
<td>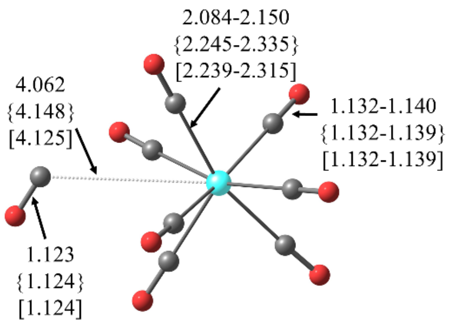

0.0 {11.4}[11.3]
$TM(CO)_7\cdots CO$ (C_s, $^1A'$)</td>
<td>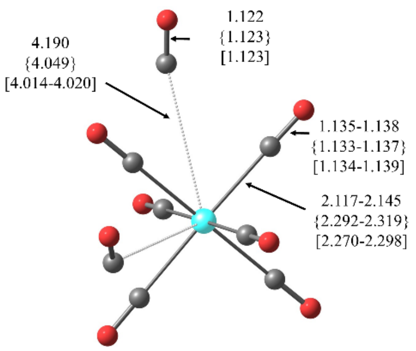

15.4 {34.3}[35.2]
$TM(CO)_6\cdots 2CO$ (Ti: C_{2v}, $^3B_2$; Zr: C_s, $^3A''$; Hf: C_1, ^3A)</td>
</tr>
<tr>
<td>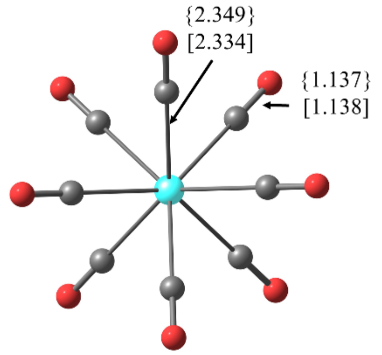

{38.6}[36.3]
$TM(CO)_8$ (D_{4d}, $^3A_1$)</td>
<td></td>
<td></td>
</tr>
</table>

Figure S8. Different minimum energy isomers of $TM(CO)_8$ (TM = Ti, {Zr}, [Hf]) complex at the M06-D3/def2-TZVPP level. Bond distances are in Å and relative energies are in kcal/mol.

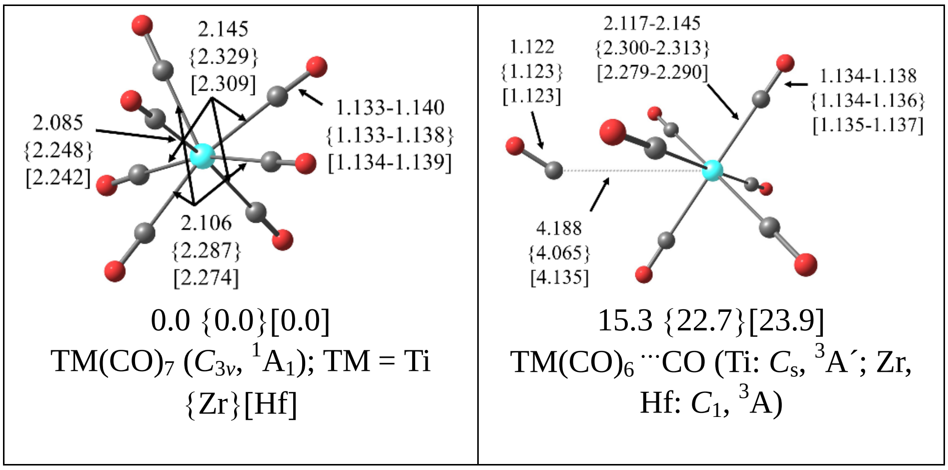

Figure S9. Different minimum energy isomers of $TM(CO)_7$ (TM = Ti, {Zr}, [Hf]) complex at the M06-D3/def2-TZVPP level. Bond distances are in Å and relative energies are in kcal/mol.

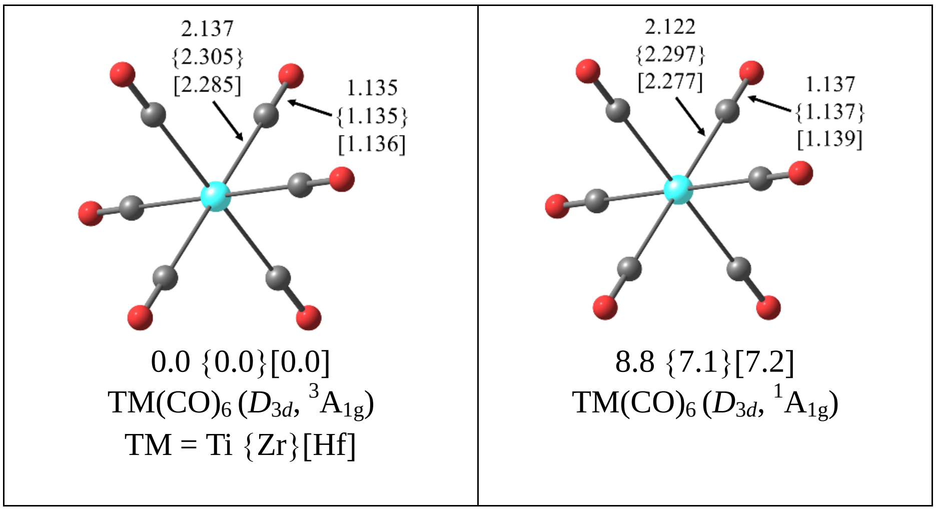

Figure S10. Different minimum energy isomers of $TM(CO)_6$ (TM = Ti, {Zr}, [Hf]) complex at the M06-D3/def2-TZVPP level. Bond distances are in Å and relative energies are in kcal/mol.

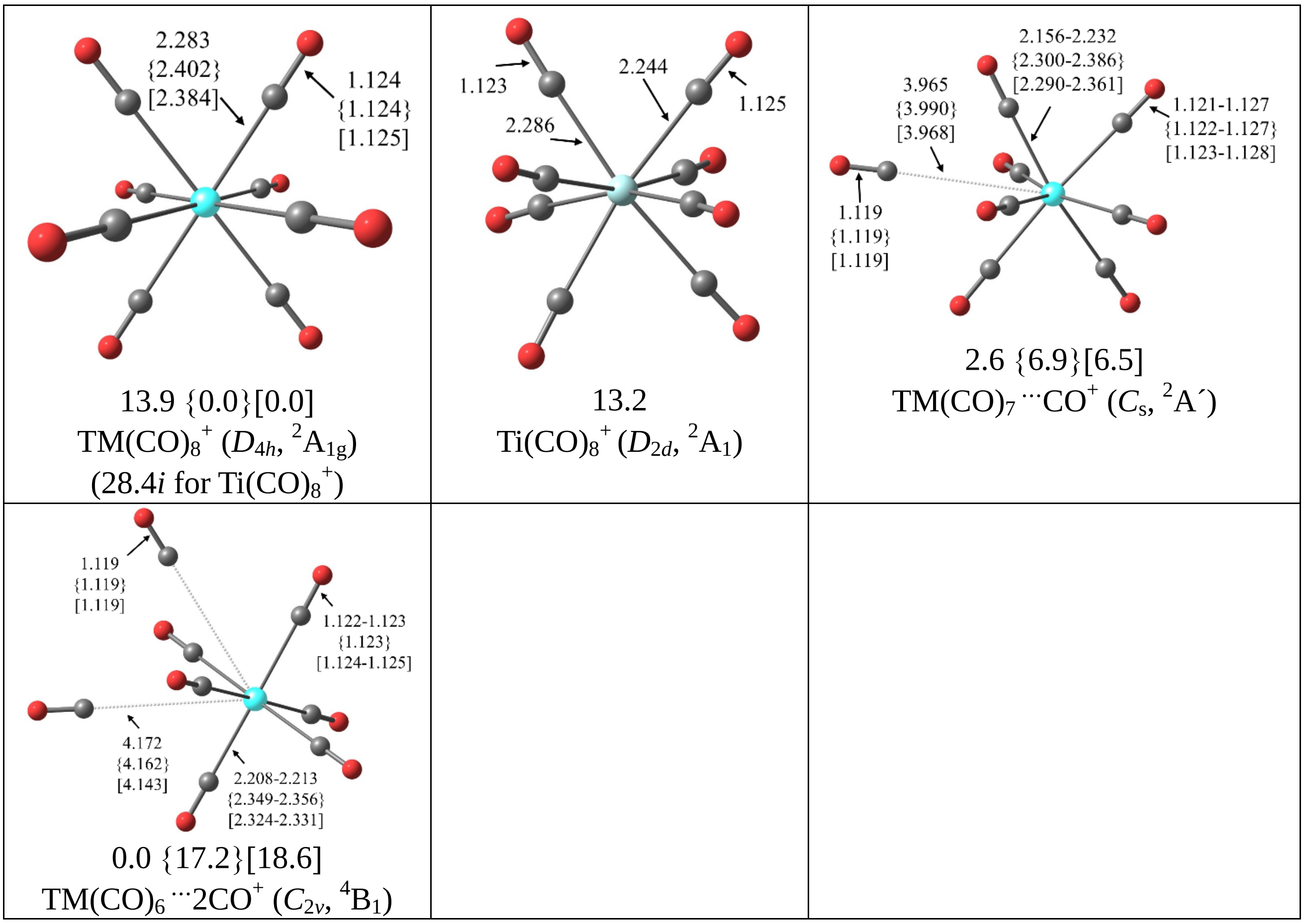

Figure S11. Different minimum energy isomers of TM(CO)$_8^+$ (TM = Ti {Zr}, [Hf]) complex at the M06-D3/def2-TZVPP level. Bond distances are in Å and relative energies are in kcal/mol.

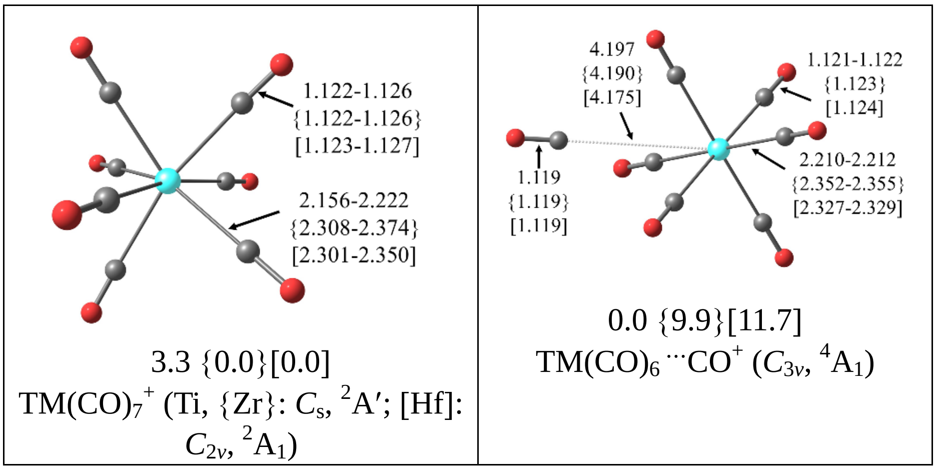

Figure S12. Different minimum energy isomers of $TM(CO)_7^+$ (TM = Ti, {Zr}, [Hf]) complex at the M06-D3/def2-TZVPP level. Bond distances are in Å and relative energies are in kcal/mol.

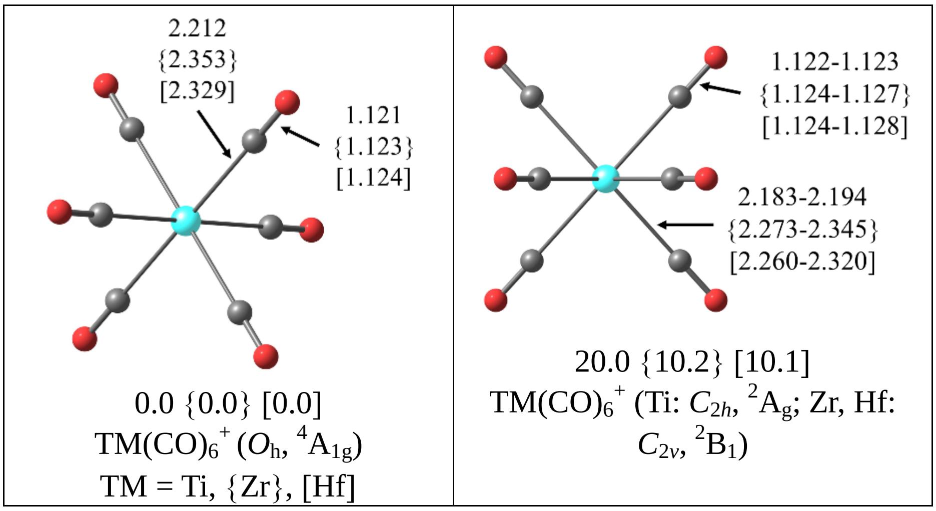

Figure S13. Different minimum energy isomers of $TM(CO)_6^+$ (TM = Ti, {Zr}, [Hf]) complex at the M06-D3/def2-TZVPP level. Bond distances are in Å and relative energies are in kcal/mol.

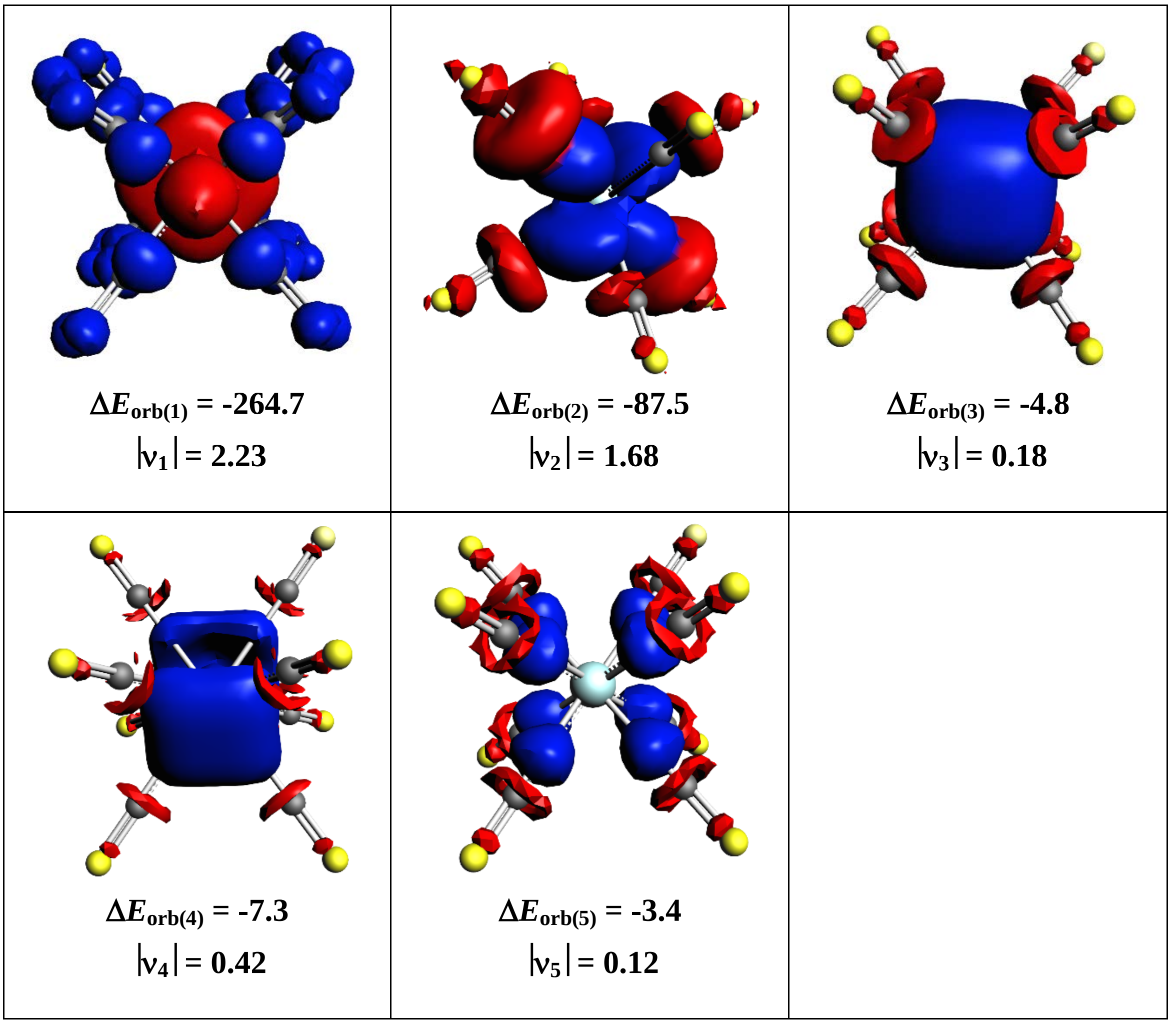

Figure S14. Shape of the deformation densities $\Delta\rho_{(1)\text{-}(5)}$, which are associated with the orbital interactions $\Delta E_{orb(1)\text{-}(5)}$ in $Ti(CO)_8$ and eigenvalues $|\nu_n|$ of the charge flow. The isosurface values are 0.002 for $\Delta\rho_{(1)\text{-}(2)}$ and 0.0008 for $\Delta\rho_{(3)\text{-}(5)}$. Only one component of the degenerate orbital interactions is shown. The color code of the charge flow is red → blue.

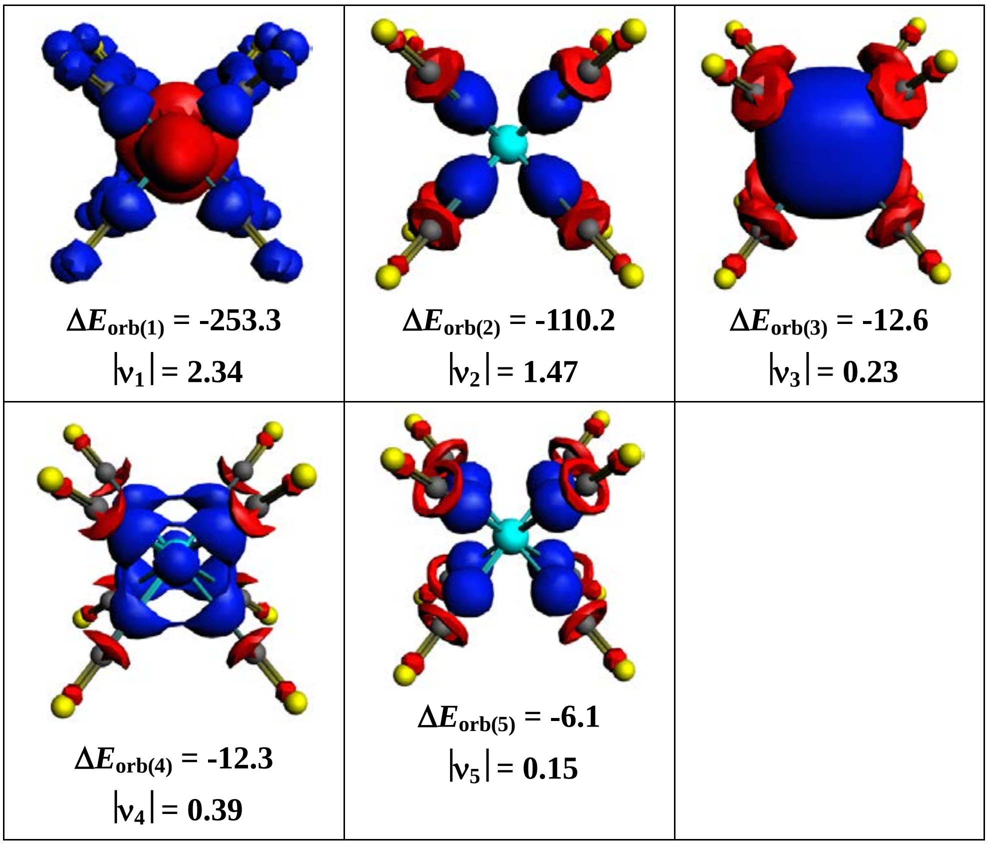

Figure S15. Shape of the deformation densities $\Delta\rho_{(1)\text{-}(5)}$, which are associated with the orbital interactions $\Delta E_{orb(1)\text{-}(5)}$ in $Hf(CO)_8$ and eigenvalues $|\nu_n|$ of the charge flow. The isosurface values are 0.002 for $\Delta\rho_{(1)\text{-}(2)}$ and 0.0008 for $\Delta\rho_{(3)\text{-}(5)}$. Only one component of the degenerate orbital interactions is shown. The color code of the charge flow is red → blue.

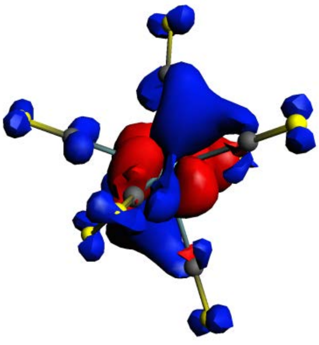 $\Delta E_{\mathbf{orb(1)}}$ = -543.7 $\vert\nu_1\vert$ = 3.10	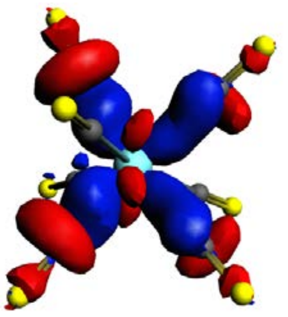 $\Delta E_{\mathbf{orb(2)}}$ = -28.4 $\vert\nu_2\vert$ = 0.72	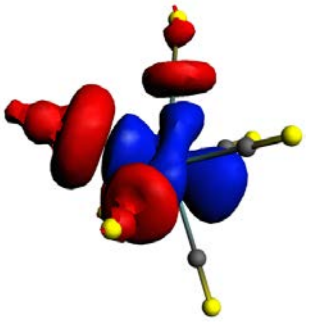 $\Delta E_{\mathbf{orb(3)}}$ = -23.1 $\vert\nu_3\vert$ = 0.61
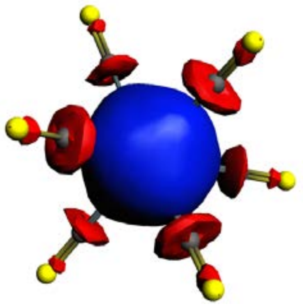 $\Delta E_{\mathbf{orb(4)}}$ = -2.0 $\vert\nu_4\vert$ = 0.17	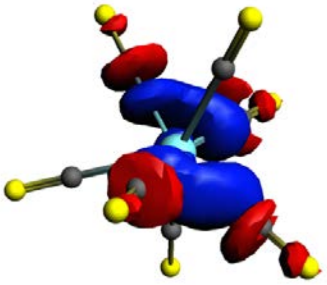 $\Delta E_{\mathbf{orb(5)}}$ = -2.2 $\vert\nu_5\vert$ = 0.28	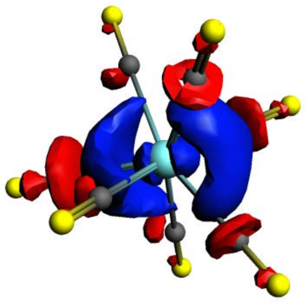 $\Delta E_{\mathbf{orb(6)}}$ = 0.0 $\vert\nu_6\vert$ = 0.15

Figure S16. Shape of the deformation densities $\Delta\rho_{(1)\text{-}(6)}$, which are associated with the orbital interactions $\Delta E_{orb(1)\text{-}(6)}$ in $Ti(CO)_7$ and eigenvalues $|\nu_n|$ of the charge flow. The isosurface values are 0.004 for $\Delta\rho_{(1)}$, 0.001 for $\Delta\rho_{(2)\text{-}(3)}$, and 0.0008 for others. Only one component of the degenerate orbital interactions is shown. The color code of the charge flow is red → blue.

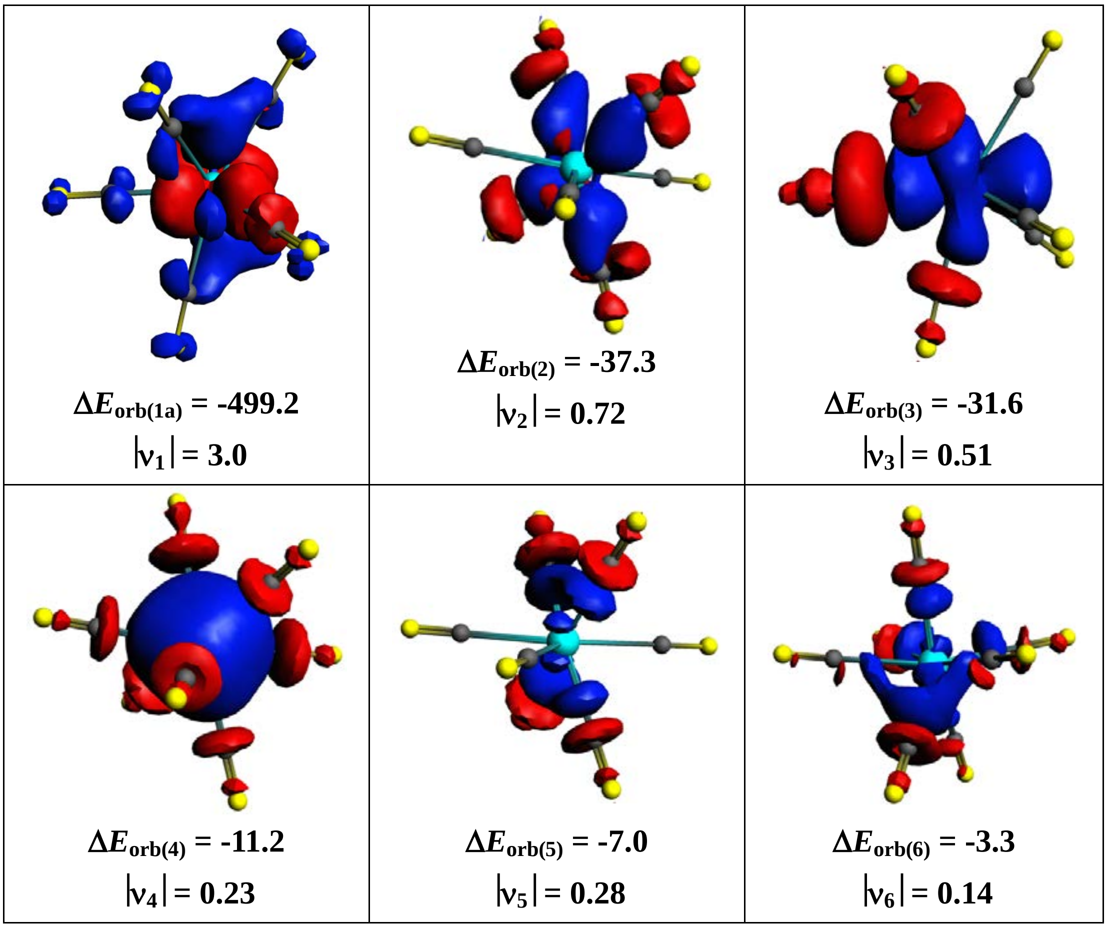

Figure S17. Shape of the deformation densities $\Delta\rho_{(1)\text{-}(6)}$, which are associated with the orbital interactions $\Delta E_{orb(1)\text{-}(6)}$ in $Hf(CO)_7$ and eigenvalues $|\nu_n|$ of the charge flow. The isosurface values are 0.004 for $\Delta\rho_{(1)}$, 0.001 for $\Delta\rho_{(2)\text{-}(3)}$, and 0.0008 for others. Only one component of the degenerate orbital interactions is shown. The color code of the charge flow is red → blue.

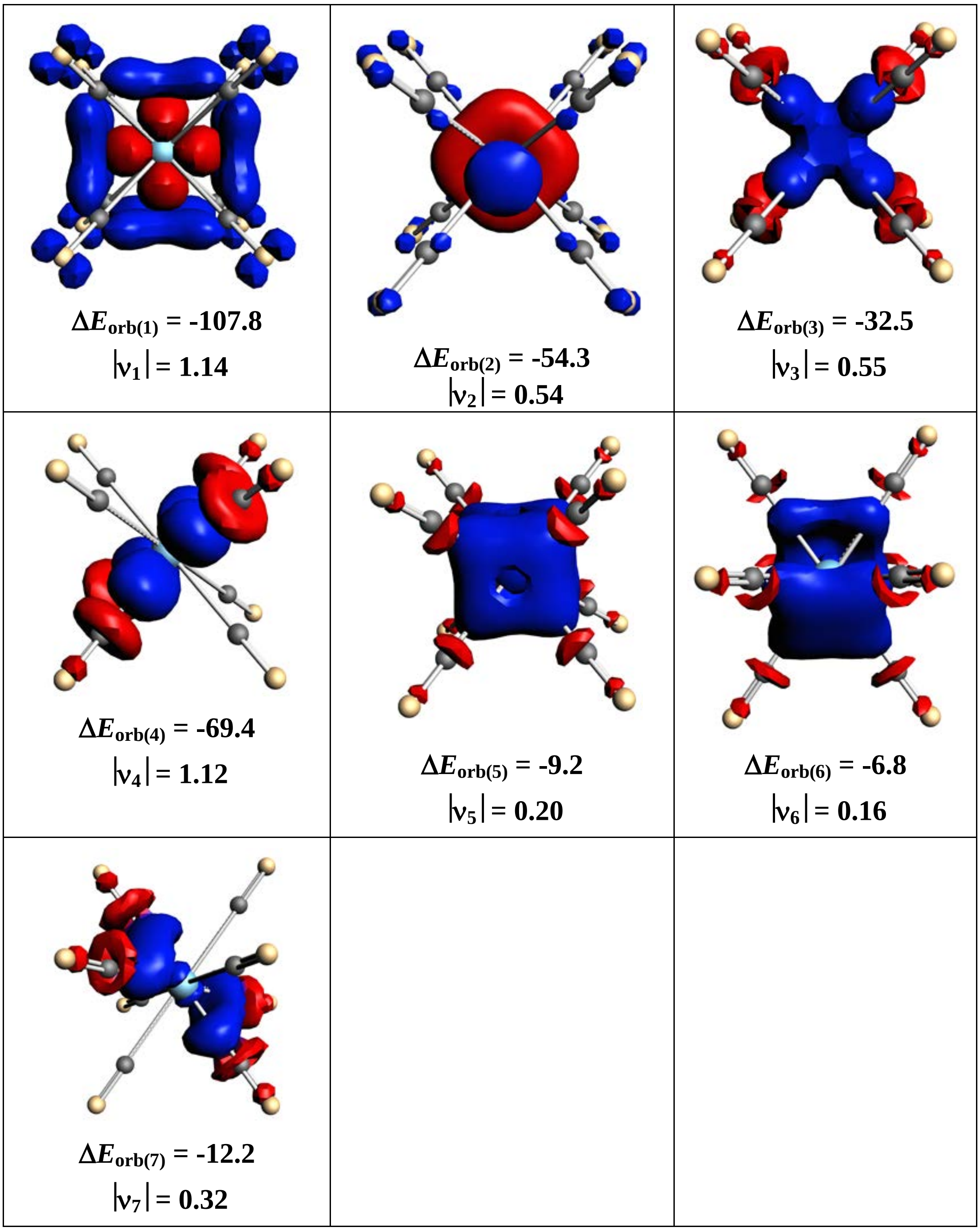

Figure S18. Shape of the deformation densities $\Delta\rho_{(1)\text{-}(7)}$, which are associated with the orbital interactions $\Delta E_{orb(1)\text{-}(7)}$ in $Ti(CO)_8^+$ and eigenvalues $|\nu_n|$ of the charge flow. The isosurface values are 0.002 for $\Delta\rho_{(1)\text{-}(4)}$ and 0.0008 for others. Only one component of the degenerate orbital interactions is shown. The color code of the charge flow is red → blue.

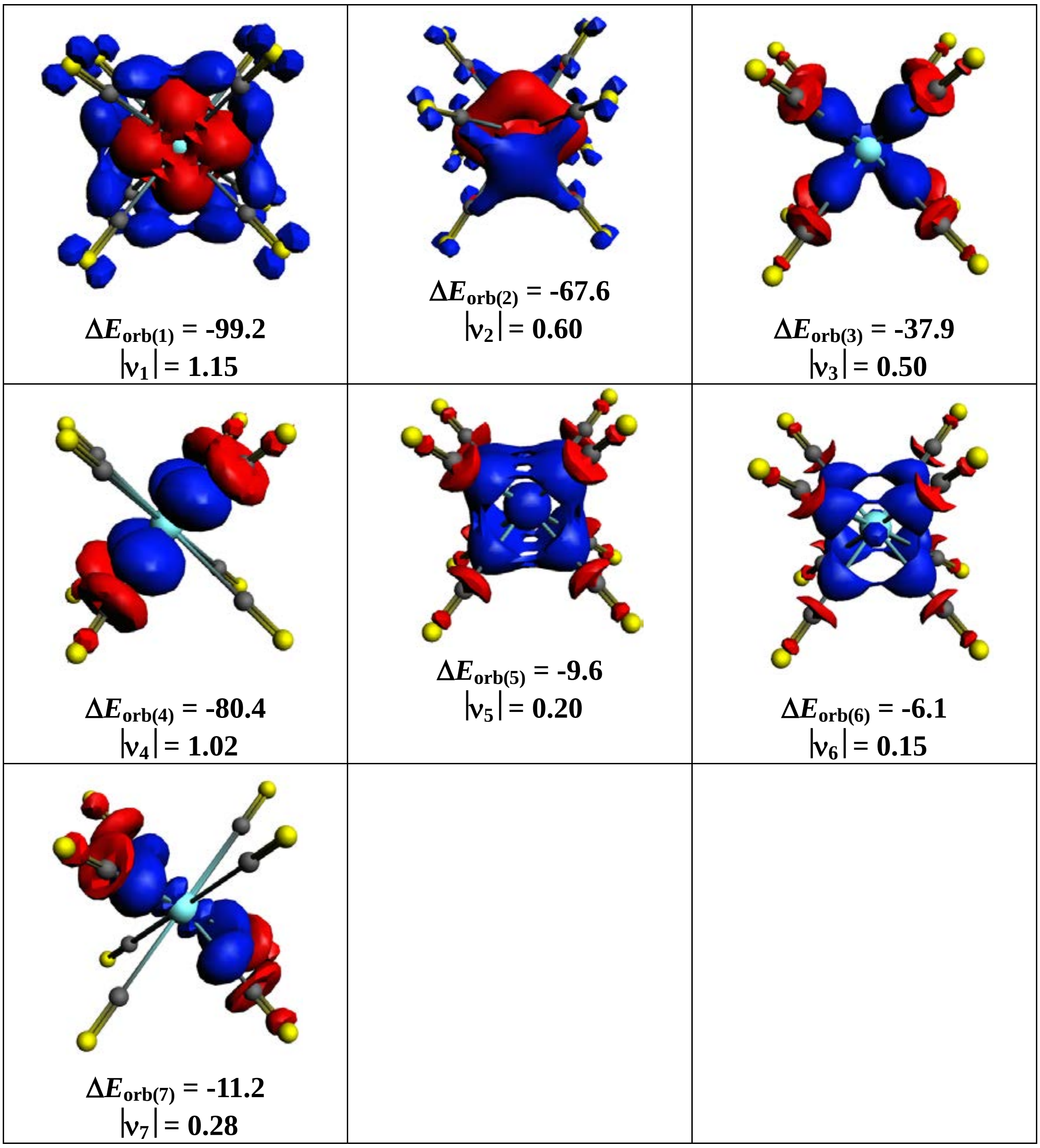

Figure S19. Shape of the deformation densities $\Delta\rho_{(1)\text{-}(7)}$, which are associated with the orbital interactions $\Delta E_{orb(1)\text{-}(7)}$ in $Zr(CO)_8^+$ and eigenvalues $|\nu_n|$ of the charge flow. The isosurface values are 0.0015 for $\Delta\rho_{(1)}$, 0.002 for $\Delta\rho_{(2)\text{-}(4)}$ and 0.0008 for others. Only one component of the degenerate orbital interactions is shown. The color code of the charge flow is red → blue.

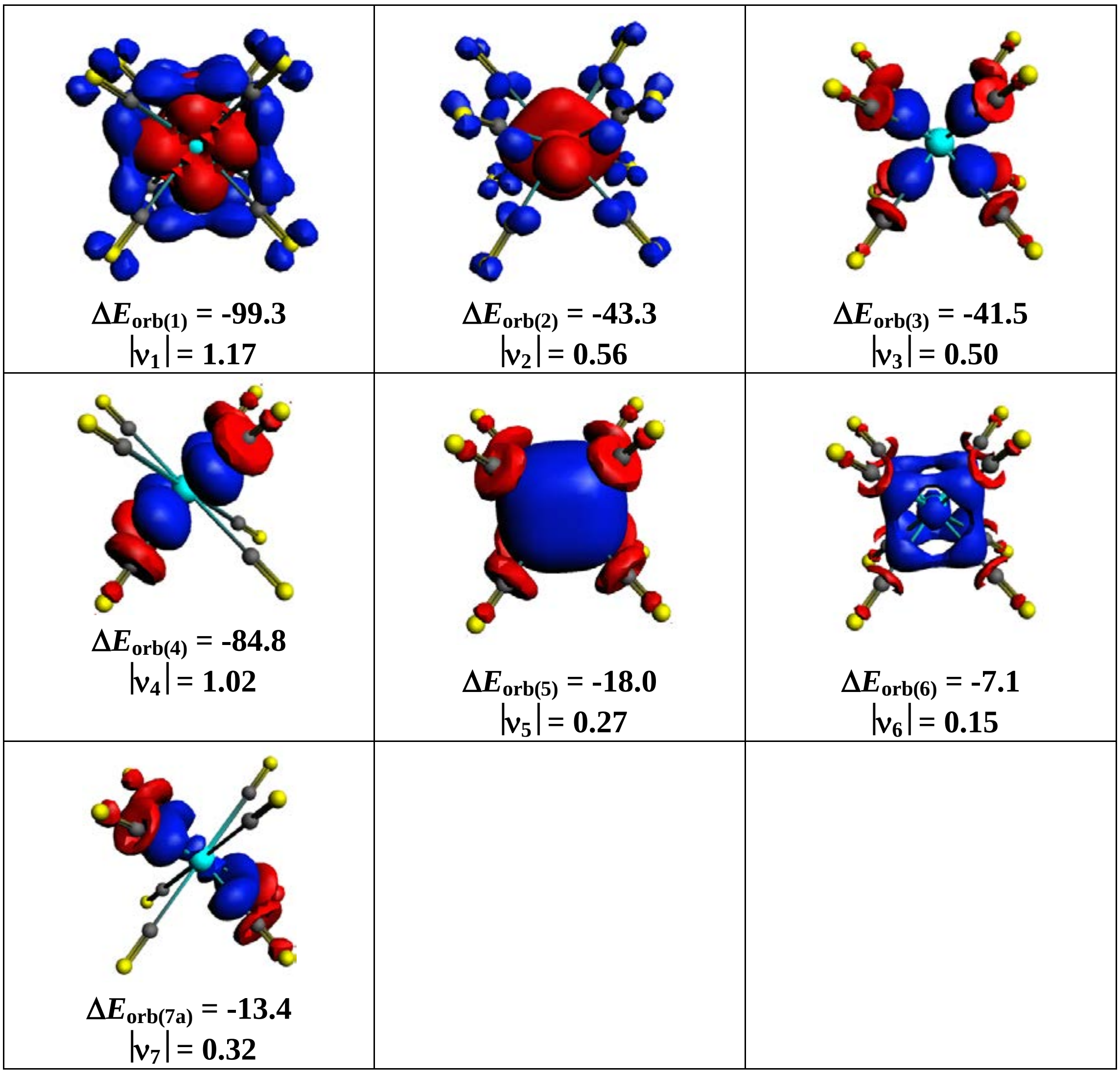

Figure S20. Shape of the deformation densities $\Delta\rho_{(1)\text{-}(7)}$, which are associated with the orbital interactions $\Delta E_{orb(1)\text{-}(7)}$ in $Hf(CO)_8^+$ and eigenvalues $|\nu_n|$ of the charge flow. The isosurface values are 0.0015 for $\Delta\rho_{(1)}$, 0.002 for $\Delta\rho_{(2)\text{-}(4)}$ and 0.0008 for others. Only one component of the degenerate orbital interactions is shown. The color code of the charge flow is red → blue.

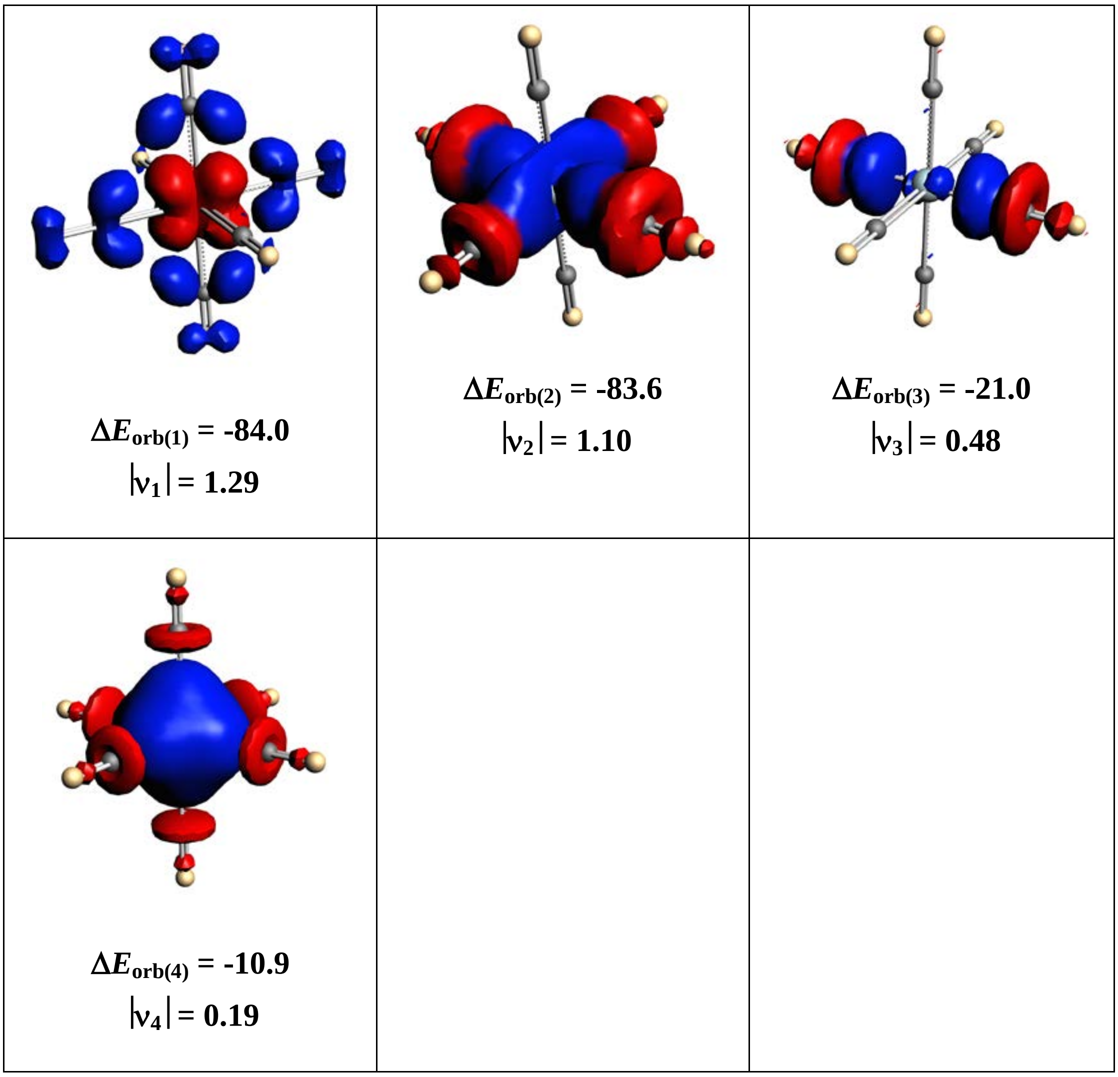

Figure S21. Shape of the deformation densities $\Delta\rho_{(1)-(4)}$, which are associated with the orbital interactions $\Delta E_{orb(1)-(4)}$ in $Ti(CO)_6^+$ and eigenvalues $|\nu_n|$ of the charge flow. The isosurface values are 0.001 for $\Delta\rho_{(1)-(2)}$ and 0.0008 for others Only one component of the degenerate orbital interactions is shown. The color code of the charge flow is red → blue.

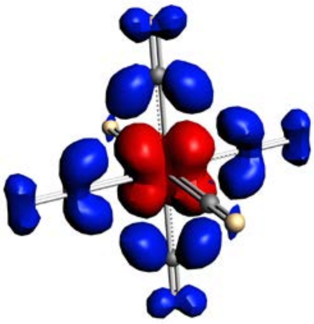<br>**$\Delta E_{orb(1)}$ = -92.4**<br>**$\lvert\nu_1\rvert$ = 1.38**	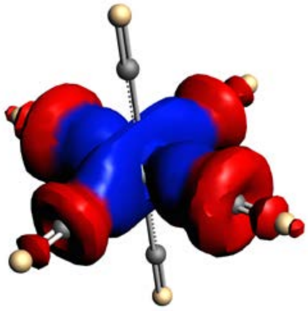<br>**$\Delta E_{orb(2)}$ = -87.4**<br>**$\lvert\nu_2\rvert$ = 1.04**	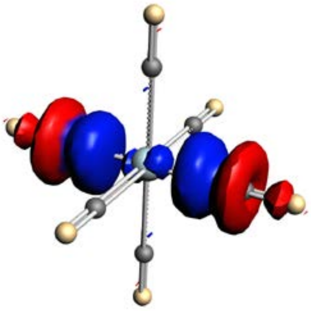<br>**$\Delta E_{orb(3)}$ = -18.9**<br>**$\lvert\nu_3\rvert$ = 0.45**
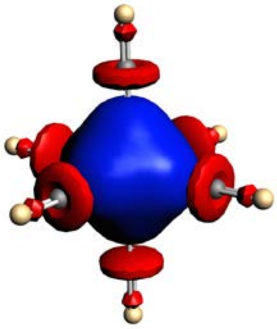<br>**$\Delta E_{orb(4)}$ = -11.6**<br>**$\lvert\nu_4\rvert$ = 0.19**		

Figure S22. Shape of the deformation densities $\Delta\rho_{(1)\text{-}(4)}$, which are associated with the orbital interactions $\Delta E_{orb(1)\text{-}(4)}$ in $Zr(CO)_6^+$ and eigenvalues $\lvert\nu_n\rvert$ of the charge flow. The isosurface values are 0.001 for $\Delta\rho_{(1)\text{-}(2)}$ and 0.0008 for others Only one component of the degenerate orbital interactions is shown. The color code of the charge flow is red → blue.

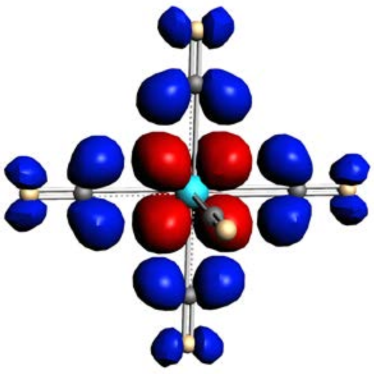<br>**$\Delta E_{orb(1)}$ = -97.3**<br>**$\|\nu_1\|$ = 1.47**	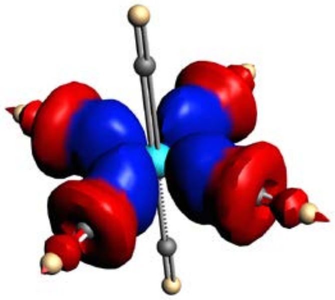<br>**$\Delta E_{orb(2)}$ = -95.2**<br>**$\|\nu_2\|$ = 1.04**	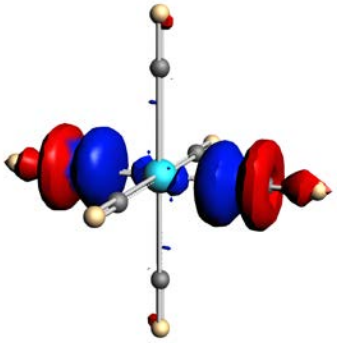<br>**$\Delta E_{orb(3)}$ = -21.9**<br>**$\|\nu_3\|$ = 0.51**
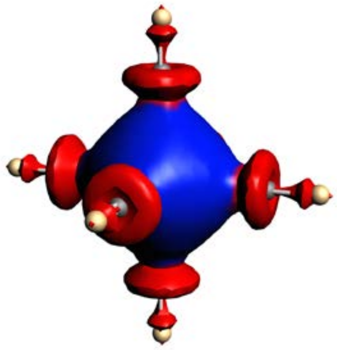<br>**$\Delta E_{orb(4)}$ = -19.5**<br>**$\|\nu_4\|$ = 0.26**		

Figure S23. Shape of the deformation densities $\Delta\rho_{(1)\text{-}(4)}$, which are associated with the orbital interactions $\Delta E_{orb(1)\text{-}(4)}$ in $Hf(CO)_6^+$ and eigenvalues $|\nu_n|$ of the charge flow. The isosurface values are 0.001 for $\Delta\rho_{(1)\text{-}(2)}$ and 0.0008 for others Only one component of the degenerate orbital interactions is shown. The color code of the charge flow is red → blue.

Table S1. The computed unscaled vibrational frequencies (in cm^{-1}) and the corresponding IR intensities (km/mol) for all directly CO coordinate complexes at the M06-D3/def2-TZVPP level.

Complex	*ν*	*I*	Complex	*ν*	*I*
$Ti(CO)_8$ (O_h, $^1A_{1g}$)	36.0	0	$Ti(CO)_6$ (D_{3d}, $^3A_{1g}$)	25.7	0
	36.0	0		40.1	0
	76.7	0		40.1	0
	76.7	0		52.5	0
	76.7	0		52.5	0
	82.4	0		70.9	0
	82.4	0		72.8	1
	82.4	0		72.8	1
	83.4	9		76.0	3
	83.4	9		245.8	0
	83.4	9		245.8	0
	83.7	0		288.3	0
	83.7	0		308.6	0
	217.7	0		308.6	0
	218.9	0		311.0	0
	218.9	0		350.6	0
	218.9	0		355.3	10
	231.0	55		355.3	10
	231.0	55		376.8	0
	231.0	55		376.8	0
	282.9	0		377.9	32
	282.9	0		416.9	7
	282.9	0		416.9	7
	283.6	0		429.3	0
	464.8	0		494.2	89
	464.8	0		494.3	89
	464.8	0		542.7	94
	481.8	0		2048.3	0
	481.8	0		2048.3	0
	502.7	0		2091.1	2576
	502.7	0		2092.5	2014
	521.0	0		2092.5	2014
	521.0	0		2194.0	0
	521.0	0			
	530.4	28			
	530.4	28			
	530.4	28			
	2085.1	2377			
	2085.1	2377			

	2085.1	2377			
	2099.3	0			
	2099.6	0			
	2099.6	0			
	2099.6	0			
	2189.7	0			
$Zr(CO)_8$ (O_h, $^1A_{1g}$)	46.9	0	$Zr(CO)_6$ (D_{3d}, $^3A_{1g}$)	15.3	0
	46.9	0		44.4	0
	74.3	0		44.4	0
	74.3	0		55.6	0
	74.3	0		55.6	0
	77.3	0		58.2	1
	77.3	0		58.2	1
	77.3	0		59.5	0
	78.7	4		62.4	1
	78.7	4		268.3	0
	78.7	4		268.3	0
	79.2	0		273.2	0
	79.2	0		290.3	0
	241.2	0		290.3	0
	252.7	0		303.5	28
	252.7	0		303.5	28
	252.7	0		310.9	50
	253.2	45		311.4	0
	253.2	45		337.0	0
	253.2	45		348.6	0
	285.5	0		348.6	0
	289.4	0		380.9	12
	289.4	0		380.9	12
	289.4	0		382.3	0
	417.5	0		441.0	39
	417.5	0		441.0	39
	417.5	0		476.2	43
	437.1	0		2071.5	0
	437.1	0		2071.5	0
	453.5	0		2089.0	2271
	453.5	0		2089.0	2271
	466.9	20		2089.9	2905
	466.9	20		2190.3	0
	466.9	20			
	475.7	0			
	475.7	0			
	475.7	0			
	2100.5	2607			

	2100.5	2607			
	2100.5	2607			
	2108.6	0			
	2109.5	0			
	2109.5	0			
	2109.5	0			
	2198.2	0			
$Hf(CO)_8$ (O_h, $^1A_{1g}$)	47.4	0	$Hf(CO)_6$ (D_{3d}, $^3A_{1g}$)	14.5	0
	47.4	0		46.3	0
	76.0	3		46.3	0
	76.0	3		56.7	1
	76.0	3		56.7	1
	76.6	0		58.0	0
	76.6	0		58.0	0
	76.6	0		62.8	0
	79.8	0		63.0	1
	79.8	0		282.2	0
	79.8	0		282.2	0
	81.7	0		287.6	0
	81.7	0		294.9	50
	238.0	44		294.9	34
	238.0	44		294.9	34
	238.0	44		305.2	0
	242.9	0		305.2	0
	261.0	0		327.5	0
	261.0	0		347.5	0
	261.0	0		355.6	0
	298.9	0		355.6	0
	300.5	0		378.7	0
	300.5	0		385.1	8
	300.5	0		385.2	8
	430.8	0		442.1	24
	430.8	0		442.1	24
	430.8	0		473.2	27
	433.2	0		2062.9	0
	433.2	0		2062.9	0
	458.2	9		2078.3	2371
	458.2	9		2078.3	2371
	458.2	9		2079.4	3026
	463.0	0		2187.4	0
	463.0	0			
	486.4	0			
	486.4	0			
	486.4	0			

	2094.4	2700			
	2094.4	2700			
	2094.4	2700			
	2101.1	0			
	2103.6	0			
	2103.6	0			
	2103.6	0			
	2197.9	0			
$Zr(CO)_8$ (D_{4d}, $^3A_1$)	35.5	0	$Ti(CO)_6$ (D_{3d}, $^1A_{1g}$)	48.9	2
	52.5	0		48.9	2
	52.5	0		62.7	1
	60.0	1		71.5	0
	60.0	1		73.6	0
	62.5	14		73.9	0
	62.5	14		73.9	0
	65.7	0		82.3	3
	69.2	0		82.3	3
	69.2	0		281.7	0
	75.7	0		281.8	0
	75.8	0		313.0	0
	76.7	2		313.0	0
	216.8	79		321.6	0
	216.9	79		341.8	0
	233.5	0		357.7	38
	233.5	0		357.7	38
	242.2	0		358.5	2
	242.4	0		401.2	0
	266.2	0		420.6	4
	266.3	0		420.7	4
	269.9	0		438.3	44
	272.3	0		442.5	0
	301.6	0		442.5	0
	358.0	7		492.1	0
	358.0	7		571.9	98
	374.8	0		571.9	98
	378.2	0		2055.3	0
	378.2	0		2055.4	0
	394.6	0		2076.3	2535
	394.7	0		2076.4	2535
	412.3	0		2082.2	1395
	412.4	0		2183.6	0
	426.1	0			
	443.4	21			
	443.4	21			

	447.9	0			
	2043.6	2			
	2043.6	2			
	2052.2	3563			
	2052.2	3563			
	2069.8	0			
	2069.8	0			
	2117.1	1716			
	2176.2	0			
$Hf(CO)_8$ (D_{4d}, $^3A_1$)	37.6	0	$Zr(CO)_6$ (D_{3d}, $^1A_{1g}$)	49.6	1
	54.1	0		52.8	1
	54.2	0		52.8	1
	61.6	0		69.7	0
	61.6	0		72.7	0
	66.5	10		73.2	0
	66.5	10		73.2	0
	68.7	0		79.9	2
	71.4	0		79.9	2
	71.4	0		277.8	0
	74.0	2		277.8	0
	78.0	0		306.8	13
	78.1	0		309.4	0
	217.1	62		309.4	0
	217.2	62		310.9	55
	246.2	0		310.9	55
	246.2	0		312.2	0
	251.0	0		334.7	0
	251.0	0		365.0	0
	256.8	1		383.1	16
	281.7	0		390.1	0
	281.8	0		390.1	0
	287.1	0		408.6	0
	317.6	0		408.6	0
	372.9	8		479.2	0
	372.9	8		508.3	43
	379.6	0		508.3	43
	397.5	0		2042.4	0
	397.5	0		2042.4	0
	406.2	0		2056.7	2855
	406.3	0		2056.7	2855
	412.3	0		2058.3	1385
	412.5	0		2158.9	0
	422.2	0			
	439.5	1			

	451.1	15			
	451.2	15			
	2037.0	0			
	2037.0	0			
	2047.5	3643			
	2047.5	3644			
	2064.1	0			
	2064.1	0			
	2112.5	1749			
	2178.2	0			
$Ti(CO)_8^+$ (D_{4h}, $^2A_{1g}$)	-28.4	0	$Hf(CO)_6$ (D_{3d}, $^1A_{1g}$)	40.6	1
	35.3	0		47.0	1
	71.3	0		47.0	1
	71.3	0		67.6	0
	74.5	0		67.6	0
	81.0	0		68.1	0
	81.1	0		68.5	0
	82.4	0		71.2	1
	82.4	0		71.2	1
	85.2	0		286.9	0
	88.8	6		286.9	0
	88.8	6		297.5	51
	89.4	5		297.5	51
	164.1	30		301.5	16
	164.7	0		309.8	0
	174.7	0		309.8	0
	174.7	0		328.3	0
	191.3	22		339.3	0
	191.3	22		372.0	0
	192.7	0		375.1	12
	240.4	0		380.8	1
	240.4	0		380.8	1
	248.7	0		388.0	0
	261.7	0		388.0	0
	282.1	0		481.3	0
	396.4	0		499.0	30
	410.8	0		499.0	30
	410.8	0		2044.4	0
	427.2	11		2044.4	0
	433.9	0		2061.2	2927
	458.8	1		2061.2	2927
	458.8	1		2061.7	1531
	461.3	0		2170.3	0
	468.1	0			

	487.0	0			
	489.2	20			
	489.2	20			
	2181.8	1403			
	2186.4	1353			
	2186.4	1353			
	2196.0	0			
	2196.0	0			
	2196.4	0			
	2197.2	0			
	2252.1	0			
$Ti(CO)_8^+$ (D_{2d}, $^2A_1$)	25.7	0	$Ti(CO)_6^+$ (O_h, $^4A_{1g}$)	45.6	0
	29.7	0		45.6	0
	67.3	1		45.6	0
	67.3	1		66.2	0
	72.1	0		66.2	0
	78.9	0		66.2	0
	82.2	0		76.1	2
	84.8	1		76.1	2
	84.8	1		76.1	2
	86.0	0		261.9	0
	88.1	7		261.9	0
	88.1	7		261.9	0
	91.3	7		262.8	0
	161.6	10		262.8	0
	161.6	10		271.3	0
	181.1	0		310.5	11
	195.1	26		310.5	11
	202.0	1		310.5	11
	218.2	15		343.3	0
	218.2	15		343.3	0
	240.2	0		343.3	0
	249.3	1		355.0	0
	249.3	1		355.0	0
	266.7	0		355.0	0
	330.1	0		418.0	54
	386.4	0		418.0	54
	392.2	10		418.0	54
	406.3	0		2212.9	1139
	406.3	0		2212.9	1139
	426.6	9		2212.9	1139
	456.4	0		2227.0	0
	460.3	2		2227.0	0
	460.3	2		2274.4	0

	473.2	0			
	477.5	0			
	488.1	25			
	488.1	25			
	2175.5	907			
	2175.9	1299			
	2175.9	1299			
	2194.3	0			
	2196.2	145			
	2196.2	145			
	2197.0	551			
	2249.2	0			
$Zr(CO)_8^+$ (D_{4h}, $^2A_{1g}$)	18.1	0	$Zr(CO)_6^+$ (O_h, $^4A_{1g}$)	40.0	0
	45.2	0		40.0	0
	70.8	0		40.0	0
	71.2	0		60.5	0
	71.2	0		60.5	0
	75.0	0		60.5	0
	77.6	0		63.7	1
	77.9	1		63.7	1
	77.9	1		63.7	1
	81.1	0		259.1	0
	81.4	3		259.1	0
	82.0	3		259.1	0
	82.0	3		268.3	0
	206.3	0		268.3	0
	213.0	34		268.4	31
	222.1	0		268.4	31
	222.1	0		268.4	31
	224.6	29		289.5	0
	224.6	29		327.9	0
	230.6	0		327.9	0
	254.4	0		327.9	0
	254.4	0		336.2	0
	265.7	0		336.2	0
	273.7	0		336.2	0
	309.3	0		378.7	34
	362.6	0		378.7	34
	381.7	0		378.7	34
	381.7	0		2194.6	1496
	382.9	11		2194.6	1496
	405.5	0		2194.6	1496
	409.0	0		2212.4	0
	410.6	2		2212.4	0

	410.6	2		2267.8	0
	426.3	0			
	449.8	17			
	449.8	17			
	453.5	0			
	2184.0	1654			
	2187.1	1638			
	2187.1	1638			
	2195.8	0			
	2195.8	0			
	2196.3	0			
	2196.7	0			
	2256.9	0			
$Hf(CO)_8^+$ (D_{4h}, $^2A_{1g}$)	14.1	0	$Hf(CO)_6^+$ (O_h, $^4A_{1g}$)	41.6	0
	45.5	0		41.6	0
	73.6	0		41.6	0
	73.6	0		62.8	0
	74.0	0		62.8	0
	77.5	2		62.8	0
	78.7	0		63.4	0
	79.0	2		63.4	0
	79.0	2		63.4	0
	80.5	0		258.0	46
	81.6	0		258.0	46
	81.6	0		258.0	46
	84.2	0		273.5	0
	201.6	40		273.5	0
	208.2	0		273.5	0
	211.3	36		283.3	0
	211.3	36		283.3	0
	230.5	0		307.9	0
	230.5	0		333.3	0
	239.6	0		333.3	0
	266.0	0		333.3	0
	266.0	0		347.2	0
	280.9	0		347.2	0
	286.5	0		347.2	0
	319.9	0		381.6	24
	378.8	0		381.6	24
	382.8	5		381.6	24
	396.8	0		2180.0	1692
	396.8	0		2180.0	1692
	405.9	0		2180.0	1692
	413.4	0		2200.5	0

	415.5	2		2200.5	0
	415.5	2		2264.0	0
	438.5	0			
	455.7	10			
	455.7	10			
	466.1	0			
	2174.6	1790			
	2177.6	1776			
	2177.6	1776			
	2186.5	0			
	2187.1	0			
	2187.1	0			
	2187.8	0			
	2255.2	0			
$Ti(CO)_7$ (C_{3v}, $^1A_1$)	34.1	1	$Ti(CO)_6^+$ (C_{2h}, ^2A_g)	35.5	2
	34.1	1		49.4	1
	74.5	0		56.4	0
	75.9	0		60.6	0
	80.2	0		63.6	2
	80.2	0		65.0	1
	84.2	5		71.0	0
	84.2	5		77.3	2
	89.3	4		79.9	3
	90.7	1		218.2	0
	90.7	1		257.1	0
	277.2	7		267.4	0
	277.3	7		271.5	0
	302.7	0		278.1	5
	322.3	1		286.7	0
	322.3	1		296.7	0
	324.2	1		316.7	25
	337.1	0		321.4	4
	366.3	28		340.0	7
	366.3	28		348.1	0
	379.6	7		372.2	34
	393.5	3		373.9	0
	393.5	3		379.6	0
	458.5	17		383.5	0
	474.4	0		418.3	1
	474.4	0		467.6	58
	498.0	0		512.6	71
	527.4	1		2169.2	0
	527.4	1		2192.1	1310
	565.5	63		2193.8	0

	578.9	74		2201.9	1440
	578.9	73		2206.7	885
	2073.2	988		2268.3	0
	2074.4	1657			
	2074.4	1657			
	2098.3	1083			
	2118.1	731			
	2118.1	731			
	2194.7	90			
$Zr(CO)_7$ (C_{3v}, $^1A_1$)	37.5	1	$Zr(CO)_6^+$ (C_{2v}, $^2B_1$)	37.9	0
	37.5	1		44.7	0
	57.0	1		56.8	1
	66.5	0		63.7	4
	69.4	2		69.0	0
	69.4	2		70.5	0
	71.8	2		73.2	1
	71.8	2		75.6	3
	77.6	2		86.8	0
	81.2	1		246.1	0
	81.2	1		246.2	30
	258.0	14		249.8	7
	258.0	14		271.8	7
	285.9	5		272.9	16
	296.7	2		281.9	1
	296.7	2		286.8	0
	299.2	0		293.1	1
	309.0	0		298.8	0
	315.9	29		308.7	25
	315.9	30		345.5	14
	321.2	8		345.6	4
	363.8	0		354.1	16
	363.9	0		381.2	0
	375.5	19		395.0	8
	411.2	0		444.7	15
	411.2	0		458.0	5
	460.7	0		463.3	28
	468.9	24		2159.3	713
	473.5	2		2164.7	987
	473.5	2		2182.6	1779
	484.6	33		2194.1	1033
	484.6	33		2195.3	0
	2079.2	890		2253.5	4
	2084.4	1663			
	2084.5	1663			

	2099.8	1309			
	2113.9	999			
	2113.9	999			
	2195.4	51			
$Hf(CO)_7$ (C_{3v}, $^1A_1$)	37.0	1	$Hf(CO)_6^+$ (C_{2v}, $^2B_1$)	37.3	0
	37.1	1		46.1	0
	58.0	1		54.8	1
	68.3	0		56.5	3
	68.4	3		69.4	0
	68.5	3		72.7	0
	72.3	0		73.1	0
	72.4	0		74.7	2
	76.8	1		86.6	0
	82.1	0		245.1	43
	82.3	0		257.2	0
	264.5	21		257.7	13
	264.5	22		280.1	13
	291.0	10		283.2	14
	303.2	15		288.8	22
	303.5	14		296.2	0
	311.2	9		301.2	10
	311.3	10		304.5	0
	311.5	4		310.9	0
	320.4	9		325.1	3
	321.7	0		339.4	14
	363.4	1		356.6	3
	363.6	1		392.2	0
	375.6	13		398.1	6
	410.4	1		456.7	13
	410.5	0		458.1	4
	463.3	10		462.9	19
	469.2	0		2148.1	795
	472.6	21		2156.3	1094
	472.8	21		2173.4	1904
	477.4	0		2184.1	1148
	477.8	0		2185.0	0
	2076.2	876		2252.3	5
	2080.5	1713			
	2080.6	1711			
	2094.2	1432			
	2106.6	1019			
	2106.6	1017			
	2195.6	44			
$Ti(CO)_7^+$ (C_s, $^2A'$)	18.8	1			

	24.3	0		
	71.2	1		
	74.3	0		
	75.9	0		
	75.9	0		
	86.0	1		
	86.9	3		
	88.8	3		
	90.8	3		
	92.3	2		
	228.3	2		
	250.9	4		
	268.4	1		
	273.3	0		
	273.6	0		
	280.6	3		
	295.4	7		
	301.7	2		
	314.5	6		
	318.8	19		
	338.4	11		
	342.8	0		
	387.7	9		
	415.0	4		
	424.6	2		
	439.1	0		
	458.5	36		
	474.4	4		
	485.5	4		
	510.6	55		
	514.5	55		
	2174.6	790		
	2178.0	1058		
	2183.0	986		
	2198.6	512		
	2212.7	416		
	2213.4	321		
	2261.5	63		
$Zr(CO)_7^+$ (C_s, $^2A'$)	13.7	2		
	37.5	0		
	51.9	1		
	58.5	1		
	65.8	0		
	70.5	1		

	71.6	1		
	73.7	2		
	75.2	0		
	76.4	2		
	82.1	1		
	232.3	13		
	234.7	2		
	253.7	1		
	255.6	1		
	263.8	18		
	267.7	10		
	277.3	2		
	278.9	7		
	289.7	4		
	292.7	17		
	295.8	3		
	338.7	0		
	341.1	13		
	349.3	5		
	356.1	2		
	392.3	18		
	398.1	3		
	416.0	4		
	434.7	1		
	436.4	28		
	445.1	29		
	2173.2	817		
	2177.2	1234		
	2185.6	1244		
	2194.4	699		
	2198.2	441		
	2208.8	362		
	2260.8	43		
$Hf(CO)_7^+$ (C_{2v}, $^2A_1$)	8.9	2		
	40.8	0		
	55.3	2		
	59.4	1		
	66.6	0		
	71.3	1		
	73.7	1		
	75.0	1		
	75.6	0		
	75.7	0		
	83.7	0		

	238.6	24			
	248.2	0			
	254.6	43			
	263.2	0			
	264.8	8			
	279.1	0			
	283.2	13			
	283.7	23			
	295.4	1			
	297.5	0			
	300.9	2			
	346.6	0			
	347.8	13			
	353.2	3			
	358.8	0			
	400.2	6			
	404.9	2			
	412.8	7			
	438.9	13			
	445.0	0			
	450.5	23			
	2166.2	916			
	2169.8	1319			
	2179.6	1838			
	2185.1	735			
	2185.3	0			
	2200.5	389			
	2259.8	38			

Table S2. Coordinates and energies of the optimized structures at M06-D3/def2-TZVPP.

$Ti(CO)_8$ (O_h, $^1A_{1g}$)
E = -1756.014646 au

C	1.276283000	1.276283000	1.276283000
C	1.276283000	1.276283000	-1.276283000

C	-1.276283000	1.276283000	1.276283000
C	-1.276283000	-1.276283000	-1.276283000
C	-1.276283000	-1.276283000	1.276283000
C	1.276283000	-1.276283000	1.276283000
C	-1.276283000	1.276283000	-1.276283000
C	1.276283000	-1.276283000	-1.276283000
O	-1.931659000	-1.931659000	-1.931659000
O	-1.931659000	1.931659000	-1.931659000
O	1.931659000	1.931659000	-1.931659000
O	1.931659000	1.931659000	1.931659000
O	1.931659000	-1.931659000	1.931659000
O	1.931659000	-1.931659000	-1.931659000
O	-1.931659000	-1.931659000	1.931659000
O	-1.931659000	1.931659000	1.931659000
Ti	0.000000000	0.000000000	0.000000000

$Ti(CO)_7$ ···CO (C_s, $^1A'$)
E = -1756.02939 au

C	-1.531919000	1.897171000	-0.000000000
C	0.651415000	1.857539000	1.508104000
C	1.994130000	-0.375012000	0.000000000
C	0.651415000	1.857539000	-1.508104000
C	0.024895000	-0.708847000	1.745276000
C	0.024895000	-0.708847000	-1.745276000
O	3.032529000	-0.826082000	0.000000000
O	0.060335000	-1.339574000	2.692534000
O	-2.344308000	2.694319000	-0.000000000
O	0.984039000	2.590102000	-2.305907000
O	0.060335000	-1.339574000	-2.692534000
O	0.984039000	2.590102000	2.305907000
C	-1.768887000	-0.605512000	-0.000000000
O	-2.738188000	-1.205664000	-0.000000000
Ti	0.016307000	0.468890000	-0.000000000
C	0.467870000	-3.567996000	0.000000000
O	-0.468987000	-4.187604000	0.000000000

$Ti(CO)_6$ ···2CO (C_{2v}, $^3B_2$)
E = -1756.004879 au

C	-1.613900000	1.412796000	0.575963000
C	-1.613900000	-1.412796000	0.575963000
C	1.613900000	-1.412796000	0.575963000
C	-0.000000000	-0.000000000	2.692782000
C	0.000000000	0.000000000	-1.541003000

C	1.613900000	1.412796000	0.575963000
O	2.481127000	-2.144251000	0.574179000
O	0.000000000	0.000000000	-2.679297000
O	-2.481127000	2.144251000	0.574179000
O	-0.000000000	-0.000000000	3.830282000
O	2.481127000	2.144251000	0.574179000
O	-2.481127000	-2.144251000	0.574179000
C	0.000000000	3.497655000	-1.730587000
O	0.000000000	4.301975000	-2.513211000
Ti	-0.000000000	-0.000000000	0.575593000
C	-0.000000000	-3.497655000	-1.730587000
O	-0.000000000	-4.301975000	-2.513211000

$Zr(CO)_8$ (O_h, $^1A_{1g}$)
E = -953.639838 au

C	1.356199000	1.356199000	1.356199000
C	1.356199000	-1.356199000	1.356199000
C	1.356199000	1.356199000	-1.356199000
C	-1.356199000	-1.356199000	-1.356199000
C	-1.356199000	1.356199000	-1.356199000
C	-1.356199000	1.356199000	1.356199000
C	1.356199000	-1.356199000	-1.356199000
C	-1.356199000	-1.356199000	1.356199000
O	-2.010895000	-2.010895000	-2.010895000
O	2.010895000	-2.010895000	-2.010895000
O	2.010895000	-2.010895000	2.010895000
O	2.010895000	2.010895000	2.010895000
O	-2.010895000	2.010895000	2.010895000
O	-2.010895000	-2.010895000	2.010895000
O	-2.010895000	2.010895000	-2.010895000
O	2.010895000	2.010895000	-2.010895000
Zr	0.000000000	0.000000000	0.000000000

$Zr(CO)_7\cdots CO$ (C_s, $^1A'$)
E = -953.621648 au

C	-1.934564000	1.683627000	-0.000000000
C	0.307751000	2.001102000	1.713040000
C	2.221645000	-0.245300000	0.000000000
C	0.307751000	2.001102000	-1.713040000
C	0.179346000	-0.830607000	1.881094000
C	0.179346000	-0.830607000	-1.881094000
O	3.300695000	-0.586019000	0.000000000
O	0.307751000	-1.461028000	2.818731000

O	-2.869742000	2.329706000	-0.000000000
O	0.464377000	2.753705000	-2.544820000
O	0.307751000	-1.461028000	-2.818731000
O	0.464377000	2.753705000	2.544820000
C	-1.719861000	-0.996863000	0.000000000
O	-2.578271000	-1.744834000	0.000000000
Zr	-0.006491000	0.453618000	-0.000000000
C	1.054372000	-3.556001000	0.000000000
O	0.188679000	-4.272137000	0.000000000

$Zr(CO)_6 \cdots 2CO$ (C_s, $^3A''$)

E = -953.585161 au

C	-0.694743000	1.757007000	1.532599000
C	-0.694743000	1.757007000	-1.532599000
C	-0.332125000	-1.689696000	-1.530078000
C	-2.733831000	-0.364298000	0.000000000
C	1.782490000	0.422840000	-0.000000000
C	-0.332125000	-1.689696000	1.530078000
O	-0.284193000	-2.548616000	-2.270721000
O	2.900536000	0.631903000	-0.000000000
O	-0.802404000	2.615300000	2.264504000
O	-3.849582000	-0.578963000	0.000000000
O	-0.284193000	-2.548616000	2.270721000
O	-0.802404000	2.615300000	-2.264504000
C	1.740633000	0.251557000	3.381281000
O	2.573096000	-0.431308000	3.700510000
C	1.740633000	0.251557000	-3.381281000
O	2.573096000	-0.431308000	-3.700510000
Zr	-0.476219000	0.030820000	0.000000000

$Zr(CO)_8$ (D_{4d}, $^3A_1$)

E = -953.578293 au

C	-0.722740000	1.748670000	-1.392625000
C	-1.748670000	0.722740000	1.392625000
C	1.748670000	0.722740000	-1.392625000
C	1.748670000	-0.722740000	1.392625000
C	0.722740000	-1.748670000	-1.392625000
C	-1.748670000	-0.722740000	-1.392625000
C	0.722740000	1.748670000	1.392625000
C	-0.722740000	-1.748670000	1.392625000
O	2.610983000	-1.085351000	2.038750000

O	1.085351000	2.610983000	2.038750000
O	-2.610983000	1.085351000	2.038750000
O	-1.085351000	2.610983000	-2.038750000
O	-2.610983000	-1.085351000	-2.038750000
O	-1.085351000	-2.610983000	2.038750000
O	1.085351000	-2.610983000	-2.038750000
O	2.610983000	1.085351000	-2.038750000
Zr	-0.000000000	0.000000000	0.000000000

$Hf(CO)_8$ (O_h, $^1A_{1g}$)

E = -954.604401 au

C	1.348428000	1.348428000	1.348428000
C	1.348428000	-1.348428000	1.348428000
C	1.348428000	1.348428000	-1.348428000
C	-1.348428000	-1.348428000	-1.348428000
C	-1.348428000	1.348428000	-1.348428000
C	-1.348428000	1.348428000	1.348428000
C	1.348428000	-1.348428000	-1.348428000
C	-1.348428000	-1.348428000	1.348428000
O	-2.003578000	-2.003578000	-2.003578000
O	2.003578000	-2.003578000	-2.003578000
O	2.003578000	-2.003578000	2.003578000
O	2.003578000	2.003578000	2.003578000
O	-2.003578000	2.003578000	2.003578000
O	-2.003578000	-2.003578000	2.003578000
O	-2.003578000	2.003578000	-2.003578000
O	2.003578000	2.003578000	-2.003578000
Hf	0.000000000	0.000000000	0.000000000

$Hf(CO)_7 \cdots CO$ (C_s, $^1A'$)

E = -954.58638 au

C	1.848661000	1.689102000	0.000000000
C	-0.410289000	1.890678000	-1.684309000
C	-2.189624000	-0.387215000	-0.000000000
C	-0.410289000	1.890678000	1.684309000
C	-0.138577000	-0.911328000	-1.875546000
C	-0.138577000	-0.911328000	1.875546000
O	-3.259535000	-0.758165000	-0.000000000
O	-0.245927000	-1.542565000	-2.816256000
O	2.746812000	2.387166000	0.000000000
O	-0.616884000	2.640160000	2.509334000

O	-0.245927000	-1.542565000	2.816256000
O	-0.616884000	2.640160000	-2.509334000
C	1.774223000	-1.001430000	-0.000000000
O	2.665325000	-1.710942000	-0.000000000
Hf	0.000000000	0.365107000	0.000000000
C	-0.895983000	-3.661558000	-0.000000000
O	-0.006101000	-4.347541000	-0.000000000

$Hf(CO)_6 \cdots 2CO$ (C_1, ^3A)

E = -954.548286 au

C	-1.508091000	0.685782000	1.717195000
C	1.504717000	0.698290000	1.713530000
C	1.498450000	0.167824000	-1.705886000
C	-0.010218000	2.614431000	-0.445180000
C	0.007090000	-1.836401000	0.457186000
C	-1.503948000	0.159431000	-1.704441000
O	2.237204000	0.074220000	-2.564072000
O	0.012507000	-2.951425000	0.689311000
O	-2.239873000	0.840064000	2.569728000
O	-0.013947000	3.727281000	-0.681040000
O	-2.242924000	0.062238000	-2.562021000
O	2.237690000	0.859397000	2.563779000
C	-3.372871000	-1.770515000	0.311179000
O	-3.662483000	-2.679998000	-0.280593000
C	3.385929000	-1.752485000	0.311160000
O	3.693236000	-2.655016000	-0.282287000
Hf	-0.002467000	0.388719000	0.006238000

$Hf(CO)_8$ (D_{4d}, $^3A_1$)

E = -954.546487 au

C	-1.382993000	1.285289000	1.371895000
C	1.399754000	-0.051768000	1.866692000
C	-1.398410000	1.361308000	-1.279835000
C	1.377761000	0.056306000	-1.882869000
C	-1.393910000	-1.289748000	-1.356140000
C	-1.378168000	-1.366603000	1.295709000
C	1.384848000	1.878246000	0.046370000
C	1.391017000	-1.872858000	-0.061769000
O	2.019357000	0.084030000	-2.821981000

O 2.029771000 2.815180000 0.069987000
O 2.052841000 -0.077787000 2.797910000
O -2.027388000 1.926504000 2.055958000
O -2.020207000 -2.048383000 1.941704000
O 2.038821000 -2.807621000 -0.092230000
O -2.043700000 -1.933096000 -2.033078000
O -2.050611000 2.040246000 -1.918611000
Hf 0.000132000 0.000089000 0.000033000

$Ti(CO)_7$ (C_{3v}, $^1A_1$)
E = -1642.724261 au

C 0.00000000 2.02060544 0.58342525
C 1.50412250 0.86840553 -1.27135497
C 0.00000000 -1.73681106 -1.27135497
C -1.50412250 0.86840553 -1.27135497
C 1.74989564 -1.01030272 0.58342525
C -1.74989564 -1.01030272 0.58342525
O 0.00000000 -2.65635891 -1.93327268
O 2.70386068 -1.56107469 0.87063247
O -0.00000000 3.12214938 0.87063247
O -2.30047430 1.32817946 -1.93327268
O -2.70386068 -1.56107469 0.87063247
O 2.30047430 1.32817946 -1.93327268
C 0.00000000 0.00000000 2.07335512
O 0.00000000 0.00000000 3.21303812
Ti -0.00000000 -0.00000000 -0.01174488

$Zr(CO)_7$ (C_{3v}, $^1A_1$)
E = -840.316078 au

C 0.00000000 2.18403396 -0.61246129
C -1.70266314 0.98303302 1.31374890
C 0.00000000 -1.96606604 1.31374890
C 1.70266314 0.98303302 1.31374890
C -1.89142889 -1.09201698 -0.61246129
C 1.89142889 -1.09201698 -0.61246129
O 0.00000000 -2.92022764 1.92415352
O -2.84204914 -1.64085784 -0.90782568
O -0.00000000 3.28171568 -0.90782568
O 2.52899132 1.46011382 1.92415352
O 2.84204914 -1.64085784 -0.90782568
O -2.52899132 1.46011382 1.92415352
C 0.00000000 0.00000000 -2.18168394
O 0.00000000 0.00000000 -3.31985694

Zr -0.00000000 -0.00000000 0.06584806
$Hf(CO)_7$ (C_{3v}, $^1A_1$)<br>E = -841.280803 au<br><br>C -0.000000000 2.176586000 -0.619133000<br>C -1.671230000 0.964885000 1.309019000<br>C -0.000000000 -1.929770000 1.309019000<br>C 1.671230000 0.964885000 1.309019000<br>C -1.884979000 -1.088293000 -0.619133000<br>C 1.884979000 -1.088293000 -0.619133000<br>O 0.000000000 -2.875906000 1.933529000<br>O -2.837374000 -1.638159000 -0.909327000<br>O 0.000000000 3.276317000 -0.909327000<br>O 2.490608000 1.437953000 1.933529000<br>O 2.837374000 -1.638159000 -0.909327000<br>O -2.490608000 1.437953000 1.933529000<br>C -0.000000000 0.000000000 -2.200644000<br>O -0.000000000 0.000000000 -3.339359000<br>Hf -0.000000000 0.000000000 0.040941000
$Ti(CO)_6\cdots CO$ (C_s, $^3A'$)<br><br>E = -1642.699909 au<br><br>C -0.82974203 -0.55942375 1.61493800<br>C 0.88519797 1.68141625 1.61554500<br>C 0.88519797 1.68141625 -1.61554500<br>C -1.65146203 1.85135425 0.00000000<br>C 1.71045697 -0.72242075 0.00000000<br>C -0.82974203 -0.55942375 -1.61493800<br>O 1.32760797 2.25849225 -2.48630000<br>O 2.61098196 -1.41774575 0.00000000<br>O -1.27025903 -1.14046675 2.48379000<br>O -2.55266703 2.54519525 0.00000000<br>O -1.27025903 -1.14046675 -2.48379000<br>O 1.32760797 2.25849225 2.48630000<br>C -0.28109104 -3.61362975 0.00000000<br>O -0.16705904 -4.73000775 0.00000000<br>Ti 0.02815797 0.56256025 0.00000000
$Zr(CO)_6\cdots CO$ (C_1, ^3A)<br><br>E = -840.279889 au<br><br>C 0.81372400 -1.88711400 -0.29902500<br>C -1.53987900 -1.21034200 1.60274100

C -1.70712200 1.93389400 0.30143100
C -1.91838200 -0.92473300 -1.46991000
C 0.98432200 0.99489700 1.53660000
C 0.63134700 1.27281200 -1.62533100
O -2.31031400 2.88378700 0.44541400
O 1.67853400 1.49376100 2.28467800
O 1.40298300 -2.84564100 -0.44946500
O -2.65313700 -1.41825900 -2.18109600
O 1.14325000 1.90589100 -2.41435100
O -2.08807200 -1.84369000 2.36873000
C 3.60883600 0.08744400 -0.40621400
O 4.34777100 -0.51893800 0.18345800
Zr -0.43513000 0.02858900 0.00648300

$Hf(CO)_6\cdots CO$ (C_1, ^3A)

E = -841.242752 au

C 0.88812100 -1.87183900 -0.32508600
C -1.42514300 -1.23254800 1.58492000
C -1.61559200 1.90753800 0.31077300
C -1.83046000 -0.91592800 -1.45468400
C 1.05029600 0.97390000 1.52957200
C 0.69244100 1.27591200 -1.60229900
O -2.22657000 2.85289500 0.46005900
O 1.74331700 1.46431600 2.28619900
O 1.48642600 -2.82338200 -0.49168200
O -2.57162200 -1.39898200 -2.16833300
O 1.20018200 1.91380700 -2.39166500
O -1.96224500 -1.87272300 2.35474100
C 3.67483600 0.09328900 -0.41463500
O 4.42684400 -0.49913800 0.17271300
Hf -0.35246700 0.02116200 0.00628300

$Ti(CO)_6$ (D_{3d}, $^3A_{1g}$)

E = -1529.394584 au

C 0.00000000 1.68064723 1.31958598
C 1.45548320 0.84032362 -1.31958598
C -0.00000000 -1.68064723 -1.31958598
C -1.45548320 0.84032362 -1.31958598
C 1.45548320 -0.84032362 1.31958598
C -1.45548320 -0.84032362 1.31958598
O 0.00000000 -2.55344581 -2.04533549
O 2.21134894 -1.27672290 2.04533549

O 0.00000000 2.55344581 2.04533549
O -2.21134894 1.27672290 -2.04533549
O -2.21134894 -1.27672290 2.04533549
O 2.21134894 1.27672290 -2.04533549
Ti -0.00000000 0.00000000 0.00000000

$Zr(CO)_6$ (D_{3d}, $^3A_{1g}$)

E = -726.974786 au

C 0.00000000 1.80120200 1.43751300
C 1.55988700 0.90060100 -1.43751300
C 0.00000000 -1.80120200 -1.43751300
C -1.55988700 0.90060100 -1.43751300
C 1.55988700 -0.90060100 1.43751300
C -1.55988700 -0.90060100 1.43751300
O 0.00000000 -2.66979800 -2.16801700
O 2.31211300 -1.33489900 2.16801700
O 0.00000000 2.66979800 2.16801700
O -2.31211300 1.33489900 -2.16801700
O -2.31211300 -1.33489900 2.16801700
O 2.31211300 1.33489900 -2.16801700
Zr 0.00000000 0.00000000 0.00000000

$Hf(CO)_6$ (D_{3d}, $^3A_{1g}$)

E = -727.937479 au

C 0.00000000 1.78346100 1.42790100
C 1.54452200 0.89173000 -1.42790100
C 0.00000000 -1.78346100 -1.42790100
C -1.54452200 0.89173000 -1.42790100
C 1.54452200 -0.89173000 1.42790100
C -1.54452200 -0.89173000 1.42790100
O 0.00000000 -2.65712200 -2.15440800
O 2.30113500 -1.32856100 2.15440800
O 0.00000000 2.65712200 2.15440800
O -2.30113500 1.32856100 -2.15440800
O -2.30113500 -1.32856100 2.15440800
O 2.30113500 1.32856100 -2.15440800
Hf 0.00000000 0.00000000 0.00000000

$Ti(CO)_6$ (D_{3d}, $^1A_{1g}$)

E = -1529.380561 au

C -1.61241900 -0.93093000 -1.01882600<br>C 0.00000000 1.86186100 -1.01882600<br>C 1.61241900 0.93093000 1.01882600<br>C 1.61241900 -0.93093000 -1.01882600<br>C -1.61241900 0.93093000 1.01882600<br>C 0.00000000 -1.86186100 1.01882600<br>O 2.50037600 1.44359300 1.51092700<br>O -2.50037600 1.44359300 1.51092700<br>O -2.50037600 -1.44359300 -1.51092700<br>O 2.50037600 -1.44359300 -1.51092700<br>O 0.00000000 -2.88718500 1.51092700<br>O 0.00000000 2.88718500 -1.51092700<br>Ti 0.00000000 0.00000000 0.00000000
$Zr(CO)_6$ (D_{3d}, $^1A_{1g}$)<br><br>E = -726.963455 au<br><br>C 0.00000000 2.05412700 1.02813100<br>C -1.77892600 -1.02706400 1.02813100<br>C 0.00000000 -2.05412700 -1.02813100<br>C 1.77892600 -1.02706400 1.02813100<br>C -1.77892600 1.02706400 -1.02813100<br>C 1.77892600 1.02706400 -1.02813100<br>O 0.00000000 -3.09439700 -1.48616300<br>O -2.67982700 1.54719900 -1.48616300<br>O 0.00000000 3.09439700 1.48616300<br>O 2.67982700 -1.54719900 1.48616300<br>O 2.67982700 1.54719900 -1.48616300<br>O -2.67982700 -1.54719900 1.48616300<br>Zr 0.00000000 0.00000000 0.00000000
$Hf(CO)_6$ (D_{3d}, $^1A_{1g}$)<br><br>E = -727.925968 au<br><br>C 0.00000000 2.02137200 1.04919100<br>C -1.75055900 -1.01068600 1.04919100<br>C 0.00000000 -2.02137200 -1.04919100<br>C 1.75055900 -1.01068600 1.04919100<br>C -1.75055900 1.01068600 -1.04919100<br>C 1.75055900 1.01068600 -1.04919100<br>O 0.00000000 -3.06131300 -1.51416400<br>O -2.65117400 1.53065600 -1.51416400<br>O 0.00000000 3.06131300 1.51416400<br>O 2.65117400 -1.53065600 1.51416400

O 2.65117400 1.53065600 -1.51416400			
O -2.65117400 -1.53065600 1.51416400			
Hf 0.00000000 0.00000000 0.00000000			
$Ti(CO)_8^+$ (D_{4h}, $^2A_{1g}$) (NIMAG = 1)			
E = -1755.74945 au			
C	0.000000000	1.879459000	1.296452000
C	1.879459000	-0.000000000	1.296452000
C	0.000000000	1.879459000	-1.296452000
C	-0.000000000	-1.879459000	-1.296452000
C	-1.879459000	0.000000000	-1.296452000
C	-1.879459000	0.000000000	1.296452000
C	1.879459000	-0.000000000	-1.296452000
C	-0.000000000	-1.879459000	1.296452000
O	0.000000000	-2.813329000	-1.922098000
O	2.813329000	-0.000000000	-1.922098000
O	2.813329000	-0.000000000	1.922098000
O	0.000000000	2.813329000	1.922098000
O	-2.813329000	0.000000000	1.922098000
O	-0.000000000	-2.813329000	1.922098000
O	-2.813329000	0.000000000	-1.922098000
O	0.000000000	2.813329000	-1.922098000
Ti	0.000000000	0.000000000	0.000000000
$Ti(CO)_8^+$ (D_{2d}, $^2A_1$)			
E = -1755.750395 au			
C	-1.681357000	0.000000000	1.549161000
C	-0.000000000	2.000942000	1.015262000
C	-2.000942000	0.000000000	-1.015262000
C	2.000942000	-0.000000000	-1.015262000
C	-0.000000000	-1.681357000	-1.549161000
C	-0.000000000	-2.000942000	1.015262000
C	0.000000000	1.681357000	-1.549161000
C	1.681357000	-0.000000000	1.549161000
O	3.038918000	-0.000000000	-1.449880000
O	0.000000000	2.457924000	-2.360968000
O	0.000000000	3.038918000	1.449880000
O	-2.457924000	0.000000000	2.360968000
O	-0.000000000	-3.038918000	1.449880000
O	2.457924000	-0.000000000	2.360968000
O	-0.000000000	-2.457924000	-2.360968000
O	-3.038918000	-0.000000000	-1.449880000

Ti	0.000000000	0.000000000	0.000000000

$Ti(CO)_7 \cdots CO^+$ (C_s, $^2A'$)

E = -1755.767478 au

C	-2.352308000	0.903881000	-0.000000000
C	-0.293631000	2.001134000	1.535073000
C	1.915089000	0.644942000	0.000000000
C	-0.293631000	2.001134000	-1.535073000
C	0.265289000	-0.607495000	1.859163000
C	0.265289000	-0.607495000	-1.859163000
O	3.033209000	0.759672000	0.000000000
O	0.564805000	-1.095861000	2.828199000
O	-3.442791000	1.184609000	-0.000000000
O	-0.293631000	2.799780000	-2.321731000
O	0.564805000	-1.095861000	-2.828199000
O	-0.293631000	2.799780000	2.321731000
C	-1.364470000	-1.462542000	-0.000000000
O	-1.946417000	-2.427088000	-0.000000000
C	1.880540000	-2.963392000	0.000000000
O	2.481058000	-3.907480000	0.000000000
Ti	-0.248739000	0.381754000	-0.000000000

$Ti(CO)_6 \cdots 2CO^+$ (C_{2v}, $^4B_1$)

E = -1755.771622 au

C	-1.613900000	1.412796000	0.575963000
C	-1.613900000	-1.412796000	0.575963000
C	1.613900000	-1.412796000	0.575963000
C	-0.000000000	-0.000000000	2.692782000
C	0.000000000	0.000000000	-1.541003000
C	1.613900000	1.412796000	0.575963000
O	2.481127000	-2.144251000	0.574179000
O	0.000000000	0.000000000	-2.679297000
O	-2.481127000	2.144251000	0.574179000
O	-0.000000000	-0.000000000	3.830282000
O	2.481127000	2.144251000	0.574179000
O	-2.481127000	-2.144251000	0.574179000
C	0.000000000	3.497655000	-1.730587000
O	0.000000000	4.301975000	-2.513211000
Ti	-0.000000000	-0.000000000	0.575593000
C	-0.000000000	-3.497655000	-1.730587000
O	-0.000000000	-4.301975000	-2.513211000

$Zr(CO)_8^+$ (D_{4h}, $^2A_{1g}$)

E = -953.379853 au

C	0.000000000	1.978005000	1.362332000
C	1.978005000	-0.000000000	1.362332000
C	0.000000000	1.978005000	-1.362332000
C	-0.000000000	-1.978005000	-1.362332000
C	-1.978005000	0.000000000	-1.362332000
C	-1.978005000	-0.000000000	1.362332000
C	1.978005000	-0.000000000	-1.362332000
C	-0.000000000	-1.978005000	1.362332000
O	0.000000000	-2.909134000	-1.992040000
O	2.909134000	0.000000000	-1.992040000
O	2.909134000	-0.000000000	1.992040000
O	0.000000000	2.909134000	1.992040000
O	-2.909134000	0.000000000	1.992040000
O	-0.000000000	-2.909134000	1.992040000
O	-2.909134000	0.000000000	-1.992040000
O	0.000000000	2.909134000	-1.992040000
Zr	0.000000000	0.000000000	0.000000000

$Zr(CO)_7\cdots CO^+$ (C_s, $^2A'$)

E = -953.368828 au

C	1.611978000	2.092139000	0.000000000
C	-0.711444000	1.860659000	-1.760797000
C	-2.175756000	-0.512029000	-0.000000000
C	-0.711444000	1.860659000	1.760797000
C	0.101046000	-0.856846000	-1.959321000
C	0.101046000	-0.856846000	1.959321000
O	-3.201573000	-0.968877000	-0.000000000
O	0.103795000	-1.459525000	-2.909652000
O	2.374946000	2.920349000	0.000000000
O	-1.067707000	2.525965000	2.591126000
O	0.103795000	-1.459525000	2.909652000
O	-1.067707000	2.525965000	-2.591126000
C	2.077205000	-0.572333000	0.000000000
O	3.085917000	-1.074443000	0.000000000
Zr	0.000000000	0.415415000	0.000000000
C	-0.314652000	-3.561926000	-0.000000000
O	-0.313967000	-4.680843000	-0.000000000

$Zr(CO)_6\cdots 2CO^+$ (C_{2v}, $^4B_1$)

E = -953.352454 au

C 0.00000000 1.64013600 2.42786100<br>C -2.35231900 0.00000000 0.72917000<br>C 0.00000000 -1.64013600 2.42786100<br>C 2.13048800 0.00000000 -2.82860000<br>C 2.35231900 0.00000000 0.72917000<br>C 0.00000000 1.71936700 -0.86458300<br>C 0.00000000 -1.71936700 -0.86458300<br>C -2.13048800 0.00000000 -2.82860000<br>O 2.69877000 0.00000000 -3.79292700<br>O 0.00000000 -2.55055600 -1.61995800<br>O -3.47533600 0.00000000 0.70715700<br>O 0.00000000 2.41574100 3.24063300<br>O 0.00000000 2.55055600 -1.61995800<br>O -2.69877000 0.00000000 -3.79292700<br>O 3.47533600 0.00000000 0.70715700<br>O 0.00000000 -2.41574100 3.24063300<br>Zr 0.00000000 0.00000000 0.74688300
$Hf(CO)_8^+$ (D_{4h}, $^2A_{1g}$)<br><br>E = -954.344201 au<br><br>C 0.00000000 1.96356400 1.35173100<br>C 1.96356400 0.00000000 1.35173100<br>C 0.00000000 1.96356400 -1.35173100<br>C 0.00000000 -1.96356400 -1.35173100<br>C -1.96356400 0.00000000 -1.35173100<br>C -1.96356400 0.00000000 1.35173100<br>C 1.96356400 0.00000000 -1.35173100<br>C 0.00000000 -1.96356400 1.35173100<br>O 0.00000000 -2.89375500 -1.98434600<br>O 2.89375500 0.00000000 -1.98434600<br>O 2.89375500 0.00000000 1.98434600<br>O 0.00000000 2.89375500 1.98434600<br>O -2.89375500 0.00000000 1.98434600<br>O 0.00000000 -2.89375500 1.98434600<br>O -2.89375500 0.00000000 -1.98434600<br>O 0.00000000 2.89375500 -1.98434600<br>Hf 0.00000000 0.00000000 0.00000000
$Hf(CO)_7\cdots CO^+$ (C_s, $^2A'$)<br><br>E = -954.333873 au

C 1.48314200 2.10228300 0.00000000<br>C -0.79017400 1.71213300 -1.74771900<br>C -2.13040000 -0.64099500 0.00000000<br>C -0.79017400 1.71213300 1.74771900<br>C 0.16456300 -0.92468600 -1.94279500<br>C 0.16456300 -0.92468600 1.94279500<br>O -3.14462400 -1.12448600 0.00000000<br>O 0.20055000 -1.53073200 -2.89122100<br>O 2.18721600 2.98232400 0.00000000<br>O -1.19185700 2.35567600 2.57579200<br>O 0.20055000 -1.53073200 2.89122100<br>O -1.19185700 2.35567600 -2.57579200<br>C 2.12626300 -0.51445400 0.00000000<br>O 3.16397600 -0.95536800 0.00000000<br>Hf 0.00000000 0.33490400 0.00000000<br>C -0.24974200 -3.62541800 0.00000000<br>O -0.20124000 -4.74320000 0.00000000
$Hf(CO)_6\cdots2CO^+$ (C_{2v}, $^4B_1$)<br><br>E = -954.314586 au<br><br>C -1.61975000 0.00000000 -2.27701800<br>C 0.00000000 2.32731800 -0.59764900<br>C 1.61975000 0.00000000 -2.27701800<br>C 0.00000000 -2.12889500 2.94361400<br>C 0.00000000 -2.32731800 -0.59764900<br>C -1.70828700 0.00000000 0.97503700<br>C 1.70828700 0.00000000 0.97503700<br>C 0.00000000 2.12889500 2.94361400<br>O 0.00000000 -2.69414800 3.90966600<br>O 2.54231800 0.00000000 1.72896100<br>O 0.00000000 3.45149900 -0.58178400<br>O -2.39347700 0.00000000 -3.09314100<br>O -2.54231800 0.00000000 1.72896100<br>O 0.00000000 2.69414800 3.90966600<br>O 0.00000000 -3.45149900 -0.58178400<br>O 2.39347700 0.00000000 -3.09314100<br>Hf 0.00000000 0.00000000 -0.61080900
$Ti(CO)_7^+$ (C_s, $^2A'$)<br><br>E = -1642.458607 au<br><br>C 0.240550000 -1.222122000 1.801604000

C	2.178677000	-0.269179000	0.000000000
C	0.399746000	1.469731000	-1.593616000
C	0.399746000	1.469731000	1.593616000
C	0.240550000	-1.222122000	-1.801604000
C	-1.818133000	1.154766000	-0.000000000
O	0.625542000	2.198205000	-2.415967000
O	0.399746000	-1.813875000	-2.744919000
O	0.399746000	-1.813875000	2.744919000
O	0.625542000	2.198205000	2.415967000
O	-2.755781000	1.777882000	-0.000000000
O	3.294091000	-0.397769000	0.000000000
C	-1.679895000	-1.377451000	-0.000000000
O	-2.559631000	-2.080816000	-0.000000000
Ti	-0.000067000	-0.025626000	0.000000000
$Zr(CO)_7^+$ (C_s, $^2A'$)			
E = -840.059941 au			
C	0.633554000	-1.260398000	1.882037000
C	2.352833000	0.474688000	-0.000000000
C	-0.059461000	1.520809000	-1.825003000
C	-0.059461000	1.520809000	1.825003000
C	0.633554000	-1.260398000	-1.882037000
C	-2.163148000	0.781688000	0.000000000
O	-0.059461000	2.221934000	-2.702155000
O	0.956402000	-1.834405000	-2.793176000
O	0.956402000	-1.834405000	2.793176000
O	-0.059461000	2.221934000	2.702155000
O	-3.216313000	1.179684000	0.000000000
O	3.453163000	0.694348000	-0.000000000
C	-1.411767000	-1.796160000	0.000000000
O	-2.101070000	-2.686738000	0.000000000
Zr	0.025152000	0.010374000	-0.000000000
$Hf(CO)_7^+$ (C_{2v}, $^2A_1$)			
E = -841.024784 au			
C	1.837703000	1.418092000	0.309648000
C	-0.000000000	-0.000000000	2.367501000
C	-1.837703000	-1.418092000	0.309648000
C	1.837703000	-1.418092000	0.309648000
C	-1.837703000	1.418092000	0.309648000
C	0.000000000	-1.338110000	-1.854360000

O	-2.729812000	-2.079451000	0.486104000
O	-2.729812000	2.079451000	0.486104000
O	2.729812000	2.079451000	0.486104000
O	2.729812000	-2.079451000	0.486104000
O	0.000000000	-2.012214000	-2.757218000
O	-0.000000000	-0.000000000	3.490261000
C	0.000000000	1.338110000	-1.854360000
O	0.000000000	2.012214000	-2.757218000
Hf	-0.000000000	-0.000000000	0.017414000

$Ti(CO)_6\cdots CO^+$ (C_{3v}, $^4A_1$)

E = -1642.463797 au

C	0.000000000	1.811676000	-0.709768000
C	-1.561585000	0.901581000	1.837551000
C	-0.000000000	-1.803163000	1.837551000
C	1.561585000	0.901581000	1.837551000
C	-1.568958000	-0.905838000	-0.709768000
C	1.568958000	-0.905838000	-0.709768000
O	-0.000000000	-2.718439000	2.485437000
O	-2.367864000	-1.367087000	-1.348423000
O	0.000000000	2.734174000	-1.348423000
O	2.354237000	1.359220000	2.485437000
O	2.367864000	-1.367087000	-1.348423000
O	-2.354237000	1.359220000	2.485437000
C	0.000000000	-0.000000000	-3.637597000
O	0.000000000	-0.000000000	-4.756782000
Ti	-0.000000000	0.000000000	0.559454000

$Zr(CO)_6\cdots CO^+$ (C_{3v}, $^4A_1$)

E = -840.044238 au

C	0.000000000	1.935478000	0.861875000
C	1.658128000	0.957321000	-1.845021000
C	0.000000000	-1.914641000	-1.845021000
C	-1.658128000	0.957321000	-1.845021000
C	1.676174000	-0.967739000	0.861875000
C	-1.676174000	-0.967739000	0.861875000
O	0.000000000	-2.826001000	-2.501050000
O	2.476217000	-1.429645000	1.500116000
O	-0.000000000	2.859289000	1.500116000
O	-2.447389000	1.413001000	-2.501050000

O	-2.476217000	-1.429645000	1.500116000
O	2.447389000	1.413001000	-2.501050000
C	0.000000000	0.000000000	3.710370000
O	0.000000000	0.000000000	4.829574000
Zr	0.000000000	0.000000000	-0.479494000

$Hf(CO)_6\cdots CO^+$ (C_{3v}, $^4A_1$)

E = -841.006187 au

C	-0.000000000	1.916711000	-0.936213000
C	-1.640815000	0.947325000	1.737552000
C	-0.000000000	-1.894650000	1.737552000
C	1.640815000	0.947325000	1.737552000
C	-1.659921000	-0.958356000	-0.936213000
C	1.659921000	-0.958356000	-0.936213000
O	0.000000000	-2.806575000	2.395097000
O	-2.461336000	-1.421053000	-1.573835000
O	0.000000000	2.842106000	-1.573835000
O	2.430565000	1.403288000	2.395097000
O	2.461336000	-1.421053000	-1.573835000
O	-2.430565000	1.403288000	2.395097000
C	0.000000000	0.000000000	-3.787767000
O	0.000000000	0.000000000	-4.906937000
Hf	0.000000000	0.000000000	0.386774000

$Ti(CO)_6^+$ (O_h, $^4A_{1g}$)

E = -1529.156322 au

C	0.000000000	0.000000000	2.211637000
C	0.000000000	2.211637000	-0.000000000
C	-0.000000000	-0.000000000	-2.211637000
C	-2.211637000	0.000000000	-0.000000000
C	2.211637000	0.000000000	0.000000000
C	0.000000000	-2.211637000	0.000000000
O	-0.000000000	-0.000000000	-3.333079000
O	3.333079000	-0.000000000	0.000000000
O	0.000000000	0.000000000	3.333079000
O	-3.333079000	-0.000000000	-0.000000000
O	-0.000000000	-3.333079000	0.000000000
O	-0.000000000	3.333079000	-0.000000000
Ti	0.000000000	0.000000000	0.000000000

$Zr(CO)_6^+$ (O_h, $^4A_{1g}$)

E = -726.736424 au

C	-0.000000000	-0.000000000	2.353059000
C	-0.000000000	2.353059000	-0.000000000
C	-0.000000000	-0.000000000	-2.353059000
C	-2.353059000	-0.000000000	-0.000000000
C	2.353059000	-0.000000000	0.000000000
C	-0.000000000	-2.353059000	0.000000000
O	0.000000000	0.000000000	-3.475613000
O	3.475613000	0.000000000	0.000000000
O	0.000000000	0.000000000	3.475613000
O	-3.475613000	0.000000000	-0.000000000
O	0.000000000	-3.475613000	0.000000000
O	0.000000000	3.475613000	-0.000000000
Zr	0.000000000	0.000000000	0.000000000

$Hf(CO)_6^+$ (O_h, $^4A_{1g}$)

E = -727.698144 au

C	0.000000000	0.000000000	2.328643000
C	0.000000000	2.328643000	-0.000000000
C	-0.000000000	-0.000000000	-2.328643000
C	-2.328643000	0.000000000	-0.000000000
C	2.328643000	0.000000000	0.000000000
C	0.000000000	-2.328643000	0.000000000
O	-0.000000000	-0.000000000	-3.452383000
O	3.452383000	-0.000000000	0.000000000
O	0.000000000	0.000000000	3.452383000
O	-3.452383000	-0.000000000	-0.000000000
O	-0.000000000	-3.452383000	0.000000000
O	-0.000000000	3.452383000	-0.000000000
Hf	0.000000000	0.000000000	0.000000000

$Ti(CO)_6^+$ (C_{2h}, ^2A_g)

E = -1529.124472 au

C	-2.169010000	0.243194000	0.000000000
C	0.036882000	1.399867000	1.689298000
C	2.169010000	-0.243194000	0.000000000
C	0.036882000	1.399867000	-1.689298000
C	-0.036882000	-1.399867000	1.689298000
C	-0.036882000	-1.399867000	-1.689298000

O	3.282711000	-0.391215000	-0.000000000
O	-0.036882000	-2.078982000	2.582964000
O	-3.282711000	0.391215000	-0.000000000
O	0.036882000	2.078982000	-2.582964000
O	-0.036882000	-2.078982000	-2.582964000
O	0.036882000	2.078982000	2.582964000
Ti	0.000000000	0.000000000	0.000000000

$Zr(CO)_6^+$ (C_{2v}, $^2B_1$)

E = -726.720127 au

C -1.52188500 1.73498300 0.70312000
C 1.52188500 1.73498300 0.70312000
C 1.36676500 0.00000000 -1.53180900
C -1.36676500 0.00000000 -1.53180900
C 1.52188500 -1.73498300 0.70312000
C -1.52188500 -1.73498300 0.70312000
O 2.06332800 0.00000000 -2.41815800
O 2.26177700 -2.55725800 0.89999400
O -2.26177700 2.55725800 0.89999400
O -2.06332800 0.00000000 -2.41815800
O -2.26177700 -2.55725800 0.89999400
O 2.26177700 2.55725800 0.89999400
Zr 0.00000000 0.00000000 0.28495300

$Hf(CO)_6^+$ (C_{2v}, $^2B_1$)

E = -727.682125 au

C 1.68379800 1.52379500 0.67267600
C 1.68379800 -1.52379500 0.67267600
C 0.00000000 -1.36757400 -1.60293200
C 0.00000000 1.36757400 -1.60293200
C -1.68379800 -1.52379500 0.67267600
C -1.68379800 1.52379500 0.67267600
O 0.00000000 -2.05872900 -2.49473300
O -2.49526000 -2.26731100 0.90294600
O 2.49526000 2.26731100 0.90294600
O 0.00000000 2.05872900 -2.49473300
O -2.49526000 2.26731100 0.90294600
O 2.49526000 -2.26731100 0.90294600
Hf 0.00000000 0.00000000 0.19600600
